# Seasonal Changes in the Seminal Plasma Proteome of the Crab-Eating Fox (*Cerdocyon thous*)

**DOI:** 10.1021/acs.jproteome.5c00694

**Published:** 2025-12-31

**Authors:** Jaqueline Candido Carvalho, Marcos Gomides Carvalho, Viviane M. Codognoto, Laiza Sartori Camargo, Ramanathan Kasimanickam, John Kastelic, Fabiana Ferreira de Souza, João Carlos Pinheiro Ferreira

**Affiliations:** † Department of Veterinary Surgery and Animal Reproduction, São Paulo State University, School of Veterinary Medicine and Animal Science, Rua Prof. Doutor Walter Mauricio Correa, s/n, Botucatu, São Paulo 18618-681, Brazil; ‡ College of Veterinary Medicine, 70739 Washington State University, 110 Ott Rd, Pullman, Washington 99163, United States; § Department of Production Animal Health, Faculty of Veterinary Medicine, 2129 University of Calgary, 3280 Hospital Dr NW, Calgary, Alberta T2N 4Z6, Canada

**Keywords:** biomarker, wild animal, canid, semen, seasonality, reproduction

## Abstract

The objective was to analyze seasonal changes in the seminal plasma proteome of crab-eating fox (*Cerdocyon thous*). Semen was collected in Brazil from March 2021 to March 2022 from five healthy adult males housed individually. Collections were performed without chemical or physical restraint by digital manipulation of the penis, and seminal plasma proteomics were assessed by mass spectrometry (ESI Q-Tof MS/MS) on 43 ejaculates from the reproductive season and four from the nonreproductive season. A total of 408 proteins were identified: 219 exclusives to the reproductive season (June–September), 90 to the nonreproductive season (October–May), and 99 shared between both. Protein abundance differed significantly between seasons. Proteins related to enzymatic and oxidoreductase functions predominated in the nonreproductive season, whereas those linked to sperm metabolism and reproductive processes were more abundant in the reproductive season. Among these, olfactory receptor, strawberry notch homologue, and zinc finger protein were considered potential reproductive season biomarkers, with AUC > 0.80 in the receiver operating characteristic analysis. This is the first study describing the seminal plasma proteome and its seasonal variation in the crab-eating fox, identifying biomarkers with potential applications in conservation and reproductive management of this and other endangered canids.

## Graphical abstract

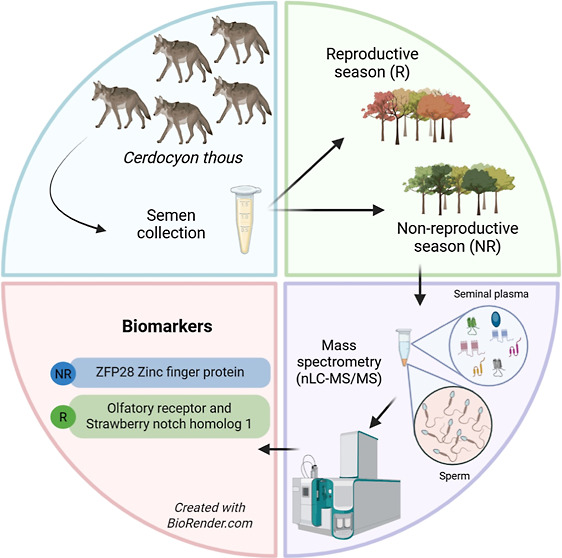

## Introduction

The *Cerdocyon thous* (crab-eating fox) is a medium-sized canid endemic to South America, with wide distribution across various ecosystems in Brazil and neighboring countries.^1,2^ Although it is currently classified as “Least Concern” by the International Union for Conservation of Nature (IUCN Red List), the last population assessment was conducted in 2015.^3^ The 10 year gap is a critical lack of updated data, limiting an accurate assessment of the actual conservation status.

Crab-eating fox are frequently exposed to infectious agents that are relatively common in domestic dogs, including *Rickettsia parkeri* rickettsiosis,^4^ canine distemper virus (CDV), parvovirus,^5^ and rabies.^6^ Peri-urban areas and landscape modifications increase their proximity to domestic dogs that may be infected.^7,8^ Furthermore, they have been identified as the most frequently road-killed mammal on Brazilian highways,^9,10^ highlighting their high vulnerability to anthropogenic threats, likely a consequence of their generalist habits and increasing overlap between their home ranges and urbanized areas.^1^

Reproductive seasonality synchronizes parturition with periods of greater resource availability, ensuring births occur under conditions favorable to offspring survival and healthy neonatal development.^11–13^ This adaptive trait enhances reproductive success in natural environments and is well documented in domestic species,^13–15^ wild canids,^15–17^ and wild felids.^18–20^ Observational and interventional studies suggest that the crab-eating fox is a long-day breeder, with a breeding season in the late winter and spring.^1,21,22^ Supporting this, females display seasonal estrous cycles during winter and early spring,^23^ whereas males have marked increases in semen volume, sperm motility, total sperm count, concentration, and proportion of morphologically normal sperm during the breeding season.^24^ Despite behavioral and limited physiological insights, the lack of molecular data remains a major gap in understanding the species’ seasonal reproductive biology.

Seminal plasma proteins have enzymatic and structural roles that support subcellular functions^25^ and influence sperm energy, metabolism, motility, capacitation, acrosome reaction, and cryoresistance^26–28^ processes that are essential for fertilization.^29^ They also protect sperm from polymorphonuclear neutrophils and provide antimicrobial and antioxidant defense.^30^ In domestic canids, proteins related to sperm motility, antioxidant protection, and immune regulation have been identified, underscoring their importance for reproductive functions.^25,31,32^ However, little is known about the seminal plasma proteome of wild canids,^33^ particularly neotropical species with seasonal reproductive patterns, such as the crab-eating fox (*C. thous*). Characterizing molecular composition of seminal plasma in these species could provide valuable insights for reproductive monitoring, fertility assessment, and conservation strategies.^33^ Moreover, identifying these proteins and their seasonal abundance patterns could provide evidence to establish reliable fertility biomarkers.^34,35^

Effects of seasonality on the seminal plasma proteome are inconsistent across the species. Significant seasonal proteome variations have been reported in bulls, boar, nonhuman primates, buffalo, pigs, goats, and horses,^34–41^ whereas others reported no significant variation in horses and cattle.^42,43^ These contrasting findings underscore the need for species-specific investigations, particularly in wild animals with distinct reproductive adaptations. Impacts of seasonality on the seminal plasma proteome of wild canids has apparently not yet been reported and may provide valuable insights into optimal breeding periods, particularly for ex situ populations.

Our objectives were to characterize seasonal variations in the seminal plasma proteome of male crab-eating fox using mass spectrometry and quantitative proteomics, to deepen our understanding of its reproductive physiology, and to provide new knowledge to inform future conservation strategies. We hypothesized that the seminal plasma proteome has significant seasonal differences, reflecting species-specific reproductive adaptations.

## Experimental Procedures

### Reagents

All reagents used were of the highest purity and were obtained from Sigma-Aldrich (St. Louis, MO, USA), GE Healthcare Life Sciences (São Paulo, SP, Brazil), Waters Corporation (Milford, MA, USA), Bio-Rad (São Paulo, SP, Brazil), and Thermo Fisher Scientific (São Paulo, SP, Brazil) or as mentioned otherwise.

### Ethics Statement

This study was conducted in accordance with the National Council for the Control of Animal experimentation (CONCEA—Brazil) and was approved by the Brazilian Institute of the Environment and Renewable Natural Resources (permit number 77730.1-SISBIO) and by the Ethics Committee of Animal Use of the FMVZ—UNESP (Permit 0134/2021-CEUA).

### Animals and Management

This study was conducted from March 2021 to March 2022 in the southern hemisphere (22° 53′ 08″ S; 48° 26′ 42″ W) at the Center for Medical Research and Wild Animals (CEMPAS)—School of Veterinary Medicine and Animal Science (FMVZ)—São Paulo State University (UNESP—Brazil).

This region has a tropical Aw climate according to the Köppen classification, characterized by a hot, rainy summer and a mild, dry winter. Annual precipitation ranges from 1500 to 1700 mm, with the majority occurring between October and March, particularly from December to February, when monthly totals often exceed 200 mm.^44,45^ The dry season extends from April to September, with July being the driest month (<50 mm precipitation).^44,45^ Average daytime temperatures range from 26–27 °C in summer to 22–23 °C in winter, whereas average nighttime temperatures range from 19–20 °C in summer to 12–14 °C in winter, resulting in an annual mean of approximately 20–21 °C.^44,45^ The day length follows subtropical latitude patterns, varying from approximately 10 h 40 min at the winter solstice (June) to 13 h 30 min at the summer solstice (December), with intermediate values near 12 h at the equinoxes (March and September), which also mark the formal onset of the respective seasons.^46^ The Köppen classification of the region has been updated according to global climate mapping studies.^45^

Five healthy crab-eating fox males were used, with an average age of 3.7 ± 0.97 (range 1.0–5.5) years old and an average weight of 6.7 kg ± 0.2 (range 6.3–7.2 kg), previously rescued as puppies or young adults.

During the experimental period, all males were housed individually in separate cages that allowed visual contact with one another. No females were kept in adjacent enclosures or within the visual range. The foxes were fed commercial pelleted dog food (FMVZ, UNESP—Botucatu, SP, Brazil), supplemented with fruits, fresh meat, viscera, and water ad libitum.

The interval from June to September was considered the reproductive season (R), whereas the remaining months were classified as the nonreproductive season (NR).^24^

### Animal Conditioning and Semen Collection

The animals had been conditioned to allow semen collection by digital manipulation of the penis using a positive reinforcement protocol, as described by Carvalho et al.^47^ Conditioning was conducted between July 2018 and June 2019 and involved gradual habituation to the human presence and physical contact, following a structured routine adapted to the species. No chemical or physical restraint was used to facilitate the semen collection.

Thirty-eight ejaculates from the R season and 36 from the NR season were selected for semen analysis. Ejaculate volume was measured using an automatic micropipet, and sperm motility was assessed by phase-contrast microscopy (400× magnification; Leica, Leica Microsystems, Wetzlar, Germany). Semen samples were diluted (1:10) in formalin-saline, and sperm concentration was determined using a Neubauer chamber under light microscopy (400× magnification). For sperm morphology assessment, wet mounts were prepared by diluting semen in warm formalin-saline, placing a drop on a microscope slide, and covering it with a coverslip. Samples were examined using differential interference contrast microscopy (1000× magnification; Leica, Leica Microsystems). A total of 100 sperm cells were classified as morphologically normal or abnormal,^47^ and results were expressed as percentages of normal sperm and sperm with minor or major defects.

### Seminal Plasma Proteomics

Forty-three ejaculates from the R season and four from the NR season were selected for proteomic analysis, as they exceeded 50 μL in volume. After semen collection, seminal plasma was separated from sperm by centrifugation at 800*g* for 15 min. Then, the supernatant (seminal plasma) was recentrifuged at 10,000*g* for 30 min at 4 °C to remove any residual cells, with the recovered supernatant stored at −80 °C.

The total protein concentration in seminal plasma was measured using a nanospectrophotometer at A280 (NanoDrop A280, Thermo Scientific NanoDrop One Microvolume UV–vis Spectrophotometers, Waltham, MA, USA). Then, an aliquot with 50 μg of total protein from each sample was run on a 12% SDS-PAGE gel (Bio-Rad Laboratories, USA), with each sample loaded into a separate lane. The run was stopped after the samples had entered the separation gel. The gel was stained with colloidal blue Coomassie.^48,49^ Single bands that had formed were excised and submitted to the tryptic digestion of proteins.

Digestion was performed as described by Shevchenko et al.^50^ with modifications. Briefly, gel fragments were initially destained 4× in an aqueous solution containing 50% methanol and 2.5% acetic acid. Thereafter, the fragments were dehydrated with 100% acetonitrile. Reduction of disulfide bonds was performed by using dithiothreitol and alkylation with iodoacetamide. Samples were digested overnight with 20 ng/μL trypsin (V5111, Sequencing grade Modified Trypsin, Promega, Madison, WI, USA) at a 1:50 trypsin/substrate ratio. Peptide extraction was performed with 5% formic acid. Samples were concentrated (SPD1010 Integrated SpeedVac Systems, Thermo Fisher Scientific Inc., Waltham, MA, USA) and stored at −80 °C until mass spectrometry analysis.

For mass spectrometry, samples were thawed, diluted in 0.1% formic acid at a proportion of 0.7 μg protein/μL, homogenized, and centrifuged at 1,100*g* for 5 min. Then, 20 μL of supernatant was deposited in glass tubes for analysis in the mass spectrometer (clear glass, 12 × 32 mm bolt neck total recovery vial with lid; Waters Corporation, Milford, MA, USA). An aliquot (4.5 μL) resulting from tryptic digestion of peptides was separated by an RP-nano UPLC C18 column (100 μm × 100 mm) (Waters nanoACQUITY UPLC, Waters Corporation) coupled to a quadrupole time-of-flight mass spectrometer (Q-TOF) (Micromass Q-Tof PREMIER Mass Spectrometer, Waters Corporation) with a nanoelectrospray source at a flow rate of 0.6 μL/min. A 2–90% acetonitrile gradient in 0.1% formic acid was maintained for 45 min. The nanoelectrospray voltage was maintained at 3.5 kV, the cone voltage at 30 V, and the source temperature at 100 °C. The instrument was operated in a positive top three mode, acquiring a mass spectrum (MS), followed by MS/MS of the three most intense peaks detected. After MS/MS fragmentation, the ion was kept on the exclusion list for 60 s, and the actual exclusion time was used to analyze endogenous cleavage of peptides.

Search parameters for spectral analysis were defined as follows: trypsin was specified as the proteolytic enzyme, allowing for a maximum of one missed cleavage; carbamidomethylation of cysteine residues was set as a fixed modification, whereas methionine oxidation was included as a variable modification. The mass tolerance was set to 1 Da for both precursor ions (MS) and fragment ions (MS/MS), and monoisotopic masses were used throughout the analysis.

Spectra were acquired using a MassLynx V. 4.1 software (Waters Corporation), and raw data files were converted to a list format (.mgf) without adding scans and then searched against the Mascot Distiller tool database MDRO 2.4.0.0 (Matrix Science Inc., Boston, MA, USA). For search, the *Canis lupus familiariz* taxonomy (UniProtKB: UP000805418) was used, as the UniProtKB database contained only 43 proteins (https://www.uniprot.org/), and the NCBI database (NCBI: ID962096; https://www.ncbi.nlm.nih.gov/) only 96 proteins deposited in the *C. thous* taxonomy. Relative quantification of each protein was determined using the exponentially modified protein abundance index (emPAI) obtained from Mascot Distiller software.^51^

### Statistical Analysis

Variables were normalized in Excel software; normalization was conducted using data of the proteins identified in at least 50% of the samples in each group (NR and R); otherwise, data were excluded. The emPAI value of each sample was divided by the sum of the protein emPAI of all samples within the groups, and the resulting value was used for statistical analysis.

Data were subjected to nonhierarchical clustering analysis using MetaboAnalyst 6.0 software.^52^ To validate group classifications and assess impacts of outliers on protein abundance, multivariate analysis—specifically principal component analysis (PCA)—was employed to characterize variation among samples within the score matrix. The heatmap was used to describe the distribution pattern of the main proteins in the seasons for visual comparison of proteins in the groups. Univariate analysis (Volcano plot) was also performed. The receiver operating characteristic (ROC) curve was calculated, and an AUC value > 0.8 was considered indicative of a strong discriminatory power. An FDR value < 0.05 and a log2 FC change ≥1.5 were considered significant.

A Venn diagram was created to illustrate the distribution profile of proteins across groups (http://bioinformatics.psb.ugent.be/). Gene ontology (GO) enrichment analysis was conducted using ShinyGO 0.82.^53^ The complete data set of identified proteins across seasons was considered in the analyses.

## Results

Seasons affected the ejaculate volume, total sperm count and total motility, with higher values during the R season (*P* < 0.05). However, no significant seasonal effect was detected for the concentration or sperm defects. Seminal characteristics during R and NR seasons are listed in Table 1.

**Table 1:** Mean ± SEM Ejaculates Characteristics of Crab-Eating Fox (*C. thous*) during Reproductive (Jun–Sep) and Nonreproductive Seasons (Oct–May)^a^

characteristic	reproductive (*n* = 38)	nonreproductive (*n* = 36)
volume (μL)	263.16 ± 37.68^a^	111.11 ± 31.50^b^
total motility (%)	37.3 ± 8.8^a^	16.0 ± 5.6^b^
concentration (× 10^6^/mL)	75.17 ± 9.06	96.62 ± 19.38
total sperm/ejaculates (× 10^9^)	25.58 ± 6.01^a^	12.11 ± 3.91^b^
major defects (%)	89.4 ± 3.6	85.5 ± 3.3
minor defects (%)	6.6 ± 1.6	12.3 ± 3.4

a ^a,b^Within a row, means without a common superscript differed (*P* < 0.05).

Proteomics analysis identified 408 proteins in seminal plasma, with 219 and 90 exclusively detected in R and NR seasons, respectively, and 99 conserved across both seasons (Figure 1). PCA identified 2 clusters, but there was an overlap between groups (PC1 + PC2 = 33%) (Figure 2).

**Figure 1:**
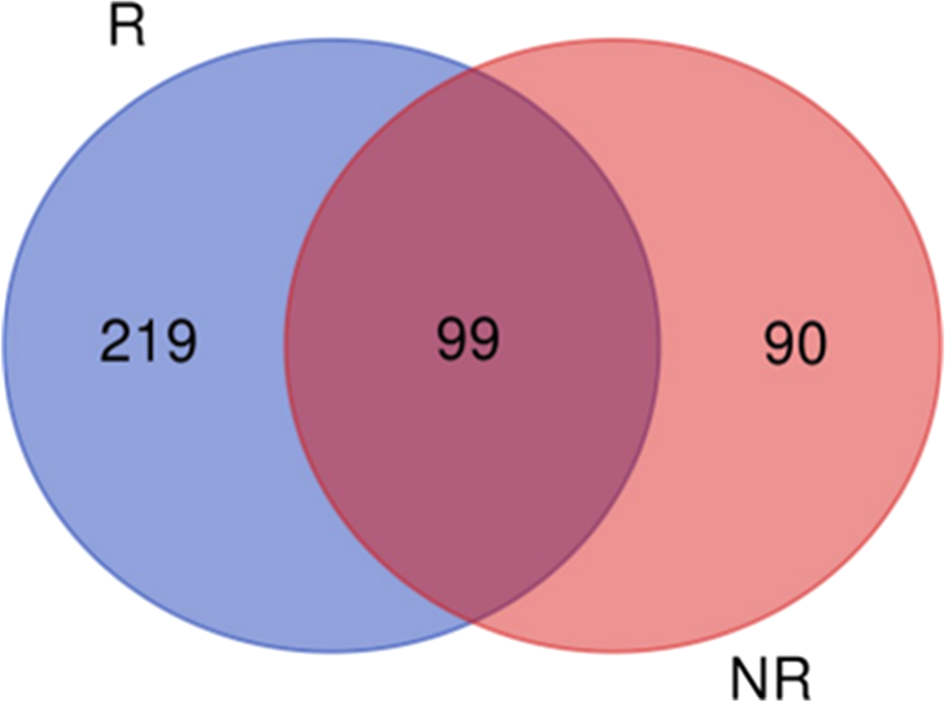
Venn diagram representing proteins detected in seminal plasma of *Cerdocyon thous* (*n* = 5) during reproductive (R) and nonreproductive (NR) seasons.

**Figure 2:**
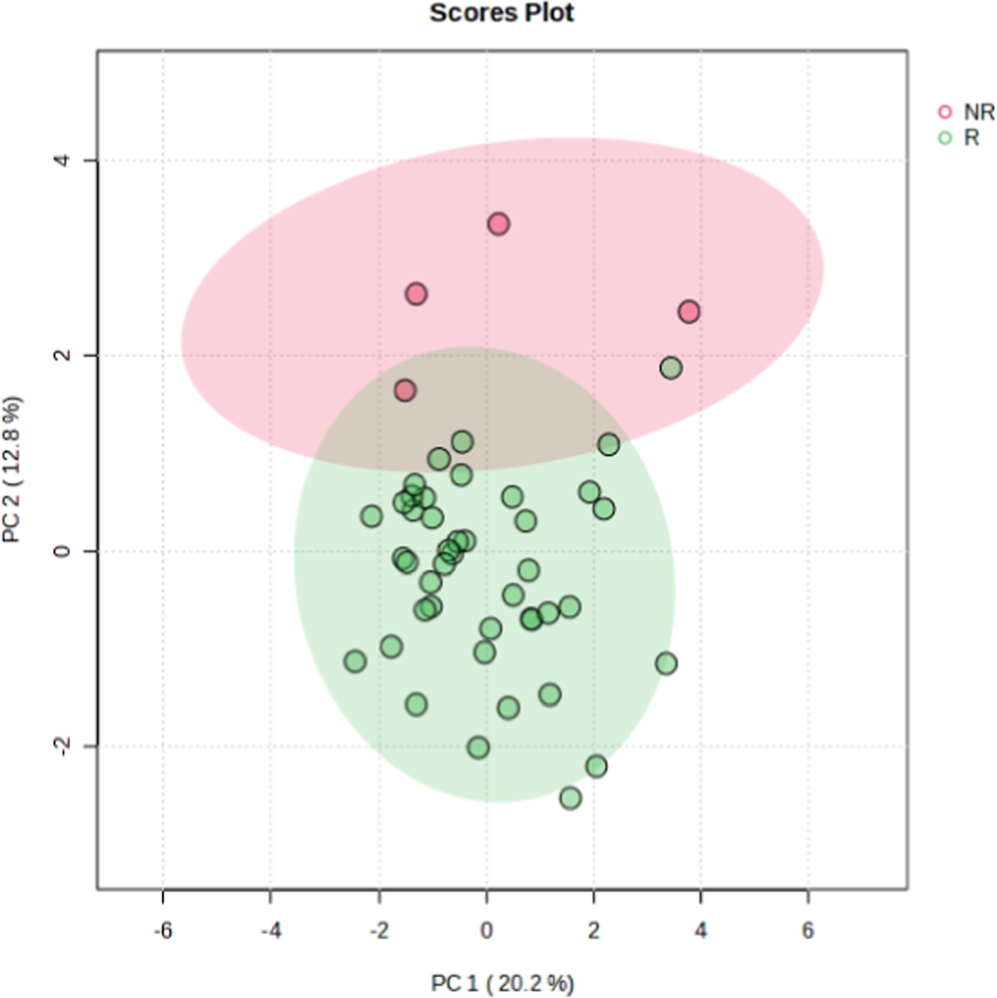
PCA of proteins in seminal plasma of *Cerdocyon thous* (*n* = 5) during reproductive (R; *n* = 43) and nonreproductive (NR; *n* = 4) seasons. Note that the sum of PC1 + PC2 was 33%.

Volcano plot analysis revealed distinct seasonal abundances, with 6 proteins in lower abundance and 2 in greater abundance during the R season (Figure 3). Moreover, 32 proteins were present in 80% of samples during the R season, whereas 12 were detected in 80% of NR samples (Figure 4).

**Figure 3:**
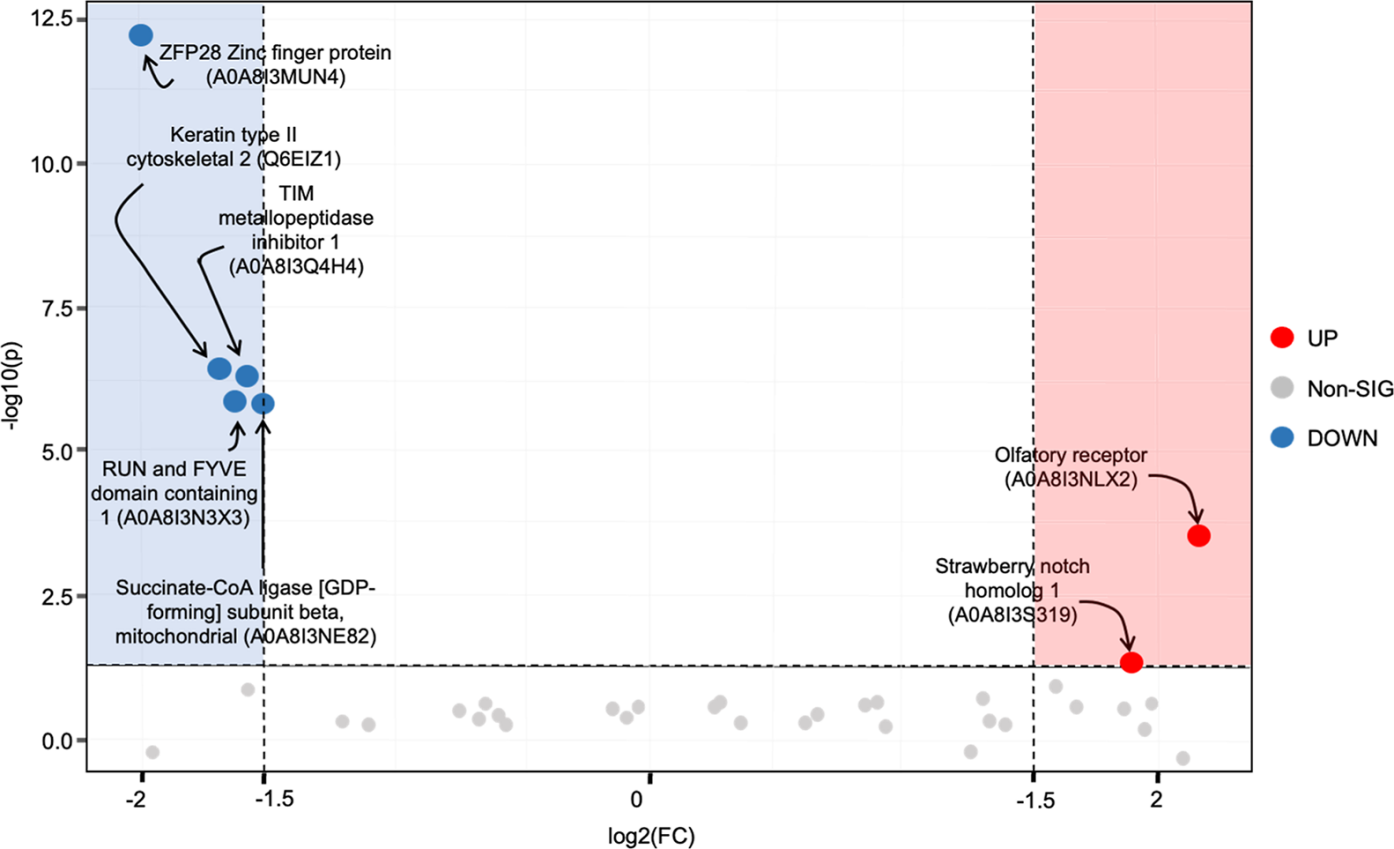
Volcano plot of proteins with lower (blue) and higher (red) abundances in seminal plasma of *Cerdocyon thous* (*n* = 5) during the breeding (R) compared to the nonbreeding (NR) season. FDR adjusted *p*-value < 0.05, log2 FC change ≥1.5.

**Figure 4:**
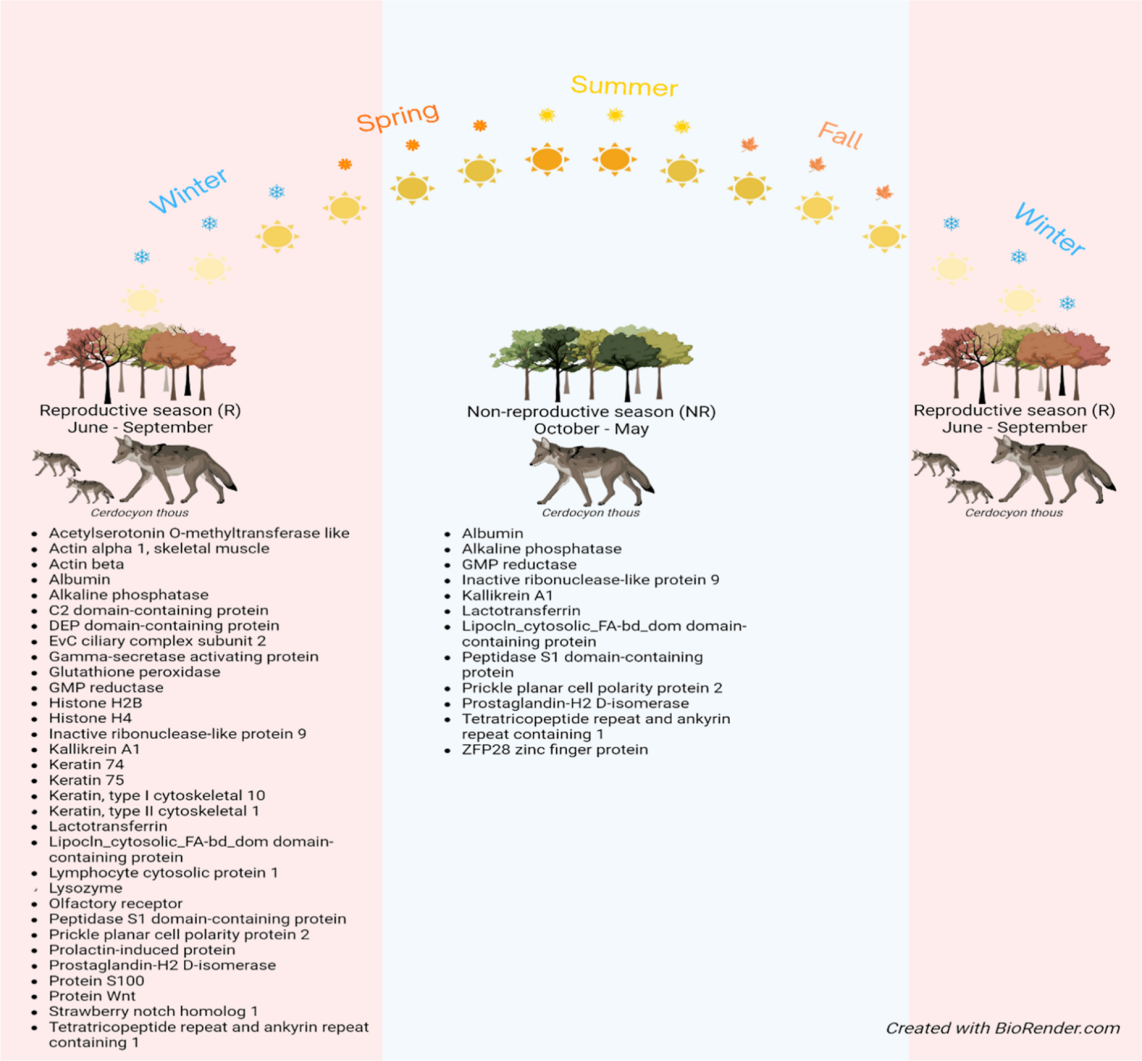
Proteins identified in at least 80% of seminal plasma samples collected during reproductive and nonreproductive seasons of the crab-eating fox.

Gene ontology enrichment analysis revealed that proteins identified in seminal plasma during the NR season were associated with enzymatic and oxidoreductase activities. In contrast, those detected in the R season were linked to biological process pathways (Figure 5).

**Figure 5:**
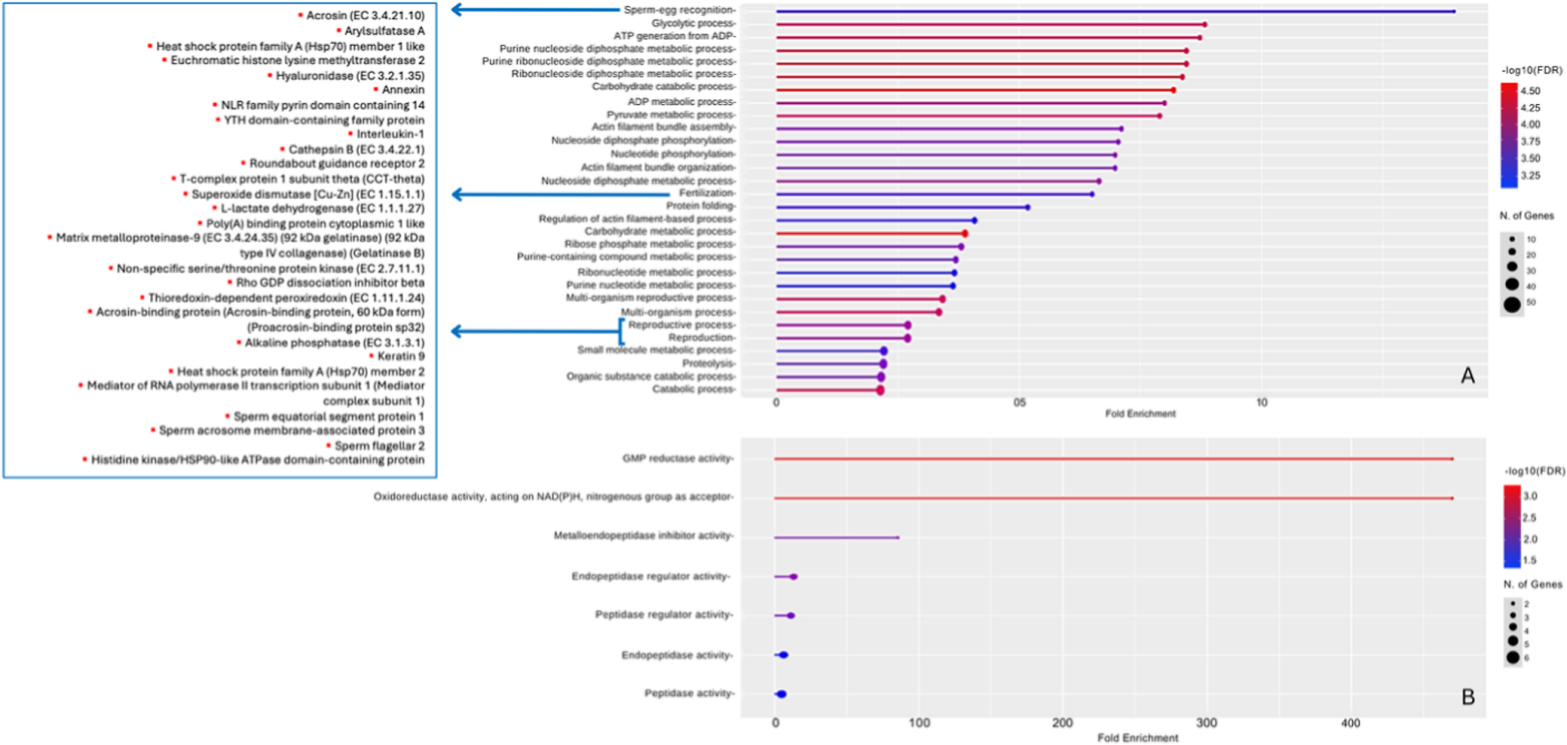
Gene ontology enrichment (biological process) of seminal plasma proteins from *C. thous* (*n* = 5) in R (A) and NR (B) seasons. Proteins associated with the reproduction process were highlighted in the R season.

The heatmap displaying the pattern of seminal plasma proteins from the R and NR seasons is shown in Figure 6. Proteomics results were subjected to biomarker analysis using an ROC curve; three proteins were identified with an area under the curve (AUC) > 0.80 (Figure 7).

**Figure 6:**
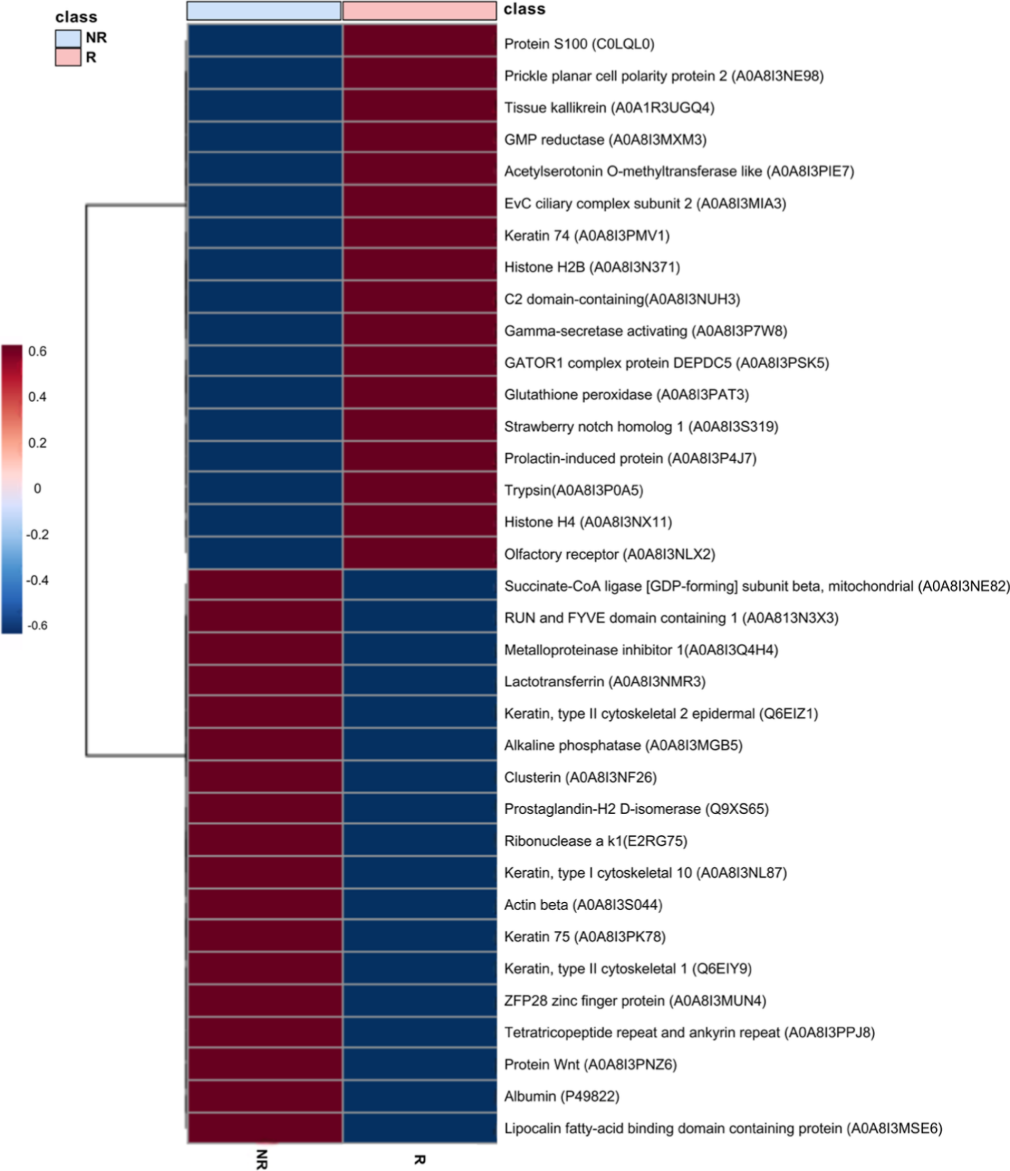
Heatmap of main proteins detected in seminal plasma of *Cerdocyon thous* (*n* = 5) during reproductive (R) and nonreproductive (NR) seasons.

**Figure 7:**
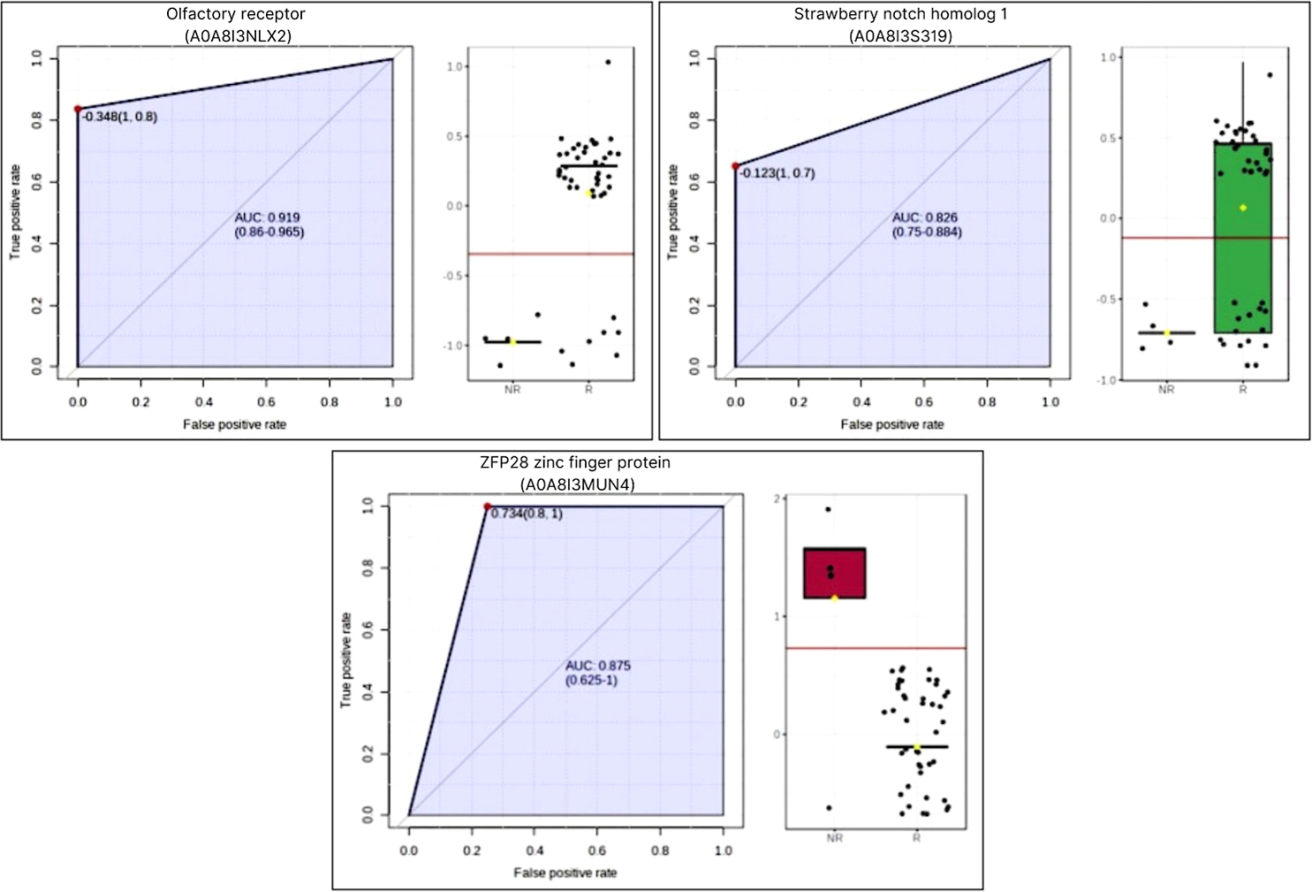
ROC curve and AUC (>0.80) of the quantitative analysis (emPAI) of seminal plasma proteins in *Cerdocyon thous* during reproductive (R) and nonreproductive (NR) seasons.

Gene ontology based on molecular function, biological process, and cellular component of proteins in the heat map (Figure 6) is detailed in Supporting Information Table S1. Additionally, genes involved in gene ontology (molecular function) enrichment during the NR season are listed in Supporting Information Table S2, whereas those for the R season are listed in Supporting Information Table S3. The ID mapping and gene ontology of proteins identified during the NR season are presented in Supporting Information Table S4, whereas those for the R season are listed in Supporting Information Table S5.

During the R season, proteins associated with cell signaling, molecular recognition, and regulation of sperm function were predominant. In contrast, the NR season had a higher abundance of proteins involved in cellular structural maintenance, immune response modulation, oxidative stress, and apoptotic processes, indicating a distinct functional proteomic profile across the reproductive cycle.

## Discussion

Greater seminal volume, sperm motility, and total sperm count during the R season reinforced the role of seasonality as a key determinant of semen quality in the crab-eating fox. The similarity of these findings with those reported by Silva et al. (2022)^24^ implied a consistent reproductive pattern for the species. Therefore, we inferred that proteomic and functional variations identified in the present study likely reflected physiological changes associated with the reproductive period rather than sampling variability.

To the best of our knowledge, this is the first study to characterize the seminal plasma proteome of the crab-eating fox and examine protein abundance changes across reproductive and nonreproductive seasons. Given the limited number of proteins reported for this species in the NCBI (96) and UniProtKB (43) databases, we used the *C. lupus familiaris*database as a reference (UniProtKB, https://www.uniprot.org/proteomes/UP000805418). This strategy enabled identification of 408 proteins, exceeding previously reported numbers for the seminal plasma of the domestic dogs (*C. lupus familiaris*).^25,32^ Moreover, they revealed conserved seminal plasma components among Canidae species, with the most abundant proteins closely resembling key seminal plasma proteins in domestic dogs, including alkaline phosphatase, albumin, lactotransferrin, and kallikrein A1. Notably, kallikrein A1 shared 100% sequence identity with arginine esterase, the predominant protein in canine seminal plasma.^54^ These findings offered valuable insights into functional roles of seminal plasma proteins in the crab-eating fox, particularly in the context of reproductive seasonality.

We identified the main proteins present in the seminal plasma of the crab-eating fox; during the nonreproductive season, they were primarily associated with enzymatic and oxidoreductase functions. In contrast, during the reproductive season, the proteins were mainly involved in sperm metabolism and reproductive functions. Despite a slight overlap in the proteins identified in seminal plasma from the R and NR seasons, DAPs were detected. Interestingly, in R season, the DAPs were the sperm olfactory receptor (OR), a member of the G-protein-coupled receptor (GPCR) superfamily^55^ involved in transduction of chemical signals into electrical impulses,^56^ with key roles in reproductive behavior, social interactions, mate selection, and partner recognition.^57,58^ They also modulate the reproductive cycle and are considered essential for pheromone recognition and, consequently, for survival and reproduction of wild canids.^59^

This study is the first to report the presence of olfactory receptors (ORs) in the seminal plasma of crab-eating fox. Increased abundance of these receptors during the reproductive season suggested a potential role for them as seminal biomarkers of the reproductive activity in this species. This finding highlighted their significance in signaling pathways that regulate sperm function, aligning with previous studies indicating that ORs in sperm can be activated by specific odorant molecules, triggering biochemical modifications essential for motility, maturation, and chemotactic response.^60^ These processes enhance sperm interaction with chemical signals released by the female during ovulation, ultimately facilitating fertilization.^56,61–64^ These mechanisms are not exclusive to domestic canids but have also been observed in wolves,^59^ suggesting an evolutionarily conserved function of these receptors across taxonomic groups.

Olfactory receptors (ORs) also interact with heparin and have a key role in sperm capacitation across several species, including rats,^62,65^ humans,^62,66^ and boars.^67^ Emerging evidence also suggests a link between OR mutations or altered expression and infertility, with potential implications for diagnostics and development of new contraceptive strategies.^68^

SNOB1, encoded by SBNO1, was more abundant during the R season. This nuclear protein regulates gene transcription and is linked to neural stem cell proliferation, neuroinflammatory response,^69^ and testicular development.^70^ This protein is expressed in testicular cells and participates in Notch- and Hippo-dependent regulatory networks, which suggests a potential contribution to pathways related to sperm maturation, cellular stress responses, and inflammatory signaling.^71–73^ Its increased abundance in seminal plasma during the reproductive season may reflect the broader seasonal modulation of regulatory pathways associated with reproductive function.

To the best of our knowledge, this is the first report of elevated levels of zinc finger protein 28 (Zfp28) in seminal plasma during the NR season of a seasonal species. Zfp28 is part of the zinc finger protein family, the largest group of transcription factors in the eukaryotic genome.^74,75^ It is involved in diverse processes, including development, reproduction, immunity, antioxidant defense, membrane stability,^75,76^ cell cycle regulation,^77^ and tumorigenesis.^78^ As a transcription factor predominantly located in the nucleus, its presence in seminal plasma was unexpected.^74^ Perhaps, there was an increase in epithelial cell apoptosis^79^ during the NR season.

The RUN and FYVE domain-containing protein 1 (RUFY1), which was upregulated during the NR season, is a member of the RUFY family. These proteins have essential roles in regulating endosomal trafficking, autophagy, cell migration, and cytoskeletal dynamics,^80,81^ processes crucial for cellular homeostasis and responses to environmental stimuli.^82^ During the NR season, when spermatogenic activity is reduced, the presence of RUFY1 in the ejaculate may be linked to compensatory mechanisms that regulate protein degradation and renewal, contributing to cellular integrity. Perhaps, RUFY1 supports tissue preservation during reproductive quiescence, helping to maintain testicular homeostasis until spermatogenic activity resumes.

Tissue inhibitor of metalloproteinase-1 (TIMP-1), which was upregulated during the nonreproductive (NR) season, has been detected in sperm tails,^83^ epididymal epithelial cells,^84^ and seminal plasma.^85^ Its function in this environment and its relationship with sperm quality remain poorly understood.^85^ One study associated higher seminal plasma concentrations of TIMPs (TIMP-1, TIMP-2, and TIMP-4) with increased sperm DNA fragmentation,^86^ a finding that appears expected given the higher prevalence of abnormal sperm during the NR season.^24^

Keratin, epidermal type II cytoskeletal 2 (KRT2), identified as a DAP in seminal plasma during the NR season, is involved in mitosis, stress responses, and protection against apoptosis.^87^ It has been reported as hypophosphorylated in asthenozoospermic sperm in men.^88^ Although phosphorylation was not evaluated here, such posttranslational modifications may contribute to the reduced sperm motility observed in the NR season.^15,87,89,90^

Clusterin, identified as a DAP in the seminal plasma of crab-eating fox during the NR season, has diverse roles including lipid transport, sperm maturation, endocrine regulation, apoptosis initiation, complement system modulation, membrane protection, cellular interactions, and tissue remodeling.^91,92^ It has also been reported in seminal plasma of wild mammals such as collared peccaries (*Pecari tajacu*)^93^ and ring-tailed coatis (*Nasua nasua*).^94^ Clusterin has been regarded as a biomarker of human sperm with low quality, as its quantity is increased in sperm^95^ and seminal plasma of asthenozoospermic men^96^ and in prostasomes of infertile men.^97^ Perhaps, clusterin was associated with the reduced sperm motility observed during the NR season.^98–102^

During the NR season, proteins such as zinc finger protein 28 (Zfp28), RUN and FYVE domain-containing protein 1 (RUFY1), tissue inhibitor of metalloproteinase-1 (TIMP-1), epidermal type II keratin (KRT2), and clusterin were present in higher abundance. These findings implicated active processes involved in recovery and maintenance of the reproductive tract, particularly related to immune modulation, apoptosis regulation, and structural cell integrity.

Although specific studies on the seminal plasma proteome of wild species remain limited, variations in semen characteristics have been reported in canids such as the red wolf (*Canis rufus* *)* ^103^ and coyote (*Canis latrans*)*,* ^99^ in partridges (*Rhynchotus rufescens*),^100^ and in the raccoon dog (*Nyctereutes procyonoides*).^101^ Biochemical variations have also been observed in wild small ruminants^104^ and in the Arctic fox (*Alopex lagopus* *)*,^105^ with seasonal changes in testosterone and prolactin concentrations directly affecting cryotolerance.

Considering the current results, proteins related to cell signaling, molecular recognition, and sperm function regulation were predominant during the R season. In contrast, during the NR season, proteins involved in structural maintenance, immune modulation, oxidative stress, and apoptosis were more abundant, indicating distinct functional proteomic profiles across the seasons. These findings provided new insights into molecular mechanisms underlying seasonal changes in the ejaculate and may contribute to understanding fertility regulation in the crab-eating fox.

## Conclusions

This study characterized the seminal plasma proteome of the crab-eating fox during the R and NR seasons, marking the first proteomic analysis of seminal plasma in a South American canid. We identified proteins, including olfactory receptors and zinc finger protein 28, with the potential as biomarkers of reproductive status. These findings should inform conservation strategies, especially for critically endangered species. We recommend that future studies also examine serum proteomes and their association with seminal plasma components, as this could improve the identification of reliable biomarkers and contribute to more effective and accessible reproductive management in wildlife conservation.

## Supplementary Material











## Data Availability

Data supporting the findings of this study are available in the Mendeley Data repository at the following link: https://data.mendeley.com/preview/jhztynd45v?a=18023a85-cdc5-4ea1-9974-611ffd1531b4

## Notes

Supplementary Table S1. Gene ontology of seminal plasma proteins detected in heatmap analysis of seminal plasma of *C. thous* (*n* = 5) (PDF)
Supplementary Table S2. Genes involved in gene ontology (molecular function) enrichment in nonreproductive (NR) season (PDF)
Supplementary Table S3. Genes involved in gene ontology (molecular function) enrichment in reproductive (R) season (PDF)
Supplementary Table S4. ID mapping and gene ontology of proteins found in the nonreproduction (NR) season (PDF)
Supplementary Table S5. ID mapping and gene ontology of proteins found in the reproductive (R) season (PDF)
