# Supplementary material for: Seasonal Changes in the Seminal Plasma Proteome of the Crab-Eating Fox (Cerdocyon thous)
Source: J Proteome Res. 2025 Dec 31;25(2):723–34. doi: 10.1021/acs.jproteome.5c00694 (PMC12888006; doi:10.1021/acs.jproteome.5c00694)
Supplement: Supplementary file 1 [file pr5c00694_si_001.pdf]

Seasonal changes in seminal plasma proteome of the crab-eating fox (*Cerdocyon thous*)

Jaqueline C. Carvalho[1], Marcos G. Carvalho[1], Viviane M. Codognoto[1], Laiza S. Camargo, Ramanathan K Kasimanickam[2], Fabiana F. Souza[1], João C. P. Ferreira[1]

[1]Department of Veterinary Surgery and Animal Reproduction, School of Veterinary Medicine and Animal Science, São Paulo State University, Botucatu, SP, Brazil

[2]College of Veterinary Medicine, Washington State University, Pullman, WA, USA

*fabiana.f.souza@unesp.br, joao.cp.ferreira@unesp.br

Table of Contents

1. **Table S1.** Supplementary Table 1 is provided as a separate .docx file
2. **Table S2.** Supplementary Table 2 is provided as a separate .docx file
3. **Table S3.** Supplementary Table 3 is provided as a separate .docx file
4. **Table S4.** Supplementary Table 4 is provided as a separate .docx file
5. **Table S5.** Supplementary Table 5 is provided as a separate .docx file

Table S1.

Gene ontology of the seminal plasma proteins observed in the heatmap analysis plasma seminal of the *Cerdocyon thous* (n = 5). NR: non-reproductive, R: reproductive.

UniProt ID*	Name	Gene ID	Gene ontology			Season	
			Biological process	Molecular function	Cellular component	NR	R
**A0A8I3PIE7**	**Acetylserotonin O-methyltransferase like**	**ASMTL**	**Methylation**	**Nucleoside triphosphate diphosphatase activity; o-methyltransferase activity**	**--**		↑
**A0A8I3S044**	**Actin beta**	**ACTB**	**--**	**--**	**--**	↑	
**P49822**	**Albumin**	**ALB**	**Cellular response to starvation; maintenance of mitochondrion**	**DNA binding; enterobactin binding; fatty acid binding; metal ion binding; pyridoxal**	**Cytoplasm; extracellular space;**	↑	

			location; negative regulation of apoptotic process	phosphate binding; toxic substance binding	protein- containing complex		
A0A8I3MGB5	Alkaline phosphatase	ALPL	Bone mineralization; calcium ion homeostasis; cellular homeostasis; cellular response to organic cyclic compound; developmental process involved in reproduction; endochondral ossification; futile creatine cycle; inhibition of non-skeletal tissue mineralization; phosphate ion homeostasis; positive regulation of cold- induced thermogenesis; pyridoxal phosphate metabolic process; response to antibiotic; response	Alkaline phosphatase activity; calcium ion binding; phosphoamidase activity; phosphoethanolamine phosphatase activity; pyridoxal phosphatase activity; pyrophosphatase activity	Extracellular matrix; extracellular membrane- bounded organelle; mitochondrial intermembrane space; mitochondrial membrane; plasma membrane	↑	

			**to sodium phosphate; response to vitamin B6; response to vitamin D**				
**A0A8I3NUH3**	**C2 domain-containing protein**	**DOC2B**	**Calcium ion-regulated exocytosis of neurotransmitter; calcium-dependent activation of synaptic vesicle fusion; positive regulation of insulin secretion; protein localization; spontaneous neurotransmitter secretion**	**Calcium ion binding; syntaxin binding**	**Cytoplasm; plasma membrane; presynapse**		↑
**A0A8I3NF26**	**Clusterin**	**CLU**	**--**	**Chaperone**	**Chromaffin granule; cytosol; endoplasmic reticulum; extracellular region; mitochondrial**	↑	

					**membrane; nucleus; perinuclear region of cytoplasm**		
**A0A8I3PSK5**	**DEP domain containing 5, GATOR1 subcomplex subunit**	**DEPDC5**	**Cellular response to amino acid starvation; intracellular signal transduction; negative regulation of TORC1 signaling**	**GTPase activator activity; protein-containing complex binding**	**Cul3-RING ubiquitin ligase complex; cytosol; GATOR1 complex; lysosomal membrane; perinuclear region of cytoplasm**		↑
**A0A8I3MIA3**	**EvC ciliary complex subunit 2**	**EVC2**	**Smoothened signaling pathway**	**--**	**Ciliary membrane; cytoplasm; cytoskeleton; cytoskeleton**		↑

**A0A8I3P7W8**	**Gamma-secretase activating protein**	**GSAP**	**--**	**--**	**--**		↑
**A0A8I3PAT3**	**Glutathione peroxidase**	**GPX5**	**Response to oxidative stress**	**Glutathione peroxidase activity**	**--**		↑
**A0A8I3MXM3**	**GMP reductase**	**GMPR2**	**purine nucleobase metabolic process; purine nucleotide metabolic process**	**GMP reductase activity; metal ion binding**	**GMP reductase complex**		↑
**A0A8I3N371**	**Histone H2B**	**H2BC26**	**--**	**DNA binding; protein heterodimerization activity; structural constituent of chromatin**	**Nucleosome; nucleus**		↑
**A0A8I3NXI1**	**Histone H4**	**NA**	**--**	**DNA binding; protein heterodimerization activity;**	**Nucleoplasm; nucleosome**		↑

				structural constituent of chromatin			
A0A1R3UGQ4	Kallikrein A1 - Arginine esterase	KLNA1	Proteolysis	Serine-type endopeptidase activity	--		↑
A0A8I3NL87	Keratin 10	KRT10	Keratinocyte differentiation; peptide cross-linking; protein heterotetramerization	Protein heterodimerization activity; structural constituent of skin epidermis	Cornified envelope; cytoplasm; keratin filament	↑	
A0A8I3PMV1	Keratin 74	KRT74	Intermediate filament cytoskeleton organization	Keratin filament binding	Cytoplasm; keratin filament		↑
A0A8I3PK78	Keratin 75	KRT6A	Hematopoietic progenitor cell differentiation	--	Cornified envelope; keratin filament	↑	

**Q6EIY9**	**Keratin, type II cytoskeletal 1**	**KRT1**	**Protein heterodimerization activity; structural constituent of skin epidermis**	**Keratinization; protein heterotetramerization**	**Cytoplasm; keratin filament; plasma membrane**	↑	
**Q6EIZ1**	**Keratin, type II cytoskeletal 2 epidermal**	**KRT2**	**Keratinization; positive regulation of epidermis development**	**Structural constituent of skin epidermis**	**Cytoplasm; keratin filament**	↑	
**A0A8I3NMR3**	**Lactotransferrin**	**LTF**	**Antibacterial humoral response; disruption by host of symbiont membrane; innate immune response in mucosa; killing of cells of another organism; negative regulation by host of viral process; negative regulation of ATP-dependent activity; negative regulation of cysteine-type**	**Cysteine-type endopeptidase inhibitor activity; heparin binding; iron ion binding; lipopolysaccharide binding; peptidase activity; protein serine/threonine kinase activator activity**	**Cell surface; extracellular space; protein- containing complex; specific granule**	↑	

			**endopeptidase activity; negative regulation of lipopolysaccharide-mediated signaling pathway; negative regulation of single-species biofilm formation in or on host organism; negative regulation of viral genome replication; positive regulation of chondrocyte proliferation; positive regulation of I- kappaB kinase/NF-kappaB signaling; positive regulation of NF-kappaB transcription factor activity; positive regulation of osteoblast differentiation;**				

			**positive regulation of osteoblast proliferation; positive regulation of protein serine/threonine kinase activity; positive regulation of toll-like receptor 4 signaling pathway; regulation of tumor necrosis factor production antifungal humoral response; antimicrobial humoral immune response mediated by antimicrobial peptide; bone morphogenesis; defense response to Gram- negative bacterium**				

A0A8I3MSE6	Lipocalin/cytosolic fatty-acid binding domain-containing protein	LCNL1	--	Small molecule binding	Extracellular region	↑	
A0A8I3NLX2	Olfactory receptor	LOC119880303	Putative odorant or sperm cell receptor	Olfactory receptor activity	Plasma membrane		↑
A0A8I3NE98	Prickle planar cell polarity protein 2	PRICKLE2	--	Zinc ion binding	Cytoplasm		↑
A0A8I3P4J7	Prolactin-induced protein	PIP	--	--	Extracellular region		↑

**Q9XS65**	**Prostaglandin-H2 D-isomerase**	**PTGDS**	**Mast cell degranulation; prostaglandin biosynthetic process; regulation of circadian sleep/wake cycle, sleep**	**Prostaglandin-D synthase activity; retinoid binding; small molecule binding**	**Extracellular region; Golgi apparatus; nuclear membrane; perinuclear region of cytoplasm; rough endoplasmic reticulum; extracellular space**	↑	
**C0LQL0**	**Protein S100**	**S100A8**	**--**	**Calcium ion binding**	**--**		↑
**A0A8I3PNZ6**	**Protein Wnt**	**WNT7A**	**Cell differentiation; system development; tissue development; Wnt signaling pathway**	**Signaling receptor binding**	**Extracellular region**	↑	
**E2RG75**	**Ribonuclease A K1**	**RAK1**	**Defense response to Gram-positive bacterium**	**Nucleic acid binding**	**Extracellular region**	↑	

**A0A8I3N3X3**	**RUN and FYVE domain containing 1**	**RUFY1**	**Protein transport; regulation of endocytosis; small GTPase mediated signal transduction**	**Metal ion binding; SH2 domain binding; SH3 domain binding**	**Cytosol; endosome; nuclear speck**	↑	
**A0A8I3S319**	**Strawberry notch homolog 1**	**SBNO1**	**Regulation of DNA-templated transcription**	**--**	**--**		↑
**A0A8I3NE82**	**Succinate--CoA ligase [GDP-forming] subunit beta, mitochondrial**	**SUCLG2**	**Succinyl-CoA metabolic process; tricarboxylic acid cycle**	**ATP binding; GTP binding; magnesium ion binding; succinate-CoA ligase (ADP-forming) activity; succinate-CoA ligase (GDP-forming) activity**	**Mitochondrion**	↑	
**A0A8I3PPJ8**	**Tetratricopeptide repeat**	**TRANK1**	**--**	**--**	**--**	↑	

	**and ankyrin repeat containing 1**						
**A0A8I3Q4H4**	**TIMP metallopeptidase inhibitor 1**	**TIMP1**	**Cellular response to UV-A; connective tissue replacement involved in inflammatory response wound healing; negative regulation of endopeptidase activity; negative regulation of membrane protein ectodomain proteolysis; negative regulation of metallopeptidase activity; negative regulation of trophoblast cell migration; positive regulation of cell**	**Cytokine activity; metalloendopeptidase Inhibitor activity; zinc ion binding**	**Basement membrane; extracellular space**	↑	

			**population proliferation; regulation of integrin- mediated signaling pathway**				
**A0A8I3P0A5**	**Trypsin**	**LOC475521**	**Proteolysis**	**Serine-type endopeptidase activity**	**-**		↑
**A0A8I3MUN4**	**ZFP28 zinc finger protein**	**ZFP28**	**Regulation of DNA-templated transcription**	**Metal ion binding**	**--**	↑	

https://www.uniprot.org/; Taxonomy:** ***Canis lupus familiaris
