# Supplementary material for: Seasonal Changes in the Seminal Plasma Proteome of the Crab-Eating Fox (Cerdocyon thous)
Source: J Proteome Res. 2025 Dec 31;25(2):723–34. doi: 10.1021/acs.jproteome.5c00694 (PMC12888006; doi:10.1021/acs.jproteome.5c00694)
Supplement: Supplementary file 2 [file pr5c00694_si_002.pdf]

Table S2. Genes involved in gene ontology (molecular function) enrichment in non-reproductive season.

**Pathway**	**Gene**
GMP reductase activity	GMPR; GMPR2
Oxidoreductase activity, acting on NAD(P)H, nitrogenous group as acceptor	GMPR; GMPR2
Metalloendopeptidase inhibitor activity	FETUB; TIMP1
Endopeptidase regulator activity	PSME1; FETUB; LTF; TIMP1
Peptidase regulator activity	PSME1; FETUB; LTF; TIMP1
Endopeptidase activity	KLK2; KLK1; PIP; MMP9
Peptidase activity	KLK2; KLK1; PIP; MMP9; LTF
