# Supplementary material for: Seasonal Changes in the Seminal Plasma Proteome of the Crab-Eating Fox (Cerdocyon thous)
Source: J Proteome Res. 2025 Dec 31;25(2):723–34. doi: 10.1021/acs.jproteome.5c00694 (PMC12888006; doi:10.1021/acs.jproteome.5c00694)
Supplement: Supplementary file 3 [file pr5c00694_si_003.pdf]

**Table S3**. Genes involved in gene ontology (molecular function) enrichment in reproductive season.

| Pathway | Gene |
|---|---|
| Carbohydrate catabolic process | NAGA; PGAM2; GPI; PI4K2A; TPI1; PYGL; PGD; PGK1; NCOR1; ENO1; PGK2 |
| Carbohydrate metabolic process | NAGA; PDGFB; SPAM1; PGAM2; MDH1; PLEK; GPI; HEXB; PI4K2A; LDHC; FUCA1; TPI1; PYGL; ELL; PGD; PGK1; MPI; NCOR1; IMPA2; ENO1; PGK2 |
| Purine nucleoside diphosphate metabolic process | PGAM2; NUDT16; GPI; PI4K2A; TPI1; PGK1; NCOR1; ENO1; PGK2 |
| Purine ribonucleoside diphosphate metabolic process | PGAM2; NUDT16; GPI; PI4K2A; TPI1; PGK1; NCOR1; ENO1; PGK2 |
| Ribonucleoside diphosphate metabolic process | PGAM2; NUDT16; GPI; PI4K2A; TPI1; PGK1; NCOR1; ENO1; PGK2 |
| Pyruvate metabolic process | PGAM2; GPI; PI4K2A; LDHC; TPI1; PGK1; NCOR1; ENO1; PGK2 |
| Glycolytic process | PGAM2; GPI; PI4K2A; TPI1; PGK1; NCOR1; ENO1; PGK2 |
| ATP generation from ADP | PGAM2; GPI; PI4K2A; TPI1; PGK1; NCOR1; ENO1; PGK2 |
| Catabolic process | TBK1; EHMT2; NAGA; PRUNE2; SPAM1; VCP; PGAM2; PABPC4; PSMB2; NCCRP1; RNF213; NUDT16; FLT3; PRICKLE2; YTHDF3; GPI; HSP90B1; USP47; CTSB; CLU; PI4K2A; JMJD7; MMP9; CUL3; GIGYF2; FUCA1; DEPDC5; APOA1; TPI1; PYGL; DDI1; HDC; PSMA3; UBR4; ASRGL1; PSMA4; PGD; PGK1; MPO; NCOR1; ULK2; IMPA2; ENO1; PSMA5; HSPA5; RPS27A; HSP90AB1; PGK2 |
| Multi-organism reproductive process | ACR; ARSA; HSPA1L; EHMT2; SPAM1; ZPBP; NLRP14; YTHDF3; CTSB; CCT8; SOD1; PABPC1L; MMP9; KALRN; ARHGDIB; PRDX4; ACRBP; KRT9; HSPA2; MED1; SPESP1; SPACA3; SPEF2; |
| Multi-organism process | ACR; ARSA; HSPA1L; EHMT2; SPAM1; ZPBP; NLRP14; YTHDF3; CTSB; CCT8; SOD1; PABPC1L; MMP9; KALRN; ARHGDIB; PRDX4; ACRBP; KRT9; HSPA2; MED1; SPESP1; SPACA3; SPEF2; |
| ADP metabolic process | PGAM2; GPI; PI4K2A; TPI1; PGK1; NCOR1; ENO1; PGK2 |
| Reproduction | ACR; ARSA; HSPA1L; EHMT2; SPAM1; ANXA1; ZPBP; NLRP14; YTHDF3; IL1A; CTSB; ROBO2; CCT8; SOD1; LDHC; PABPC1L; MMP9; KALRN; ARHGDIB; PRDX4; ACRBP; ALPL; KRT9; HSPA2; |

| | |
|---|---|
| | MED1; SPESP1; SPACA3; SPEF2; HSP90AB1 |
| Reproductive process | ACR; ARSA; HSPA1L; EHMT2; SPAM1; ANXA1; ZPBP; NLRP14; YTHDF3; IL1A; CTSB; ROBO2; CCT8; SOD1; LDHC; PABPC1L; MMP9; KALRN; ARHGDIB; PRDX4; ACRBP; ALPL; KRT9; HSPA2; MED1; SPESP1; SPACA3; SPEF2; HSP90AB1 |
| Nucleoside diphosphate metabolic process | PGAM2; NUDT16; GPI; PI4K2A; TPI1; PGK1; NCOR1; ENO1; PGK2 |
| Ribose phosphate metabolic process | VCP; PGAM2; NUDT16; GPI; TKT; PI4K2A; LDHC; GMPR2; ATIC; TPI1; EPHA2; PGK1; NCOR1; ENO1; DHODH; PGK2 |
| Actin filament bundle assembly | PLEK; BRAF; LCP1; ACTG1; HSP90B1; CUL3; APOA1; PFN1; ACTN1; PLS3 |
| Organic substance catabolic process | NAGA; SPAM1; VCP; PGAM2; PABPC4; PSMB2; NCCRP1; RNF213; NUDT16; FLT3; PRICKLE2; YTHDF3; GPI; HSP90B1; USP47; CTSB; CLU; PI4K2A; JMJD7; CUL3; GIGYF2; FUCA1; APOA1; TPI1; PYGL; DDI1; HDC; PSMA3; UBR4; ASRGL1; PSMA4; PGD; PGK1; NCOR1; IMPA2; ENO1; PSMA5; HSPA5; RPS27A; HSP90AB1; PGK2 |
| Nucleoside diphosphate phosphorylation | PGAM2; GPI; PI4K2A; TPI1; PGK1; NCOR1; ENO1; PGK2 |
| Nucleotide phosphorylation | PGAM2; GPI; PI4K2A; TPI1; PGK1; NCOR1; ENO1; PGK2 |
| Actin filament bundle organization | PLEK; BRAF; LCP1; ACTG1; HSP90B1; CUL3; APOA1; PFN1; ACTN1; PLS3 |
| Proteolysis | SERPINB8; ACR; VCP; TFPI2; KLK2; KLK1; PSMB2; PIP; GSN; NAALAD2; GSAP; SERPINH1; NCCRP1; RNF213; PRICKLE2; HSP90B1; USP47; CTSB; CLU; SERPINB1; JMJD7; MMP9; CUL3; CSTB; CTSF; LTF; DDI1; TIMP1; PSMA3; UBR4; PSMA4; PGK1; PSMA5; HSPA5; RPS27A; HSP90AB1 |
| Purine-containing compound metabolic process | VCP; PGAM2; NUDT16; FLT3; GPI; PI4K2A; LDHC; GMPR; GMPR2; ATIC; TPI1; EPHA2; PGK1; NCOR1; ENO1; PGK2 |
| Fertilization | ACR; ARSA; HSPA1L; EHMT2; SPAM1; ZPBP; CCT8; ACRBP; SPESP1; SPACA3 |
| Small molecule metabolic process | CS; PRUNE2; ANXA1; VCP; PGAM2; MDH1; PLEK; NUDT16; FLT3; GPI; SOD1; PI4K2A; LDHC; GMPR; GMPR2; FUCA1; APOA1; ATIC; TPI1; ELL; HDC; |

| | |
|---|---|
| | ASRGL1; EPHA2; STARD3; PGD; PGK1; MPO; MPI; NCOR1; IMPA2; PTGDS; ENO1; DHODH; PGK2 |
| Sperm-egg recognition | ACR; ARSA; HSPA1L; ZPBP; CCT8; SPACA3 |
| Protein folding | HSPA1L; ST13; HSPA9; HSP90B1; CLU; CCT8; PDIA3; PRDX4; HSPA2; HSP90AA1; HSPA5; HSP90AB1 |
| Ribonucleotide metabolic process | VCP; PGAM2; NUDT16; ; GPI; PI4K2A; LDHC; GMPR2; ATIC; TPI1; EPHA2; PGK1; NCOR1; ENO1; DHODH; PGK2 |
| Regulation of actin filament-based process | AKAP9; PLEK; CAPZA2; GSN; BRAF; ACTG1; CAPG; CTNNA2; RYR2; ANK2; ARHGDIB; APOA1; CAPZB; PFN1; ARHGEF19 |
| Purine nucleotide metabolic process | VCP; PGAM2; NUDT16; GPI; PI4K2A; LDHC; GMPR; GMPR2; ATIC; TPI1; EPHA2; PGK1; NCOR1; ENO1; PGK2 |
