# Supplementary material for: Seasonal Changes in the Seminal Plasma Proteome of the Crab-Eating Fox (Cerdocyon thous)
Source: J Proteome Res. 2025 Dec 31;25(2):723–34. doi: 10.1021/acs.jproteome.5c00694 (PMC12888006; doi:10.1021/acs.jproteome.5c00694)
Supplement: Supplementary file 4 [file pr5c00694_si_004.pdf]

Table S4. ID mapping and gene ontology of proteins found in the non-reproduction season.

Protein ID UNIPROT	Protein name	Gene	Gene ontology		
			Biological process	Cellular component	Molecular function
A0A8I3RR71	40S ribosomal protein S25	RPS25	Ribosomal small subunit biogenesis [GO:0042274]; rrna processing [GO:0006364]	Cytosolic small ribosomal subunit [GO:0022627]; nucleolus [GO:0005730]; postsynaptic density [GO:0014069]	Structural constituent of ribosome [GO:0003735]
A0A8I3Q739	Actin beta like 2	ACTBL2		Glutamatergic synapse [GO:0098978]	
A0A8I3S044	Actin beta	ACTB			
P49822	Albumin (allergen Can f 3)	ALB	Cellular response to calcium ion starvation [GO:0072732]; cellular response to starvation [GO:0009267]; negative regulation of mitochondrial depolarization [GO:0051902]	Cytoplasm [GO:0005737]; extracellular space [GO:0005615]; protein-containing complex [GO:0032991]	DNA binding [GO:0003677]; enterobactin binding [GO:1903981]; fatty acid binding [GO:0005504]; metal ion binding [GO:0046872]; pyridoxal phosphate binding [GO:0030170]; toxic substance binding [GO:0015643]
A0A8I3MGB5	Alkaline phosphatase (EC 3.1.3.1)	ALPL	Bone mineralization [GO:0030282]; calcium ion homeostasis [GO:0055074]; cellular homeostasis [GO:0019725]; cellular response to organic cyclic compound [GO:0071407]; developmental process involved in reproduction [GO:0003006]; endochondral ossification [GO:0001958]; futile creatine cycle [GO:0140651]; inhibition of non-skeletal tissue mineralization [GO:0140928]; phosphate ion homeostasis [GO:0055062]; positive regulation of cold-induced thermogenesis [GO:0120162]; pyridoxal phosphate metabolic process [GO:0042822]; response to antibiotic [GO:0046677]; response to sodium phosphate [GO:1904383]; response to vitamin B6 [GO:0034516]; response to vitamin D [GO:0033280]	Extracellular matrix [GO:0031012]; extracellular membrane-bounded organelle [GO:0065010]; mitochondrial intermembrane space [GO:0005758]; mitochondrial membrane [GO:0031966]; plasma membrane [GO:0005886]; side of membrane [GO:0098552]	Alkaline phosphatase activity [GO:0004035]; calcium ion binding [GO:0005509]; phosphoamidase activity [GO:0050187]; phosphoethanolamine phosphatase activity [GO:0052732]; pyridoxal phosphatase activity [GO:0033883]; pyrophosphatase activity [GO:0016462]
A0A8I3Q7I5	ATP binding cassette subfamily A member 8	ABCA8	Positive regulation of cholesterol efflux [GO:0010875]; sphingomyelin biosynthetic process [GO:0006686]; xenobiotic transmembrane transport [GO:0006855]	Basolateral plasma membrane [GO:0016323]; endoplasmic reticulum [GO:0005783]	ABC-type xenobiotic transporter activity [GO:0008559]; ATP binding [GO:0005524]; ATP hydrolysis activity [GO:0016887]

A0A8I3N1V3	Atypical kinase COQ8A, mitochondrial (Chaperone activity of bc1 complex-like) (Coenzyme Q protein 8A) (aarF domain-containing protein kinase 3)	COQ8A	Ubiquinone biosynthetic process [GO:0006744]	Membrane [GO:0016020]; mitochondrion [GO:0005739]	Kinase activity [GO:0016301]
A0A8I3NLW4	Catenin alpha-2 (Alpha N-catenin)	CTNNA2	Cell adhesion [GO:0007155]; cell differentiation [GO:0030154]	Actin cytoskeleton [GO:0015629]; adherens junction [GO:0005912]; axon [GO:0030424]; cytoplasm [GO:0005737]; nucleus [GO:0005634]; plasma membrane [GO:0005886]	Actin filament binding [GO:0051015]; cadherin binding [GO:0045296]; structural molecule activity [GO:0005198]
A0A8I3NF26	Clusterin	CLU		Chromaffin granule [GO:0042583]; cytosol [GO:0005829]; endoplasmic reticulum [GO:0005783]; extracellular region [GO:0005576]; mitochondrial membrane [GO:0031966]; nucleus [GO:0005634]; perinuclear region of cytoplasm [GO:0048471]	
A0A8I3PN88	DDB1 and CUL4 associated factor 15	DCAF15	Protein polyubiquitination [GO:0000209]; regulation of natural killer cell activation [GO:0032814]	Cul4-RING E3 ubiquitin ligase complex [GO:0080008]	Small molecule binding [GO:0036094]
A0A8I3MIA3	EvC ciliary complex subunit 2	EVC2	Smoothened signaling pathway [GO:0007224]	Ciliary membrane [GO:0060170]; cytoplasm [GO:0005737]; cytoskeleton [GO:0005856]	
A0A8I3P629	Fetuin B	FETUB			Cysteine-type endopeptidase inhibitor activity [GO:0004869]
A0A1K0GGH0	Globin A2 (Globin A3) (Hemoglobin subunit beta/delta hybrid)	GLNA2 GLNA3 LOC609402		Hemoglobin complex [GO:0005833]	Heme binding [GO:0020037]; metal ion binding [GO:0046872]; oxygen binding [GO:0019825]; oxygen carrier activity [GO:0005344]
A0A8I3MXM3	GMP reductase (GMPR) (EC 1.7.1.7) (Guanosine 5'-monophosphate oxidoreductase)	GMPR2 GMPR	Purine nucleobase metabolic process [GO:0006144]; purine nucleotide metabolic process [GO:0006163]	GMP reductase complex [GO:1902560]	GMP reductase activity [GO:0003920]; metal ion binding [GO:0046872]

	(Guanosine monophosphate reductase)				
A0A8I3N2P1	Heat shock transcription factor 1	HSF1		Nucleus [GO:0005634]	DNA-binding transcription factor activity [GO:0003700]; sequence-specific DNA binding [GO:0043565]
A0A8I3P6S7	Ig-like domain-containing protein				
E2RG75	Inactive ribonuclease-like protein 9	RAK1 RNASE9	Defense response to Gram-positive bacterium [GO:0050830]	Extracellular region [GO:0005576]	Nucleic acid binding [GO:0003676]
A0A8I3NVH3	Integrin subunit alpha V	ITGAV	Cell adhesion [GO:0007155]; integrin-mediated signaling pathway [GO:0007229]	Integrin complex [GO:0008305]	
A0A8I3NM17	Kallikrein 1	KLK1	Proteolysis [GO:0006508]		Serine-type endopeptidase activity [GO:0004252]
A0A1R3UGQ4	Kallikrein A1 (Kallikrein-related peptidase 2)	KLNA1 KLK2	Proteolysis [GO:0006508]		Serine-type endopeptidase activity [GO:0004252]
A0A8I3PJ84	Keratin 18	KRT18		Intermediate filament [GO:0005882]	Structural molecule activity [GO:0005198]
A0A8I3PK78	Keratin 75	KRT75	Hematopoietic progenitor cell differentiation [GO:0002244]	Cornified envelope [GO:0001533]; keratin filament [GO:0045095]	
A0A8I3NL87	Keratin, type I cytoskeletal 10 (Cytokeratin-10) (Keratin-10)	KRT10	Keratinocyte differentiation [GO:0030216]; protein heterotetramerization [GO:0051290]	Cell surface [GO:0009986]; cornified envelope [GO:0001533]; cytoplasm [GO:0005737]; extracellular region [GO:0005576]; keratin filament [GO:0045095]	Protein heterodimerization activity [GO:0046982]; structural constituent of skin epidermis [GO:0030280]
A0A8I3NNW0	Keratin 9	KRT9	Intermediate filament organization [GO:0045109]; skin development [GO:0043588]; spermatogenesis [GO:0007283]	Intermediate filament [GO:0005882]	Structural molecule activity [GO:0005198]
Q6EIY9	Keratin, type II cytoskeletal 1 (Cytokeratin-1) (CK-1) (Epithelial keratin-1) (Keratin-1) (K1) (Type-II keratin Kb1)	KRT1 KER1	Intermediate filament organization [GO:0045109]; keratinization [GO:0031424]; protein heterotetramerization [GO:0051290]	Cytoplasm [GO:0005737]; keratin filament [GO:0045095]; plasma membrane [GO:0005886]	Protein heterodimerization activity [GO:0046982]; structural constituent of skin epidermis [GO:0030280]
Q6EIZ1	Keratin, type II cytoskeletal 2	KRT2 K2E KRT2A	Intermediate filament organization [GO:0045109]; keratinization [GO:0031424];	Cytoplasm [GO:0005737]; keratin filament [GO:0045095]	Structural constituent of skin epidermis [GO:0030280]

	epidermal (Cytokeratin-2e) (CK-2e) (Epithelial keratin-2e) (Keratin-2 epidermis) (Keratin-2e) (K2e) (Type-II keratin Kb2)		positive regulation of epidermis development [GO:0045684]		
A0A8I3NMR3	Lactotransferrin	LTF	Antibacterial humoral response [GO:0019731]; antifungal humoral response [GO:0019732]; antimicrobial humoral immune response mediated by antimicrobial peptide [GO:0061844]; bone morphogenesis [GO:0060349]; defense response to Gram-negative bacterium [GO:0050829]; innate immune response in mucosa [GO:0002227]; iron ion transport [GO:0006826]; killing of cells of another organism [GO:0031640]; negative regulation by host of viral process [GO:0044793]; negative regulation of lipopolysaccharide-mediated signaling pathway [GO:0031665]; negative regulation of single-species biofilm formation in or on host organism [GO:1900229]; negative regulation of viral genome replication [GO:0045071]; ossification [GO:0001503]; positive regulation of canonical NF-kappab signal transduction [GO:0043123]; positive regulation of chondrocyte proliferation [GO:1902732]; positive regulation of osteoblast differentiation [GO:0045669]; positive regulation of osteoblast proliferation [GO:0033690]; positive regulation of toll-like receptor 4 signaling pathway [GO:0034145]; proteolysis [GO:0006508]; regulation of tumor necrosis factor production [GO:0032680]	Cell surface [GO:0009986]; extracellular space [GO:0005615]; protein-containing complex [GO:0032991]; specific granule [GO:0042581]	Cysteine-type endopeptidase inhibitor activity [GO:0004869]; heparin binding [GO:0008201]; iron ion binding [GO:0005506]; lipopolysaccharide binding [GO:0001530]; membrane destabilizing activity [GO:0140912]; protein serine/threonine kinase activator activity [GO:0043539]; serine-type peptidase activity [GO:0008236]
A0A8I3MSE6	Lipocalin like 1	LCNL1			Small molecule binding [GO:0036094]
P81709	Lysozyme C, spleen isozyme (EC		Defense response to Gram-negative bacterium [GO:0050829]; defense response to Gram-		Lysozyme activity [GO:0003796]

	3.2.1.17) (1,4-beta-N-acetylmuramidase C)		positive bacterium [GO:0050830]; killing of cells of another organism [GO:0031640]		
A0A8I3P478	Matrix metalloproteinase-9 (EC 3.4.24.35) (92 kDa gelatinase) (92 kDa type IV collagenase) (Gelatinase B)	MMP9	Apoptotic process [GO:0006915]; cell migration [GO:0016477]; cellular response to UV-A [GO:0071492]; collagen catabolic process [GO:0030574]; embryo implantation [GO:0007566]; endodermal cell differentiation [GO:0035987]; extracellular matrix organization [GO:0030198]; negative regulation of epithelial cell differentiation involved in kidney development [GO:2000697]; negative regulation of intrinsic apoptotic signaling pathway [GO:2001243]; positive regulation of apoptotic process [GO:0043065]; positive regulation of epidermal growth factor receptor signaling pathway [GO:0045742]; positive regulation of keratinocyte migration [GO:0051549]; positive regulation of release of cytochrome c from mitochondria [GO:0090200]; positive regulation of vascular associated smooth muscle cell proliferation [GO:1904707]; proteolysis [GO:0006508]; response to amyloid-beta [GO:1904645]; skeletal system development [GO:0001501]	Extracellular matrix [GO:0031012]; extracellular space [GO:0005615]	Identical protein binding [GO:0042802]; metalloendopeptidase activity [GO:0004222]; zinc ion binding [GO:0008270]
A0A8I3Q4H4	Metalloproteinase inhibitor 1 (Tissue inhibitor of metalloproteinases 1)	TIMP1	Cellular response to UV-A [GO:0071492]; connective tissue replacement involved in inflammatory response wound healing [GO:0002248]; negative regulation of membrane protein ectodomain proteolysis [GO:0051045]; negative regulation of trophoblast cell migration [GO:1901164]; positive regulation of cell population proliferation [GO:0008284]; regulation of integrin-mediated signaling pathway [GO:2001044]	Basement membrane [GO:0005604]; extracellular space [GO:0005615]	Cytokine activity [GO:0005125]; growth factor activity [GO:0008083]; metalloendopeptidase inhibitor activity [GO:0008191]; zinc ion binding [GO:0008270]
A0A8I3P0A5	trypsin (EC 3.4.21.4)	LOC475521	Proteolysis [GO:0006508]	Extracellular region [GO:0005576]	Serine-type endopeptidase activity [GO:0004252]

A0A8I3MWC0	PR domain zinc finger protein 1 (EC 2.1.1.-)	PRDM1	Aorta development [GO:0035904]; artery morphogenesis [GO:0048844]; cell fate commitment [GO:0045165]; coronary vasculature development [GO:0060976]; eye photoreceptor cell development [GO:0042462]; gene expression [GO:0010467]; germ cell development [GO:0007281]; heart valve development [GO:0003170]; intestinal epithelial cell development [GO:0060576]; kidney development [GO:0001822]; maternal placenta development [GO:0001893]; morphogenesis of a branching structure [GO:0001763]; negative regulation of gene expression [GO:0010629]; positive regulation of gene expression [GO:0010628]; post-embryonic development [GO:0009791]; regulation of cell population proliferation [GO:0042127]; regulation of extrathymic T cell differentiation [GO:0033082]; regulation of natural killer cell differentiation [GO:0032823]; regulation of NK T cell differentiation [GO:0051136]; retinal bipolar neuron differentiation [GO:0060040]; sebum secreting cell proliferation [GO:1990654]; trophoblast giant cell differentiation [GO:0060707]; ventricular septum development [GO:0003281]	Cytoplasm [GO:0005737]; nucleolus [GO:0005730]; nucleoplasm [GO:0005654]	DNA-binding transcription repressor activity, RNA polymerase II-specific [GO:0001227]; histone methyltransferase binding [GO:1990226]; metal ion binding [GO:0046872]; promoter-specific chromatin binding [GO:1990841]; RNA polymerase II cis-regulatory region sequence-specific DNA binding [GO:0000978]; transferase activity [GO:0016740]
A0A8I3NE98	Prickle planar cell polarity protein 2	PRICKLE2		Cytoplasm [GO:0005737]	Zinc ion binding [GO:0008270]
A0A8I3P4J7	Prolactin-induced protein	PIP		Extracellular region [GO:0005576]	
Q9XS65	Prostaglandin-H2 D-isomerase (EC 5.3.99.2) (Glutathione-independent PGD synthase) (Lipocalin-type prostaglandin-D synthase) (Prostaglandin-D2	PTGDS	Mast cell degranulation [GO:0043303]; prostaglandin biosynthetic process [GO:0001516]; regulation of circadian sleep/wake cycle, sleep [GO:0045187]	Extracellular region [GO:0005576]; extracellular space [GO:0005615]; Golgi apparatus [GO:0005794]; nuclear membrane [GO:0031965]; perinuclear region of cytoplasm [GO:0048471]; rough endoplasmic reticulum [GO:0005791]	Prostaglandin-D synthase activity [GO:0004667]; retinoid binding [GO:0005501]; small molecule binding [GO:0036094]

	synthase) (PGD2 synthase) (PGDS) (PGDS2)				
A0A8I3PNZ6	Protein Wnt	WNT7A	Animal organ development [GO:0048513]; cell differentiation [GO:0030154]; system development [GO:0048731]; tissue development [GO:0009888]; Wnt signaling pathway [GO:0016055]	Extracellular region [GO:0005576]	Signaling receptor binding [GO:0005102]
A0A8I3MW08	Protocadherin 7	PCDH7	Homophilic cell adhesion via plasma membrane adhesion molecules [GO:0007156]	Plasma membrane [GO:0005886]	Calcium ion binding [GO:0005509]
A0A8I3Q8R9	Uncharacterized protein	LOC487476	Signal transduction [GO:0007165]		
A0A8I3N3X3	RUN and FYVE domain containing 1	RUFY1	Early endosome to Golgi transport [GO:0034498]; endosomal vesicle fusion [GO:0034058]; protein transport [GO:0015031]; regulation of endocytosis [GO:0030100]; small gtpase-mediated signal transduction [GO:0007264]	Cytosol [GO:0005829]; early endosome membrane [GO:0031901]; nuclear speck [GO:0016607]	Metal ion binding [GO:0046872]; protein-macromolecule adaptor activity [GO:0030674]; SH2 domain binding [GO:0042169]; SH3 domain binding [GO:0017124]
A0A8I3MT21	Ryanodine receptor 2	RYR2	Calcium ion transport into cytosol [GO:0060402]; cardiac muscle contraction [GO:0060048]; cellular response to caffeine [GO:0071313]; detection of calcium ion [GO:0005513]; establishment of protein localization to endoplasmic reticulum [GO:0072599]; intracellular calcium ion homeostasis [GO:0006874]; positive regulation of sequestering of calcium ion [GO:0051284]; positive regulation of the force of heart contraction [GO:0098735]; regulation of atrial cardiac muscle cell action potential [GO:0098910]; regulation of AV node cell action potential [GO:0098904]; regulation of cardiac muscle contraction by calcium ion signaling [GO:0010882]; regulation of heart rate [GO:0002027]; regulation of SA node cell action potential [GO:0098907]; regulation of ventricular cardiac muscle cell action potential [GO:0098911]; release of sequestered calcium ion into cytosol by sarcoplasmic reticulum	Calcium channel complex [GO:0034704]; sarcoplasmic reticulum membrane [GO:0033017]; Z disc [GO:0030018]	Calcium ion binding [GO:0005509]; calmodulin binding [GO:0005516]; identical protein binding [GO:0042802]; protein kinase A catalytic subunit binding [GO:0034236]; protein kinase A regulatory subunit binding [GO:0034237]; ryanodine-sensitive calcium-release channel activity [GO:0005219]; suramin binding [GO:0043924]

			[GO:0014808]; response to muscle activity [GO:0014850]; response to muscle stretch [GO:0035994]; response to redox state [GO:0051775]; type B pancreatic cell apoptotic process [GO:0097050]		
A0A8I3NE82	Succinate--CoA ligase [GDP-forming] subunit beta, mitochondrial (EC 6.2.1.4) (GTP-specific succinyl-CoA synthetase subunit beta) (G-SCS) (GTPSCS) (Succinyl-CoA synthetase beta-G chain) (SCS-betaG)	SUCLG2	Succinyl-coa metabolic process [GO:0006104]; tricarboxylic acid cycle [GO:0006099]	Mitochondrion [GO:0005739]	ATP binding [GO:0005524]; GTP binding [GO:0005525]; magnesium ion binding [GO:0000287]; succinate-coa ligase (ADP-forming) activity [GO:0004775]; succinate-coa ligase (GDP-forming) activity [GO:0004776]
A0A8I3PPJ8	Tetratricopeptide repeat and ankyrin repeat containing 1	TRANK1			
A0A8I3NWM9	Transforming growth factor beta receptor 3	TGFBR3		Extracellular region [GO:0005576]; plasma membrane [GO:0005886]	
A0A8I3N8D4	Transmembrane protein 63A	TMEM63A		Endomembrane system [GO:0012505]; membrane [GO:0016020]	Calcium-activated cation channel activity [GO:0005227]; nucleic acid binding [GO:0003676]
A0A8I3MVM8	Treacle ribosome biosis factor 1	TCOF1			
A0A8I3MU16	Proteasome activator complex subunit 1 (Proteasome activator 28 subunit alpha)	PSME1		Proteasome activator complex [GO:0008537]	
A0A8I3NX52	Uncharacterized protein				
A0A8I3PJ06	YEATS domain containing 2	YEATS2	Regulation of DNA-templated transcription [GO:0006355]	Nucleus [GO:0005634]	

A0A8I3MUN4	ZFP28 zinc finger protein	ZFP28	Regulation of DNA-templated transcription [GO:0006355]	DNA binding [GO:0003677]; metal ion binding [GO:0046872]
