# Supplementary material for: Seasonal Changes in the Seminal Plasma Proteome of the Crab-Eating Fox (Cerdocyon thous)
Source: J Proteome Res. 2025 Dec 31;25(2):723–34. doi: 10.1021/acs.jproteome.5c00694 (PMC12888006; doi:10.1021/acs.jproteome.5c00694)
Supplement: Supplementary file 5 [file pr5c00694_si_005.pdf]

Table S5. ID mapping and gene ontology of proteins found in the reproductive season.

Protein ID UNIPROT	Protein name	Gene	Gene ontology		
			Biological process	Cellular component	Molecular function
A0A8I3MYN5	[histone H4]-N-methyl-L-lysine20 N-methyltransferase KMT5B (EC 2.1.1.361) (EC 2.1.1.362) ([histone H4]-lysine20 N-methyltransferase KMT5B)	KMT5B	Methylation [GO:0032259]; muscle organ development [GO:0007517]	Chromosome [GO:0005694]; nucleus [GO:0005634]	Histone H4K20 methyltransferase activity [GO:0042799]
A0A8I3NCX8	14-3-3 domain-containing protein	LOC100855903		Cytoplasm [GO:0005737]	
A0A8I3Q1P7	Tyrosine 3-monooxygenase/tryptophan 5-monooxygenase activation protein zeta	YWHAZ		Cytoplasm [GO:0005737]	
A0A8I3NXE5	Thioredoxin-dependent peroxiredoxin (EC 1.11.1.24)				Peroxiredoxin activity [GO:0051920]
A0A8I3MUG5	Small ribosomal subunit protein es1	RPS3A	Cell differentiation [GO:0030154]; translation [GO:0006412]	Cytosolic small ribosomal subunit [GO:0022627]; nucleolus [GO:0005730]	Structural constituent of ribosome [GO:0003735]
A0A8I3MQ54	6-phosphogluconate dehydrogenase, decarboxylating (EC 1.1.1.44)	PGD	D-gluconate metabolic process [GO:0019521]; pentose-phosphate shunt [GO:0006098]		NADP binding [GO:0050661]; phosphogluconate dehydrogenase (decarboxylating) activity [GO:0004616]
A0A8I3NCJ5	78 kda glucose-regulated protein (Binding-immunoglobulin protein) (Heat shock protein 70 family protein 5) (Heat	HSPA5	Cellular response to glucose starvation [GO:0042149]; cellular response to interleukin-4 [GO:0071353]; cerebellar Purkinje cell layer development [GO:0021680]; cerebellum structural organization [GO:0021589]; endoplasmic reticulum unfolded protein response [GO:0030968]; ER overload response	Cell surface [GO:0009986]; COP9 signalosome [GO:0008180]; cytosol [GO:0005829]; endoplasmic reticulum chaperone complex [GO:0034663]; endoplasmic reticulum lumen [GO:0005788]; endoplasmic reticulum membrane	ATP binding [GO:0005524]; ATP hydrolysis activity [GO:0016887]; ATP-dependent protein folding chaperone [GO:0140662]; misfolded protein binding [GO:0051787]; protein domain specific binding [GO:0019904]; protein

	shock protein family A member 5) (Immunoglobulin heavy chain-binding protein)		[GO:0006983]; maintenance of protein localization in endoplasmic reticulum [GO:0035437]; negative regulation of apoptotic process [GO:0043066]; negative regulation of PERK-mediated unfolded protein response [GO:1903898]; negative regulation of transforming growth factor beta receptor signaling pathway [GO:0030512]; positive regulation of cell migration [GO:0030335]; positive regulation of protein ubiquitination [GO:0031398]; post-translational protein targeting to membrane, translocation [GO:0031204]; proteolysis involved in protein catabolic process [GO:0051603]	[GO:0005789]; endoplasmic reticulum-Golgi intermediate compartment [GO:0005793]; midbody [GO:0030496]; mitochondrion [GO:0005739]; plasma membrane [GO:0005886]	serine/threonine kinase inhibitor activity [GO:0030291]; ribosome binding [GO:0043022]; ubiquitin protein ligase binding [GO:0031625]
A0A8I3RV44	ABC transporter domain-containing protein	ABCA13		Membrane [GO:0016020]	ABC-type transporter activity [GO:0140359]; ATP binding [GO:0005524]; ATP hydrolysis activity [GO:0016887]
A0A8I3PIE7	Acetylserotonin O-methyltransferase like	ASMTL	Methylation [GO:0032259]		Nucleoside triphosphate diphosphatase activity [GO:0047429]; O-methyltransferase activity [GO:0008171]
A0A8I3PQV8	Lysosomal acid phosphatase (EC 3.1.3.2)	ACP2		Lysosomal lumen [GO:0043202]; lysosomal membrane [GO:0005765]	
A0A8I3PYH1	Acrosin (EC 3.4.21.10)	ACR	Acrosome reaction [GO:0007340]; binding of sperm to zona pellucida [GO:0007339]; penetration of zona pellucida [GO:0007341]; proteolysis [GO:0006508]	Acrosomal vesicle [GO:0001669]; protein-containing complex [GO:0032991]	D-mannose binding [GO:0005537]; serine-type endopeptidase activity [GO:0004252]
A0A8I3PWI5	Acrosin-binding protein (Acrosin-binding protein, 60 kda form) (Proacrosin-binding protein sp32)	ACRBP	Acrosome assembly [GO:0001675]; fertilization [GO:0009566]	Acrosomal vesicle [GO:0001669]	
A0A8I3NRH5	Acrosomal vesicle protein 1	ACRV1			
A0A8I3MHG9	Actin alpha 1, skeletal muscle	ACTA1		Cytoplasm [GO:0005737]; cytoskeleton [GO:0005856]	
A0A8I3S044	Actin beta	ACTB			

A0A8I3NE73	Actin gamma 1	ACTG1	Angiogenesis [GO:0001525]; cellular response to type II interferon [GO:0071346]; morphogenesis of a polarized epithelium [GO:0001738]; positive regulation of cell migration [GO:0030335]; positive regulation of gene expression [GO:0010628]; positive regulation of wound healing [GO:0090303]; protein localization to bicellular tight junction [GO:1902396]; regulation of focal adhesion assembly [GO:0051893]; regulation of stress fiber assembly [GO:0051492]; regulation of synaptic vesicle endocytosis [GO:1900242]; regulation of transepithelial transport [GO:0150111]; sarcomere organization [GO:0045214]; tight junction assembly [GO:0120192]	Apical junction complex [GO:0043296]; basal body patch [GO:0120220]; calyx of Held [GO:0044305]; extracellular exosome [GO:0070062]; filamentous actin [GO:0031941]; myofibril [GO:0030016]; phagocytic vesicle [GO:0045335]; Schaffer collateral - CA1 synapse [GO:0098685]	Identical protein binding [GO:0042802]; profilin binding [GO:0005522]; structural constituent of cytoskeleton [GO:0005200]; ubiquitin protein ligase binding [GO:0031625]
O18840	Actin, cytoplasmic 1 (EC 3.6.4.-) (Beta-actin) [Cleaved into: Actin, cytoplasmic 1, N-terminally processed]	ACTB	Axonogenesis [GO:0007409]; cell motility [GO:0048870]	Actin cytoskeleton [GO:0015629]; actin filament [GO:0005884]; axon [GO:0030424]; cytoplasm [GO:0005737]; cytoskeleton [GO:0005856]; dense body [GO:0097433]; focal adhesion [GO:0005925]; membrane [GO:0016020]; nua4 histone acetyltransferase complex [GO:0035267]; nucleus [GO:0005634]; plasma membrane [GO:0005886]; protein-containing complex [GO:0032991]; synapse [GO:0045202]	ATP binding [GO:0005524]; hydrolase activity [GO:0016787]; protein kinase binding [GO:0019901]; structural constituent of postsynaptic actin cytoskeleton [GO:0098973]
A0A8I3RPA5	Actin, cytoplasmic 1			Cytoskeleton [GO:0005856]	
A0A8I3MJF7	Actinin alpha 1	ACTN1		Anchoring junction [GO:0070161]; cytoskeleton [GO:0005856]; plasma membrane [GO:0005886]; ruffle [GO:0001726]; Z disc [GO:0030018]	Actin binding [GO:0003779]; calcium ion binding [GO:0005509]
A0A8I3MU01	Acylphosphatase-1 (EC 3.6.1.7) (Acylphosphate				Acylphosphatase activity [GO:0003998]

	phosphohydrolase 1)				
A0A8I3MHD9	ADP ribosylation factor like gtpase 15	ARL15			GTP binding [GO:0005525]; gtpase activity [GO:0003924]
A0A8I3PUZ6	Uncharacterized protein	ARL4A			GTP binding [GO:0005525]; gtpase activity [GO:0003924]
A0A8I3NIN3	A-kinase anchoring protein 9	AKAP9	Cellular response to camp [GO:0071320]; maintenance of centrosome location [GO:0051661]; microtubule nucleation [GO:0007020]; positive regulation of microtubule polymerization [GO:0031116]; protein-containing complex localization [GO:0031503]; regulation of cardiac muscle cell action potential involved in regulation of contraction [GO:0098909]; regulation of Golgi organization [GO:1903358]; regulation of heart rate by cardiac conduction [GO:0086091]; regulation of ventricular cardiac muscle cell membrane repolarization [GO:0060307]; signal transduction [GO:0007165]	Centrosome [GO:0005813]; cis-Golgi network [GO:0005801]; glutamatergic synapse [GO:0098978]; Golgi stack [GO:0005795]; voltage-gated potassium channel complex [GO:0008076]	DNA binding [GO:0003677]; molecular adaptor activity [GO:0060090]; potassium channel regulator activity [GO:0015459]; protein kinase A regulatory subunit binding [GO:0034237]; transmembrane transporter binding [GO:0044325]
P49822	Albumin (allergen Can f 3)	ALB	Cellular response to calcium ion starvation [GO:0072732]; cellular response to starvation [GO:0009267]; negative regulation of mitochondrial depolarization [GO:0051902]	Cytoplasm [GO:0005737]; extracellular space [GO:0005615]; protein-containing complex [GO:0032991]	DNA binding [GO:0003677]; enterobactin binding [GO:1903981]; fatty acid binding [GO:0005504]; metal ion binding [GO:0046872]; pyridoxal phosphate binding [GO:0030170]; toxic substance binding [GO:0015643]
A0A8I3PBM7	Aldo-keto reductase family 1 member A1 (EC 1.1.1.19) (EC 1.1.1.2) (EC 1.1.1.20) (EC 1.1.1.372) (EC 1.1.1.54) (Alcohol dehydrogenase [NADP(+)]) (Aldehyde reductase) (Glucuronate reductase)	AKR1A1	Aldehyde catabolic process [GO:0046185]	Apical plasma membrane [GO:0016324]; cytosol [GO:0005829]	Alcohol dehydrogenase (NADP+) activity [GO:0008106]

	(Glucuronolactone reductase) (S-nitroso-coa reductase)				
A0A8I3MGX2	Aldo-keto reductase family 1 member E2	AKR1E2			Oxidoreductase activity [GO:0016491]
A0A8I3MGB5	Alkaline phosphatase (EC 3.1.3.1)	ALPL	Bone mineralization [GO:0030282]; calcium ion homeostasis [GO:0055074]; cellular homeostasis [GO:0019725]; cellular response to organic cyclic compound [GO:0071407]; developmental process involved in reproduction [GO:0003006]; endochondral ossification [GO:0001958]; futile creatine cycle [GO:0140651]; inhibition of non-skeletal tissue mineralization [GO:0140928]; phosphate ion homeostasis [GO:0055062]; positive regulation of cold-induced thermogenesis [GO:0120162]; pyridoxal phosphate metabolic process [GO:0042822]; response to antibiotic [GO:0046677]; response to sodium phosphate [GO:1904383]; response to vitamin B6 [GO:0034516]; response to vitamin D [GO:0033280]	Extracellular matrix [GO:0031012]; extracellular membrane-bounded organelle [GO:0065010]; mitochondrial intermembrane space [GO:0005758]; mitochondrial membrane [GO:0031966]; plasma membrane [GO:0005886]; side of membrane [GO:0098552]	Alkaline phosphatase activity [GO:0004035]; calcium ion binding [GO:0005509]; phosphoamidase activity [GO:0050187]; phosphoethanolamine phosphatase activity [GO:0052732]; pyridoxal phosphatase activity [GO:0033883]; pyrophosphatase activity [GO:0016462]
A0A8I3MLJ9	Alpha-1,4 glucan phosphorylase (EC 2.4.1.1)	PYGL	Glycogen metabolic process [GO:0005977]	Cytosol [GO:0005829]	Glycogen phosphorylase activity [GO:0008184]; nucleotide binding [GO:0000166]; pyridoxal phosphate binding [GO:0030170]
A0A8I3NBJ3	Alpha-1-acid glycoprotein	LOC100685620	Acute-phase response [GO:0006953]; regulation of immune system process [GO:0002682]	Extracellular space [GO:0005615]	
A0A8I3NKJ0	Alpha-galactosidase (EC 3.2.1.-)	NAGA	Carbohydrate catabolic process [GO:0016052]; glycolipid catabolic process [GO:0019377]	Lysosome [GO:0005764]; membrane [GO:0016020]	Alpha-N-acetylgalactosaminidase activity [GO:0008456]; protein homodimerization activity [GO:0042803]
A0A8I3NUL2	Alpha-L-fucosidase (EC 3.2.1.51)	FUCA1	Fucose metabolic process [GO:0006004]; lipid metabolic process [GO:0006629]	Lysosome [GO:0005764]	Alpha-L-fucosidase activity [GO:0004560]
A0A8I3PT63	Ankyrin 2	ANK2	Signal transduction [GO:0007165]	Cytoskeleton [GO:0005856]; membrane [GO:0016020]	
A0A8I3M9P4	Annexin	ANXA1	Actin cytoskeleton organization [GO:0030036]; alpha-beta T cell differentiation [GO:0046632];	Actin filament [GO:0005884]; apical plasma membrane [GO:0016324];	Calcium ion binding [GO:0005509]; calcium-dependent phospholipid

			arachidonate secretion [GO:0050482]; cellular response to glucocorticoid stimulus [GO:0071385]; cellular response to vascular endothelial growth factor stimulus [GO:0035924]; G protein-coupled receptor signaling pathway, coupled to cyclic nucleotide second messenger [GO:0007187]; granulocyte chemotaxis [GO:0071621]; inflammatory response [GO:0006954]; keratinocyte differentiation [GO:0030216]; monocyte chemotaxis [GO:0002548]; myoblast migration involved in skeletal muscle regeneration [GO:0014839]; negative regulation of exocytosis [GO:0045920]; negative regulation of interleukin-8 production [GO:0032717]; negative regulation of T-helper 2 cell differentiation [GO:0045629]; neutrophil activation [GO:0042119]; neutrophil clearance [GO:0097350]; positive regulation of cell migration involved in sprouting angiogenesis [GO:0090050]; positive regulation of G1/S transition of mitotic cell cycle [GO:1900087]; positive regulation of interleukin-2 production [GO:0032743]; positive regulation of neutrophil apoptotic process [GO:0033031]; positive regulation of T cell proliferation [GO:0042102]; positive regulation of T-helper 1 cell differentiation [GO:0045627]; positive regulation of vesicle fusion [GO:0031340]; positive regulation of wound healing [GO:0090303]; regulation of cell shape [GO:0008360]; regulation of hormone secretion [GO:0046883]; regulation of inflammatory response [GO:0050727]; regulation of interleukin-1 production [GO:0032652]; regulation of leukocyte migration [GO:0002685]	basolateral plasma membrane [GO:0016323]; cell surface [GO:0009986]; cornified envelope [GO:0001533]; cytoplasmic vesicle membrane [GO:0030659]; cytosol [GO:0005829]; endosome [GO:0005768]; extracellular exosome [GO:0070062]; lateral plasma membrane [GO:0016328]; motile cilium [GO:0031514]; nucleoplasm [GO:0005654]; phagocytic cup [GO:0001891]; sarcolemma [GO:0042383]	binding [GO:0005544]; calcium-dependent protein binding [GO:0048306]; phospholipase A2 inhibitor activity [GO:0019834]
A0A8I3P7T5	Annexin	ANXA2		Basement membrane [GO:0005604]; extracellular region [GO:0005576]	Calcium ion binding [GO:0005509]; calcium-dependent phospholipid binding [GO:0005544]; cytoskeletal

					protein binding [GO:0008092]; phospholipase inhibitor activity [GO:0004859]
A0A0F7RNT6	Annexin	ANXA5	Negative regulation of coagulation [GO:0050819]	Endothelial microparticle [GO:0072563]; external side of plasma membrane [GO:0009897]	Calcium ion binding [GO:0005509]; calcium-dependent phospholipid binding [GO:0005544]; phosphatidylserine binding [GO:0001786]
A0A8I3N098	Annexin	ANXA6	Apoptotic signaling pathway [GO:0097190]; calcium ion transport [GO:0006816]; mitochondrial calcium ion homeostasis [GO:0051560]; regulation of muscle contraction [GO:0006937]	Late endosome membrane [GO:0031902]; lysosomal membrane [GO:0005765]; melanosome [GO:0042470]; mitochondrion [GO:0005739]; perinuclear region of cytoplasm [GO:0048471]	Calcium ion binding [GO:0005509]; calcium-dependent phospholipid binding [GO:0005544]; calcium-dependent protein binding [GO:0048306]; cholesterol binding [GO:0015485]; GTP binding [GO:0005525]; identical protein binding [GO:0042802]; ligand-gated monoatomic ion channel activity [GO:0015276]
A0A8I3N2W3	Annexin	ANXA8L1			Calcium ion binding [GO:0005509]; calcium-dependent phospholipid binding [GO:0005544]
A0A8I3MQE3	AP-3 complex subunit beta	AP3B2	Intracellular protein transport [GO:0006886]; vesicle-mediated transport [GO:0016192]	AP-3 adaptor complex [GO:0030123]; clathrin-coated vesicle membrane [GO:0030665]	
A0A8I3MSG4	Apolipoprotein a-i (apolipoprotein a1)	APOA1	Adrenal gland development [GO:0030325]; blood vessel endothelial cell migration [GO:0043534]; cellular response to lipoprotein particle stimulus [GO:0071402]; cholesterol biosynthetic process [GO:0006695]; cholesterol efflux [GO:0033344]; cholesterol homeostasis [GO:0042632]; cholesterol import [GO:0070508]; endothelial cell proliferation [GO:0001935]; G protein-coupled receptor signaling pathway [GO:0007186]; glucocorticoid metabolic process [GO:0008211]; high-density lipoprotein particle assembly [GO:0034380]; high-density lipoprotein particle remodeling [GO:0034375]; integrin-mediated signaling pathway [GO:0007229]; lipid storage [GO:0019915];	Endocytic vesicle [GO:0030139]; spherical high-density lipoprotein particle [GO:0034366]; very-low-density lipoprotein particle [GO:0034361]	Amyloid-beta binding [GO:0001540]; apolipoprotein A-I receptor binding [GO:0034191]; chemorepellent activity [GO:0045499]; cholesterol binding [GO:0015485]; cholesterol transfer activity [GO:0120020]; enzyme binding [GO:0019899]; heat shock protein binding [GO:0031072]; high-density lipoprotein particle binding [GO:0008035]; high-density lipoprotein particle receptor binding [GO:0070653]; identical protein binding [GO:0042802]; phosphatidylcholine-sterol O-acyltransferase activator activity

			lipoprotein biosynthetic process [GO:0042158]; negative regulation of cell adhesion molecule production [GO:0060354]; negative regulation of cytokine production involved in immune response [GO:0002719]; negative regulation of heterotypic cell-cell adhesion [GO:0034115]; negative regulation of inflammatory response [GO:0050728]; negative regulation of interleukin-1 beta production [GO:0032691]; negative regulation of tumor necrosis factor-mediated signaling pathway [GO:0010804]; negative regulation of very-low-density lipoprotein particle remodeling [GO:0010903]; phosphatidylcholine biosynthetic process [GO:0006656]; phospholipid efflux [GO:0033700]; phospholipid homeostasis [GO:0055091]; positive regulation of cholesterol efflux [GO:0010875]; positive regulation of cholesterol metabolic process [GO:0090205]; positive regulation of phagocytosis [GO:0050766]; positive regulation of phospholipid efflux [GO:1902995]; positive regulation of Rho protein signal transduction [GO:0035025]; positive regulation of stress fiber assembly [GO:0051496]; positive regulation of substrate adhesion-dependent cell spreading [GO:1900026]; protein stabilization [GO:0050821]; regulation of Cdc42 protein signal transduction [GO:0032489]; regulation of intestinal cholesterol absorption [GO:0030300]; reverse cholesterol transport [GO:0043691]; triglyceride homeostasis [GO:0070328]; vitamin transport [GO:0051180]		[GO:0060228]; phospholipid binding [GO:0005543]
A0A8I3PXM1	Arylsulfatase A	ARSA			Hydrolase activity [GO:0016787]
A0A8I3NT22	ATP binding cassette subfamily C member 8	ABCC8	Potassium ion transport [GO:0006813]	Plasma membrane [GO:0005886]; protein-containing complex [GO:0032991]	ABC-type transporter activity [GO:0140359]; ATP binding [GO:0005524]; ATP hydrolysis

					activity [GO:0016887]; sulfonylurea receptor activity [GO:0008281]
A0A1B1X468	ATP synthase subunit beta (EC 7.1.2.2)	PTGES3	Angiogenesis [GO:0001525]; cellular response to interleukin-7 [GO:0098761]; lipid metabolic process [GO:0006629]; negative regulation of cell adhesion involved in substrate-bound cell migration [GO:0006933]; positive regulation of blood vessel endothelial cell migration [GO:0043536]; proton motive force-driven mitochondrial ATP synthesis [GO:0042776]; regulation of intracellular ph [GO:0051453]	Cell surface [GO:0009986]; mitochondrial membrane [GO:0031966]; mitochondrial nucleoid [GO:0042645]; plasma membrane [GO:0005886]; proton-transporting ATP synthase complex, catalytic core F(1) [GO:0045261]	Angiostatin binding [GO:0043532]; ATP binding [GO:0005524]; ATP hydrolysis activity [GO:0016887]; MHC class I protein binding [GO:0042288]; proton-transporting ATP synthase activity, rotational mechanism [GO:0046933]; proton-transporting atpase activity, rotational mechanism [GO:0046961]
A0A8I3PX58	Sodium/potassium-transporting atpase subunit beta-3 (Sodium/potassium-dependent atpase subunit beta-3)	ATP1B3	Potassium ion transport [GO:0006813]; sodium ion transport [GO:0006814]	Apical plasma membrane [GO:0016324]; basolateral plasma membrane [GO:0016323]; sodium:potassium-exchanging atpase complex [GO:0005890]	
A0A8I3MY15	Beta-hexosaminidase (EC 3.2.1.52)	HEXB	Astrocyte cell migration [GO:0043615]; chondroitin sulfate catabolic process [GO:0030207]; dermatan sulfate catabolic process [GO:0030209]; ganglioside catabolic process [GO:0006689]; hyaluronan catabolic process [GO:0030214]; intracellular calcium ion homeostasis [GO:0006874]; lipid storage [GO:0019915]; locomotory behavior [GO:0007626]; lysosome organization [GO:0007040]; maintenance of location in cell [GO:0051651]; male courtship behavior [GO:0008049]; myelination [GO:0042552]; neuromuscular process controlling balance [GO:0050885]; neuron cellular homeostasis [GO:0070050]; oligosaccharide catabolic process [GO:0009313]; oogenesis [GO:0048477]; penetration of zona pellucida [GO:0007341]; phospholipid biosynthetic process [GO:0008654]; positive regulation of transcription by RNA polymerase II [GO:0045944]; regulation of cell shape [GO:0008360]; sensory perception of sound	Acrosomal vesicle [GO:0001669]; azurophil granule [GO:0042582]; beta-N-acetylhexosaminidase complex [GO:1905379]; cortical granule [GO:0060473]; extracellular space [GO:0005615]; membrane [GO:0016020]	Acetylglucosaminyltransferase activity [GO:0008375]; beta-N-acetylhexosaminidase activity [GO:0004563]; identical protein binding [GO:0042802]

			[GO:0007605]; skeletal system development [GO:0001501]		
A0A8I3PLT1	Bifunctional purine biosynthesis protein ATIC (EC 2.1.2.3) (EC 3.5.4.10) (AICAR transformylase/inosine monophosphate cyclohydrolase)	ATIC	'De novo' IMP biosynthetic process [GO:0006189]	Cytosol [GO:0005829]	IMP cyclohydrolase activity [GO:0003937]; phosphoribosylaminoimidazolecarboxamide formyltransferase activity [GO:0004643]
A0A8I3N2B4	BMERB domain containing 1	BMERB1	Cell motility involved in cerebral cortex radial glia guided migration [GO:0021814]; microtubule depolymerization [GO:0007019]; negative regulation of cell motility involved in cerebral cortex radial glia guided migration [GO:0021822]; negative regulation of microtubule depolymerization [GO:0007026]	Microtubule cytoskeleton [GO:0015630]	
A0A8I3QW85	B-Raf proto-oncogene, serine/threonine kinase	BRAF	Signal transduction [GO:0007165]		
A0A8I3NUH3	C2 domain-containing protein	DOC2B	Calcium ion-regulated exocytosis of neurotransmitter [GO:0048791]; calcium-dependent activation of synaptic vesicle fusion [GO:0099502]; positive regulation of insulin secretion [GO:0032024]; protein localization [GO:0008104]; spontaneous neurotransmitter secretion [GO:0061669]	Cytoplasm [GO:0005737]; plasma membrane [GO:0005886]; presynapse [GO:0098793]	Calcium ion binding [GO:0005509]; syntaxin binding [GO:0019905]
A0A8I3S2T1	Calcium/calmodulin-dependent protein kinase (EC 2.7.11.17)	CAMKK1			ATP binding [GO:0005524]; protein serine/threonine kinase activity [GO:0004674]
A0A8I3PE55	Calcium-transporting atpase (EC 7.2.2.10)	ATP2B3		Plasma membrane [GO:0005886]	ATP binding [GO:0005524]; ATP hydrolysis activity [GO:0016887]; calmodulin binding [GO:0005516]; P-type calcium transporter activity [GO:0005388]
A0A8I3NLW4	Catenin alpha-2 (Alpha N-catenin)	CTNNA2	Cell adhesion [GO:0007155]; cell differentiation [GO:0030154]	Actin cytoskeleton [GO:0015629]; adherens junction [GO:0005912]; axon [GO:0030424]; cytoplasm	Actin filament binding [GO:0051015]; cadherin binding

				[GO:0005737]; nucleus [GO:0005634]; plasma membrane [GO:0005886]	[GO:0045296]; structural molecule activity [GO:0005198]
Q6TN20	Cathelicidin	CAMP	Amyloid fibril formation [GO:1990000]; antibacterial humoral response [GO:0019731]; antimicrobial humoral immune response mediated by antimicrobial peptide [GO:0061844]; defense response to Gram-negative bacterium [GO:0050829]; defense response to Gram-positive bacterium [GO:0050830]; innate immune response [GO:0045087]; innate immune response in mucosa [GO:0002227]; killing by host of symbiont cells [GO:0051873]; neutrophil activation [GO:0042119]	Extracellular space [GO:0005615]; specific granule [GO:0042581]	Lipopolysaccharide binding [GO:0001530]
A0A8I3P7H9	Cathepsin B (EC 3.4.22.1)	CTSB	Proteolysis [GO:0006508]	Apical plasma membrane [GO:0016324]; lysosome [GO:0005764]	Cysteine-type endopeptidase activity [GO:0004197]
A0A8I3NPI1	Cathepsin F	CTSF	Proteolysis [GO:0006508]	Endoplasmic reticulum [GO:0005783]; lysosome [GO:0005764]; plasma membrane [GO:0005886]	Cysteine-type peptidase activity [GO:0008234]
A0A8I3P050	Cdk5 and Abl enzyme substrate 1	CABLES1	Regulation of cell cycle [GO:0051726]	Cytosol [GO:0005829]	
A0A8I3Q094	Centrosomal protein 112	CEP112		Centrosome [GO:0005813]	
A0A8I3PDZ6	Centrosomal protein 170B	CEP170B			
A0A8I3MQ18	Chromosome 4 open reading frame 50	C4orf50			
A0A8I3PEF5	Chromosome 9 c9orf78 homolog	C9H9orf78		Nucleus [GO:0005634]	
A0A8I3N305	Citrate synthase	CS	Citrate metabolic process [GO:0006101]; tricarboxylic acid cycle [GO:0006099]	Mitochondrial matrix [GO:0005759]	Citrate (Si)-synthase activity [GO:0004108]
A0A8I3NF26	Clusterin	CLU		Chromaffin granule [GO:0042583]; cytosol [GO:0005829]; endoplasmic reticulum [GO:0005783]; extracellular region [GO:0005576]; mitochondrial membrane	

				[GO:0031966]; nucleus [GO:0005634]; perinuclear region of cytoplasm [GO:0048471]	
A0A8I3MD34	ADF-H domain-containing protein	LOC102153978	Actin filament depolymerization [GO:0030042]	Actin cytoskeleton [GO:0015629]; lamellipodium membrane [GO:0031258]; nuclear matrix [GO:0016363]; ruffle membrane [GO:0032587]	Actin filament binding [GO:0051015]
A0A8I3MLW3	Collagen beta(1-O)galactosyltransferase 2	COLGALT2			
A0A8I3NJ53	Cullin 3	CUL3	Protein ubiquitination [GO:0016567]; ubiquitin-dependent protein catabolic process [GO:0006511]	Cullin-RING ubiquitin ligase complex [GO:0031461]	Ubiquitin protein ligase binding [GO:0031625]
A0A8I3NXJ3	C-X-C chemokine receptor type 3 (Interferon-inducible protein 10 receptor)	CXCR3	Angiogenesis [GO:0001525]; chemotaxis [GO:0006935]; inflammatory response [GO:0006954]; regulation of cell adhesion [GO:0030155]; regulation of leukocyte migration [GO:0002685]	External side of plasma membrane [GO:0009897]	C-X-C chemokine receptor activity [GO:0016494]; chemokine binding [GO:0019956]
A0A8I3PQR7	Cyclin dependent kinase 18	CDK18			ATP binding [GO:0005524]; protein serine/threonine kinase activity [GO:0004674]
A0A8I3NW54	Cystatin B	CSTB	Adult locomotory behavior [GO:0008344]; amyloid fibril formation [GO:1990000]; negative regulation of proteolysis [GO:0045861]	Cytosol [GO:0005829]; extracellular space [GO:0005615]; nucleolus [GO:0005730]	Cysteine-type endopeptidase inhibitor activity [GO:0004869]; protease binding [GO:0002020]
A0A8I3QNP3	Uncharacterized protein	LOC607874			Cysteine-type endopeptidase inhibitor activity [GO:0004869]
A0A8I3PN88	DDB1 and CUL4 associated factor 15	DCAF15	Protein polyubiquitination [GO:0000209]; regulation of natural killer cell activation [GO:0032814]	Cul4-RING E3 ubiquitin ligase complex [GO:0080008]	Small molecule binding [GO:0036094]
A0A8I3PSK5	DEP domain containing 5, GATOR1 subcomplex subunit	DEPDC5	Cellular response to amino acid starvation [GO:0034198]; intracellular signal transduction [GO:0035556]; negative regulation of TORC1 signaling [GO:1904262]	Cul3-RING ubiquitin ligase complex [GO:0031463]; cytosol [GO:0005829]; GATOR1 complex [GO:1990130]; lysosomal membrane [GO:0005765]; perinuclear region of cytoplasm [GO:0048471]	Gtpase activator activity [GO:0005096]; protein-containing complex binding [GO:0044877]; small gtpase binding [GO:0031267]
A0A8I3N6W4	Dexd/H-box helicase 60	DDX60			ATP binding [GO:0005524]; helicase activity [GO:0004386]; hydrolase

					activity [GO:0016787]; nucleic acid binding [GO:0003676]
A0A8I3S7J9	DNA-(apurinic or apyrimidinic site) endonuclease (EC 3.1.-.-)	APEX2	DNA recombination [GO:0006310]; DNA repair [GO:0006281]	Fibrillar center [GO:0001650]; mitochondrion [GO:0005739]; nucleoplasm [GO:0005654]	DNA binding [GO:0003677]; endonuclease activity [GO:0004519]; exonuclease activity [GO:0004527]; zinc ion binding [GO:0008270]
A0A8I3RZT8	DNA-directed RNA polymerase subunit beta (EC 2.7.7.6)	POLR3B	DNA-templated transcription [GO:0006351]	DNA-directed RNA polymerase complex [GO:0000428]; nucleus [GO:0005634]	DNA binding [GO:0003677]; DNA-directed 5'-3' RNA polymerase activity [GO:0003899]; metal ion binding [GO:0046872]; ribonucleoside binding [GO:0032549]
A0A8I3NGS6	DOP1 leucine zipper like protein A	DOP1A	Golgi to endosome transport [GO:0006895]; protein transport [GO:0015031]	Cytosol [GO:0005829]	
A0A8I3P241	Doublecortin domain-containing protein	RP1L1	Cell projection organization [GO:0030030]; intracellular signal transduction [GO:0035556]; visual perception [GO:0007601]	Cytoplasm [GO:0005737]; cytoskeleton [GO:0005856]; photoreceptor outer segment [GO:0001750]	
A0A8I3Q4C7	Dynein light chain	DYNLL1	Apoptotic process [GO:0006915]; DNA damage response [GO:0006974]; microtubule-based process [GO:0007017]	Centrosome [GO:0005813]; chromosome [GO:0005694]; dynein complex [GO:0030286]; microtubule [GO:0005874]; mitochondrion [GO:0005739]; nucleus [GO:0005634]	
A0A8I3MMN1	EF-hand calcium binding domain 7	EFCAB7	Positive regulation of protein import into nucleus [GO:0042307]; positive regulation of protein localization to ciliary membrane [GO:1903569]; positive regulation of transcription by RNA polymerase II [GO:0045944]; protein localization to motile cilium [GO:0120229]; regulation of smoothened signaling pathway [GO:0008589]	Ciliary membrane [GO:0060170]; cytoplasmic side of plasma membrane [GO:0009898]; plasma membrane protein complex [GO:0098797]	Calcium ion binding [GO:0005509]
A0A8I3NGL9	EGF-like domain-containing protein	TDGF1	Signal transduction [GO:0007165]		
A0A8I3NCW4	Elongation factor 1-gamma (eef-1B gamma)	EEF1G			Translation elongation factor activity [GO:0003746]

A0A8I3PAX2	Endoplasmic reticulum resident protein 29	ERP29	Negative regulation of gene expression [GO:0010629]; negative regulation of protein secretion [GO:0050709]; positive regulation of gene expression [GO:0010628]; protein secretion [GO:0009306]; regulation of endoplasmic reticulum stress-induced intrinsic apoptotic signaling pathway [GO:1902235]	Cell surface [GO:0009986]; endoplasmic reticulum lumen [GO:0005788]; melanosome [GO:0042470]	
A0A8I3RQG5	Epididymal sperm binding protein 1	ELSPBP1	Single fertilization [GO:0007338]	Extracellular region [GO:0005576]	
A0A8I3NZP7	Euchromatic histone lysine methyltransferase 2	EHMT2	Behavioral response to cocaine [GO:0048148]; cellular response to cocaine [GO:0071314]; cellular response to starvation [GO:0009267]; DNA methylation-dependent constitutive heterochromatin formation [GO:0006346]; fertilization [GO:0009566]; methylation [GO:0032259]; negative regulation of autophagosome assembly [GO:1902902]; negative regulation of gene expression via chromosomal cpg island methylation [GO:0044027]; negative regulation of transcription by RNA polymerase II [GO:0000122]; neuron fate specification [GO:0048665]; oocyte development [GO:0048599]; organ growth [GO:0035265]; phenotypic switching [GO:0036166]; regulation of DNA replication [GO:0006275]; spermatid development [GO:0007286]; synaptonemal complex assembly [GO:0007130]	Chromatin [GO:0000785]; nuclear speck [GO:0016607]	C2H2 zinc finger domain binding [GO:0070742]; histone H3K27 methyltransferase activity [GO:0046976]; histone H3K56 methyltransferase activity [GO:0140759]; histone H3K9 methyltransferase activity [GO:0046974]; p53 binding [GO:0002039]; promoter-specific chromatin binding [GO:1990841]; RNA polymerase II transcription regulatory region sequence-specific DNA binding [GO:0000977]; transcription corepressor binding [GO:0001222]; zinc ion binding [GO:0008270]
A0A8I3NNE8	Elongation factor 1-delta	EEF1D		Eukaryotic translation elongation factor 1 complex [GO:0005853]	Translation elongation factor activity [GO:0003746]
A0A8I3MIA3	Evc ciliary complex subunit 2	EVC2	Smoothened signaling pathway [GO:0007224]	Ciliary membrane [GO:0060170]; cytoplasm [GO:0005737]; cytoskeleton [GO:0005856]	
A0A8I3N536	F-actin-capping protein subunit alpha-2 (capz alpha-2)	CAPZA2	Barbed-end actin filament capping [GO:0051016]	Brush border [GO:0005903]; cortical cytoskeleton [GO:0030863]; F-actin capping protein complex [GO:0008290]; membrane [GO:0016020]	Actin binding [GO:0003779]

A0A8I3MHS5	F-actin-capping protein subunit beta	CAPZB	Actin polymerization or depolymerization [GO:0008154]; barbed-end actin filament capping [GO:0051016]; lamellipodium assembly [GO:0030032]; regulation of cell morphogenesis [GO:0022604]	Brush border [GO:0005903]; cortical cytoskeleton [GO:0030863]; F-actin capping protein complex [GO:0008290]; hippocampal mossy fiber to CA3 synapse [GO:0098686]; lamellipodium [GO:0030027]; membrane [GO:0016020]; postsynaptic density [GO:0014069]; Schaffer collateral - CA1 synapse [GO:0098685]; sperm head-tail coupling apparatus [GO:0120212]; WASH complex [GO:0071203]	Actin binding [GO:0003779]
A0A8I3P528	FERM and PDZ domain containing 3	FRMPD3		Cytoskeleton [GO:0005856]	
A0A8I3NVK3	Ferritin		Intracellular iron ion homeostasis [GO:0006879]; iron ion transport [GO:0006826]	Autolysosome [GO:0044754]	Ferric iron binding [GO:0008199]
A0A8I3MTX3	FHF complex subunit HOOK-interacting protein 1B (FTS- and Hook-interacting protein)	FHIP1B	Protein transport [GO:0015031]		
A0A8I3SCU1	Fibronectin leucine rich transmembrane protein 1	FLRT1	Cell adhesion [GO:0007155]; dendrite development [GO:0016358]; fibroblast growth factor receptor signaling pathway [GO:0008543]; neuron projection extension [GO:1990138]; positive regulation of synapse assembly [GO:0051965]	Cell-cell junction [GO:0005911]; cytoplasmic vesicle [GO:0031410]; endoplasmic reticulum membrane [GO:0005789]; extracellular space [GO:0005615]; focal adhesion [GO:0005925]; neuron projection terminus [GO:0044306]; neuronal cell body membrane [GO:0032809]; perinuclear region of cytoplasm [GO:0048471]	Fibroblast growth factor receptor binding [GO:0005104]
A0A8I3MGH3	Fibronectin type III domain containing 1	FNDC1			
A0A8I3QCK4	Fibronectin type-III domain-containing protein	FANK1			
A0A8I3MJP4	Fructose-bisphosphate	ALDOA	Glycolytic process [GO:0006096]	I band [GO:0031674]; M band [GO:0031430]	Fructose-bisphosphate aldolase activity [GO:0004332]

	aldolase (EC 4.1.2.13)				
A0A8I3PHH5	Fructose-bisphosphate aldolase (EC 4.1.2.13)		Glycolytic process [GO:0006096]	I band [GO:0031674]; M band [GO:0031430]	Fructose-bisphosphate aldolase activity [GO:0004332]
A0A8I3NHK6	Galectin-3-binding protein (Lectin galactoside-binding soluble 3-binding protein)	CANT1	Cell adhesion [GO:0007155]	Extracellular region [GO:0005576]; membrane [GO:0016020]	Calcium ion binding [GO:0005509]; metalloendopeptidase inhibitor activity [GO:0008191]; nucleoside diphosphate phosphatase activity [GO:0017110]
A0A8I3P7W8	Gamma-secretase activating protein	GSAP			
A0A8I3NQB9	Gelsolin (ADF) (Actin-depolymerizing factor)	GSN	Actin filament severing [GO:0051014]; barbed-end actin filament capping [GO:0051016]; cilium assembly [GO:0060271]	Cytoplasm [GO:0005737]; cytoskeleton [GO:0005856]	Actin filament binding [GO:0051015]
A0A8I3PTI8	Gliomedin	GLDN	Clustering of voltage-gated sodium channels [GO:0045162]; microvillus organization [GO:0032528]	Cell surface [GO:0009986]; extracellular space [GO:0005615]; plasma membrane [GO:0005886]	Protein binding involved in heterotypic cell-cell adhesion [GO:0086080]
A0A8I3MPB5	Glucose-6-phosphate isomerase (EC 5.3.1.9)	GPI	Gluconeogenesis [GO:0006094]; glycolytic process [GO:0006096]		Carbohydrate derivative binding [GO:0097367]; glucose-6-phosphate isomerase activity [GO:0004347]
A0A8I3PAT3	Glutathione peroxidase	GPX5	Response to oxidative stress [GO:0006979]	Extracellular region [GO:0005576]	Peroxidase activity [GO:0004601]
A0A8I3N2E9	Glutathione S-transferase (EC 2.5.1.18)	GSTM3		Cytoplasm [GO:0005737]	Glutathione transferase activity [GO:0004364]; identical protein binding [GO:0042802]
A0A8I3Q407	Glyceraldehyde-3-phosphate dehydrogenase (EC 1.2.1.12)	LOC477441	Apoptotic process [GO:0006915]; glucose metabolic process [GO:0006006]; glycolytic process [GO:0006096]; regulation of translation [GO:0006417]	Cytoskeleton [GO:0005856]; cytosol [GO:0005829]; nucleus [GO:0005634]	Glyceraldehyde-3-phosphate dehydrogenase (NAD+) (phosphorylating) activity [GO:0004365]; NAD binding [GO:0051287]; NADP binding [GO:0050661]; transferase activity [GO:0016740]
A0A8I3QC15	Glyoxalase domain containing 5	GLOD5			
A0A8I3MPF1	Glypican 2	GPC2	Regulation of signal transduction [GO:0009966]	Collagen-containing extracellular matrix [GO:0062023]; endoplasmic	

				reticulum [GO:0005783]; plasma membrane [GO:0005886]; side of membrane [GO:0098552]	
A0A8I3MXM3	GMP reductase (GMPR) (EC 1.7.1.7) (Guanosine 5'-monophosphate oxidoreductase) (Guanosine monophosphate reductase)	GMPR2 GMPR	Purine nucleobase metabolic process [GO:0006144]; purine nucleotide metabolic process [GO:0006163]	GMP reductase complex [GO:1902560]	GMP reductase activity [GO:0003920]; metal ion binding [GO:0046872]
A0A8I3MEA4	Glyceraldehyde-3-phosphate dehydrogenase (EC 1.2.1.12) (Peptidyl-cysteine S-nitrosylase GAPDH)		Apoptotic process [GO:0006915]; glycolytic process [GO:0006096]; regulation of translation [GO:0006417]	Cytoskeleton [GO:0005856]; cytosol [GO:0005829]; nucleus [GO:0005634]	NAD binding [GO:0051287]; oxidoreductase activity, acting on the aldehyde or oxo group of donors, NAD or NADP as acceptor [GO:0016620]; transferase activity [GO:0016740]
A0A8I3PSR9	GRB10 interacting GYF protein 2	GIGYF2	Adult locomotory behavior [GO:0008344]; feeding behavior [GO:0007631]; homeostasis of number of cells within a tissue [GO:0048873]; insulin-like growth factor receptor signaling pathway [GO:0048009]; mitotic G1 DNA damage checkpoint signaling [GO:0031571]; mrna destabilization [GO:0061157]; multicellular organism growth [GO:0035264]; musculoskeletal movement [GO:0050881]; negative regulation of translational initiation [GO:0045947]; negative regulation of type I interferon-mediated signaling pathway [GO:0060339]; neuromuscular process controlling balance [GO:0050885]; post-embryonic development [GO:0009791]; post-transcriptional gene silencing [GO:0016441]; rescue of stalled ribosome [GO:0072344]; spinal cord motor neuron differentiation [GO:0021522]	Cytoplasmic stress granule [GO:0010494]; cytosol [GO:0005829]; endoplasmic reticulum [GO:0005783]; endosome [GO:0005768]; Golgi apparatus [GO:0005794]; membrane [GO:0016020]; perikaryon [GO:0043204]; protein-containing complex [GO:0032991]; proximal dendrite [GO:1990635]	Molecular adaptor activity [GO:0060090]; proline-rich region binding [GO:0070064]
A0A8I3PV53	H1.2 linker histone, cluster member	H1-2	Negative regulation of transcription by RNA polymerase II [GO:0000122]; nucleosome assembly [GO:0006334]	Euchromatin [GO:0000791]; nucleosome [GO:0000786]; nucleus [GO:0005634]	DNA binding [GO:0003677]; structural constituent of chromatin [GO:0030527]

A0A8I3N9V3	Histidine kinase/HSP90-like atpase domain-containing protein	HSP90AB1	Cellular response to heat [GO:0034605]; cellular response to interleukin-4 [GO:0071353]; chaperone-mediated protein complex assembly [GO:0051131]; chaperone-mediated protein folding [GO:0061077]; negative regulation of apoptotic process [GO:0043066]; negative regulation of proteasomal ubiquitin-dependent protein catabolic process [GO:0032435]; placenta development [GO:0001890]; positive regulation of cell differentiation [GO:0045597]; positive regulation of protein localization to cell surface [GO:2000010]; positive regulation of transforming growth factor beta receptor signaling pathway [GO:0030511]; regulation of cell cycle [GO:0051726]; regulation of protein ubiquitination [GO:0031396]; supramolecular fiber organization [GO:0097435]; telomerase holoenzyme complex assembly [GO:1905323]; telomere maintenance via telomerase [GO:0007004]; virion attachment to host cell [GO:0019062]	Aryl hydrocarbon receptor complex [GO:0034751]; axonal growth cone [GO:0044295]; COP9 signalosome [GO:0008180]; cytosol [GO:0005829]; dendritic growth cone [GO:0044294]; dynein axonemal particle [GO:0120293]; extracellular region [GO:0005576]; HSP90-CDC37 chaperone complex [GO:1990565]; melanosome [GO:0042470]; neuronal cell body [GO:0043025]; perinuclear region of cytoplasm [GO:0048471]	ATP binding [GO:0005524]; ATP hydrolysis activity [GO:0016887]; ATP-dependent protein binding [GO:0043008]; ATP-dependent protein folding chaperone [GO:0140662]; disordered domain specific binding [GO:0097718]; DNA polymerase binding [GO:0070182]; double-stranded RNA binding [GO:0003725]; heat shock protein binding [GO:0031072]; histone deacetylase binding [GO:0042826]; histone methyltransferase binding [GO:1990226]; peptide binding [GO:0042277]; protein homodimerization activity [GO:0042803]; protein kinase binding [GO:0019901]; protein kinase regulator activity [GO:0019887]; protein phosphatase activator activity [GO:0072542]; receptor ligand inhibitor activity [GO:0141069]; tau protein binding [GO:0048156]; ubiquitin protein ligase binding [GO:0031625]; unfolded protein binding [GO:0051082]
A0A8I3NV93	HAUS augmin like complex subunit 6	HAUS6	Centrosome cycle [GO:0007098]; spindle assembly [GO:0051225]	Centrosome [GO:0005813]; HAUS complex [GO:0070652]; mitotic spindle microtubule [GO:1990498]	
A0A8I3MR78	Heat shock protein 90 alpha family class A member 1	HSP90AA1	Activation of innate immune response [GO:0002218]; cellular response to virus [GO:0098586]; chaperone-mediated protein complex assembly [GO:0051131]; neurofibrillary tangle assembly [GO:1902988]; positive regulation of defense response to virus by host [GO:0002230]; positive regulation of interferon-beta production [GO:0032728]; positive regulation of protein catabolic process [GO:0045732]; positive regulation of protein	Cytosol [GO:0005829]; melanosome [GO:0042470]; mitochondrion [GO:0005739]; nucleoplasm [GO:0005654]; protein-containing complex [GO:0032991]	ATP binding [GO:0005524]; ATP hydrolysis activity [GO:0016887]; ATP-dependent protein folding chaperone [GO:0140662]; disordered domain specific binding [GO:0097718]; DNA polymerase binding [GO:0070182]; gtpase binding [GO:0051020]; histone deacetylase binding [GO:0042826]; nitric-oxide synthase regulator

			polymerization [GO:0032273]; protein insertion into mitochondrial outer membrane [GO:0045040]; protein stabilization [GO:0050821]; regulation of apoptotic process [GO:0042981]; regulation of protein ubiquitination [GO:0031396]; telomerase holoenzyme complex assembly [GO:1905323]; telomere maintenance via telomerase [GO:0007004]		activity [GO:0030235]; protein homodimerization activity [GO:0042803]; protein tyrosine kinase binding [GO:1990782]; scaffold protein binding [GO:0097110]; tau protein binding [GO:0048156]; TPR domain binding [GO:0030911]; ubiquitin protein ligase binding [GO:0031625]; unfolded protein binding [GO:0051082]
A0A8I3PZH8	Heat shock protein 90 alpha family class A member 1			Cytoplasm [GO:0005737]	ATP binding [GO:0005524]; ATP hydrolysis activity [GO:0016887]; ATP-dependent protein folding chaperone [GO:0140662]; unfolded protein binding [GO:0051082]
A0A8I3NA23	Heat shock protein 90 beta family member 1	HSP90B1		Melanosome [GO:0042470]; sarcoplasmic reticulum lumen [GO:0033018]	ATP binding [GO:0005524]; ATP hydrolysis activity [GO:0016887]; ATP-dependent protein folding chaperone [GO:0140662]; unfolded protein binding [GO:0051082]
A0A8I3NE39	Heat shock protein family A (Hsp70) member 1 like	HSPA1L	Binding of sperm to zona pellucida [GO:0007339]; positive regulation of protein targeting to mitochondrion [GO:1903955]; protein refolding [GO:0042026]	Cell body [GO:0044297]; COP9 signalosome [GO:0008180]; cytosol [GO:0005829]; zona pellucida receptor complex [GO:0002199]	ATP binding [GO:0005524]; ATP-dependent protein folding chaperone [GO:0140662]; heat shock protein binding [GO:0031072]; ubiquitin protein ligase binding [GO:0031625]; unfolded protein binding [GO:0051082]
A0A8I3N1B1	Heat shock protein family A (Hsp70) member 2	HSPA2	Male meiosis I [GO:0007141]; negative regulation of inclusion body assembly [GO:0090084]; positive regulation of G2/M transition of mitotic cell cycle [GO:0010971]; protein refolding [GO:0042026]; spermatid development [GO:0007286]; synaptonemal complex disassembly [GO:0070194]	Catsper complex [GO:0036128]; cell surface [GO:0009986]; cytosol [GO:0005829]; male germ cell nucleus [GO:0001673]; meiotic spindle [GO:0072687]; synaptonemal complex [GO:0000795]	ATP binding [GO:0005524]; ATP-dependent protein folding chaperone [GO:0140662]; disordered domain specific binding [GO:0097718]; enzyme binding [GO:0019899]; glycolipid binding [GO:0051861]; unfolded protein binding [GO:0051082]
A0A8I3NXH9	Heat shock protein family A (Hsp70) member 4 like	HSPA4L			ATP binding [GO:0005524]; ATP-dependent protein folding chaperone [GO:0140662]

A0A8I3NK47	Heat shock protein family A (Hsp70) member 8	LOC607182	Clathrin coat disassembly [GO:0072318]	Spliceosomal complex [GO:0005681]	ATP binding [GO:0005524]; ATP-dependent protein folding chaperone [GO:0140662]
A0A8I3NX23	F5/8 type C domain-containing protein	IFT25	Intraciliary transport [GO:0042073]	Cilium [GO:0005929]; intraciliary transport particle B [GO:0030992]	
A0A8I3P9Z8	HECT domain E3 ubiquitin protein ligase 4	HECTD4			Ubiquitin-protein transferase activity [GO:0004842]
A0A8I3PEM1	Histidine decarboxylase (EC 4.1.1.22)	HDC	Amino acid metabolic process [GO:0006520]; carboxylic acid metabolic process [GO:0019752]; catecholamine biosynthetic process [GO:0042423]		Carboxy-lyase activity [GO:0016831]; pyridoxal phosphate binding [GO:0030170]
A0A8I3N371	Histone H2B	H2BC26		Nucleosome [GO:0000786]; nucleus [GO:0005634]	DNA binding [GO:0003677]; protein heterodimerization activity [GO:0046982]; structural constituent of chromatin [GO:0030527]
A0A8I3NXI1	Histone H4			Nucleosome [GO:0000786]; nucleus [GO:0005634]	DNA binding [GO:0003677]; protein heterodimerization activity [GO:0046982]; structural constituent of chromatin [GO:0030527]
A0A8I3MQH3	Hyaluronidase (EC 3.2.1.35)	SPAM1	Carbohydrate metabolic process [GO:0005975]; fusion of sperm to egg plasma membrane involved in single fertilization [GO:0007342]	Plasma membrane [GO:0005886]; side of membrane [GO:0098552]	Hyaluronoglucosaminidase activity [GO:0004415]
A0A8I3PEZ2	Keratin, type II cytoskeletal 8 (Cytokeratin-8) (Keratin-8)			Cytoplasm [GO:0005737]; intermediate filament [GO:0005882]; nuclear matrix [GO:0016363]; nucleoplasm [GO:0005654]	
A0A8I3P2Z9	Ig-like domain-containing protein	KIRREL1		Membrane [GO:0016020]	
A0A8I3P6S7	Ig-like domain-containing protein				
E2RG75	Inactive ribonuclease-like protein 9	RAK1 RNASE9	Defense response to Gram-positive bacterium [GO:0050830]	Extracellular region [GO:0005576]	Nucleic acid binding [GO:0003676]
A0A8I3NAA8	Inositol-1-monophosphatase (EC 3.1.3.25)	IMPA2	Inositol biosynthetic process [GO:0006021]; phosphatidylinositol phosphate biosynthetic process [GO:0046854]		Inositol monophosphate 1-phosphatase activity [GO:0008934]; metal ion binding [GO:0046872]

A0A8I3PC70	Inositol-3-phosphate synthase 1 (EC 5.5.1.4)	ELL	Inositol biosynthetic process [GO:0006021]; phospholipid biosynthetic process [GO:0008654]		Inositol-3-phosphate synthase activity [GO:0004512]
A0A8I3N4M4	Interleukin 18 receptor 1	IL1RL1	Natural killer cell activation [GO:0030101]; negative regulation of cold-induced thermogenesis [GO:0120163]; positive regulation of non-canonical NF-kappab signal transduction [GO:1901224]; positive regulation of T-helper 1 cell cytokine production [GO:2000556]; positive regulation of type II interferon production [GO:0032729]; T-helper 1 cell differentiation [GO:0045063]	Interleukin-18 receptor complex [GO:0045092]	Hydrolase activity [GO:0016787]; interleukin-18 binding [GO:0042007]; interleukin-18 receptor activity [GO:0042008]
A0A8I3MI94	Interleukin family protein	IL24		Extracellular space [GO:0005615]	Cytokine activity [GO:0005125]
A0A8I3N935	Interleukin-1	IL1A	Fever generation [GO:0001660]; immune response [GO:0006955]; positive regulation of cell division [GO:0051781]	Cytosol [GO:0005829]; extracellular space [GO:0005615]; nucleus [GO:0005634]	Cytokine activity [GO:0005125]; interleukin-1 receptor binding [GO:0005149]
A0A8I3ND47	Isoaspartyl peptidase/L-asparaginase (EC 3.4.19.5) (EC 3.5.1.1) (Asparaginase-like protein 1) (Beta-aspartyl-peptidase) (Isoaspartyl dipeptidase) (L-asparagine amidohydrolase)	ASRGL1	Proteolysis [GO:0006508]	Cytoplasm [GO:0005737]	Asparaginase activity [GO:0004067]; peptidase activity [GO:0008233]
A0A8I3NDM6	IZUMO family member 4	IZUMO4			
A0A8I3NM17	Kallikrein 1	KLK1	Proteolysis [GO:0006508]		Serine-type endopeptidase activity [GO:0004252]
A0A1R3UGQ4	Kallikrein A1 (Kallikrein-related peptidase 2)	KLNA1 KLK2	Proteolysis [GO:0006508]		Serine-type endopeptidase activity [GO:0004252]
A0A8I3S8A6	Keratin 73	KRT73		Keratin filament [GO:0045095]	
A0A8I3PMV1	Keratin 74	KRT74	Intermediate filament cytoskeleton organization [GO:0045104]	Cytoplasm [GO:0005737]; keratin filament [GO:0045095]	Keratin filament binding [GO:1990254]

A0A8I3PK78	Keratin 75	KRT75	Hematopoietic progenitor cell differentiation [GO:0002244]	Cornified envelope [GO:0001533]; keratin filament [GO:0045095]	
A0A8I3NL87	Keratin, type I cytoskeletal 10 (Cytokeratin-10) (Keratin-10)	KRT10	Keratinocyte differentiation [GO:0030216]; protein heterotetramerization [GO:0051290]	Cell surface [GO:0009986]; cornified envelope [GO:0001533]; cytoplasm [GO:0005737]; extracellular region [GO:0005576]; keratin filament [GO:0045095]	Protein heterodimerization activity [GO:0046982]; structural constituent of skin epidermis [GO:0030280]
A0A8I3NNW0	Keratin 9	KRT9	Intermediate filament organization [GO:0045109]; skin development [GO:0043588]; spermatogenesis [GO:0007283]	Intermediate filament [GO:0005882]	Structural molecule activity [GO:0005198]
Q6EIY9	Keratin, type II cytoskeletal 1 (Cytokeratin-1) (CK-1) (Epithelial keratin-1) (Keratin-1) (K1) (Type-II keratin Kb1)	KRT1 KER1	Intermediate filament organization [GO:0045109]; keratinization [GO:0031424]; protein heterotetramerization [GO:0051290]	Cytoplasm [GO:0005737]; keratin filament [GO:0045095]; plasma membrane [GO:0005886]	Protein heterodimerization activity [GO:0046982]; structural constituent of skin epidermis [GO:0030280]
A0A8I3NMR3	Lactotransferrin	LTF	Antibacterial humoral response [GO:0019731]; antifungal humoral response [GO:0019732]; antimicrobial humoral immune response mediated by antimicrobial peptide [GO:0061844]; bone morphogenesis [GO:0060349]; defense response to Gram-negative bacterium [GO:0050829]; innate immune response in mucosa [GO:0002227]; iron ion transport [GO:0006826]; killing of cells of another organism [GO:0031640]; negative regulation by host of viral process [GO:0044793]; negative regulation of lipopolysaccharide-mediated signaling pathway [GO:0031665]; negative regulation of single-species biofilm formation in or on host organism [GO:1900229]; negative regulation of viral genome replication [GO:0045071]; ossification [GO:0001503]; positive regulation of canonical NF-kappab signal transduction [GO:0043123]; positive regulation of chondrocyte proliferation [GO:1902732]; positive regulation of osteoblast differentiation [GO:0045669]; positive regulation of osteoblast	Cell surface [GO:0009986]; extracellular space [GO:0005615]; protein-containing complex [GO:0032991]; specific granule [GO:0042581]	Cysteine-type endopeptidase inhibitor activity [GO:0004869]; heparin binding [GO:0008201]; iron ion binding [GO:0005506]; lipopolysaccharide binding [GO:0001530]; membrane destabilizing activity [GO:0140912]; protein serine/threonine kinase activator activity [GO:0043539]; serine-type peptidase activity [GO:0008236]

			proliferation [GO:0033690]; positive regulation of toll-like receptor 4 signaling pathway [GO:0034145]; proteolysis [GO:0006508]; regulation of tumor necrosis factor production [GO:0032680]		
A0A8I3PFE5	Laminin subunit alpha 4	LAMA4	Cell adhesion [GO:0007155]; regulation of cell adhesion [GO:0030155]; regulation of cell migration [GO:0030334]; regulation of embryonic development [GO:0045995]	Basement membrane [GO:0005604]; extracellular region [GO:0005576]	Signaling receptor binding [GO:0005102]
A0A8I3NJC2	LIM domain-containing protein 1	LIMD1	Regulatory ncrna-mediated gene silencing [GO:0031047]	Cytoplasm [GO:0005737]; nucleus [GO:0005634]	Metal ion binding [GO:0046872]
A0A8I3Q9B0	Lipocalin 2	LCN2	Defense response to bacterium [GO:0042742]	Extracellular space [GO:0005615]	Enterobactin binding [GO:1903981]; identical protein binding [GO:0042802]; iron ion binding [GO:0005506]; iron ion sequestering activity [GO:0140315]
H2B3G5	Lipocalin Can f 6.0101 (Allergen Can f 6) (allergen Can f 6.0101)			Extracellular region [GO:0005576]; extracellular space [GO:0005615]	Odorant binding [GO:0005549]; small molecule binding [GO:0036094]
A0A8I3MSE6	Lipocalin like 1	LCNL1			Small molecule binding [GO:0036094]
A0A8I3P2H4	L-lactate dehydrogenase (EC 1.1.1.27)	LDHC	ATP biosynthetic process [GO:0006754]; flagellated sperm motility [GO:0030317]; lactate biosynthetic process from pyruvate [GO:0019244]; pyruvate catabolic process [GO:0042867]	Cytosol [GO:0005829]; motile cilium [GO:0031514]	L-lactate dehydrogenase activity [GO:0004459]
A0A8I3MXC1	Lymphocyte cytosolic protein 1	LCP1	Actin filament bundle assembly [GO:0051017]; cell migration [GO:0016477]; extracellular matrix disassembly [GO:0022617]; positive regulation of podosome assembly [GO:0071803]; protein kinase A signaling [GO:0010737]; regulation of intracellular protein transport [GO:0033157]; T cell activation involved in immune response [GO:0002286]	Actin filament [GO:0005884]; cytosol [GO:0005829]; filopodium [GO:0030175]; focal adhesion [GO:0005925]; glial cell projection [GO:0097386]; perinuclear region of cytoplasm [GO:0048471]; phagocytic cup [GO:0001891]; podosome [GO:0002102]; ruffle [GO:0001726]; stress fiber [GO:0001725]	Actin filament binding [GO:0051015]; calcium ion binding [GO:0005509]; gtpase binding [GO:0051020]; identical protein binding [GO:0042802]; integrin binding [GO:0005178]
P81709	Lysozyme C, spleen isozyme (EC 3.2.1.17) (1,4-beta-N-		Defense response to Gram-negative bacterium [GO:0050829]; defense response to Gram-positive bacterium [GO:0050830]; killing of cells of another organism [GO:0031640]		Lysozyme activity [GO:0003796]

	acetylmuramidase C)				
E2RR82	Lysozyme D1	LOC487083 LYZD1			Lysozyme activity [GO:0003796]
A0A8I3NX55	Lysozyme (EC 3.2.1.17)	LYZ	Defense response to Gram-positive bacterium [GO:0050830]; killing of cells of another organism [GO:0031640]	Extracellular space [GO:0005615]	Identical protein binding [GO:0042802]; lysozyme activity [GO:0003796]
A0A8I3NNU8	Capping actin protein, gelsolin like	CAPG		Centriole [GO:0005814]; cytoplasm [GO:0005737]; Flemming body [GO:0090543]; mitotic spindle [GO:0072686]; nucleolus [GO:0005730]; nucleoplasm [GO:0005654]	Actin filament binding [GO:0051015]; protein domain specific binding [GO:0019904]
A0A8I3NYN6	Uncharacterized protein	MDH1	Malate metabolic process [GO:0006108]		L-malate dehydrogenase (NAD+) activity [GO:0030060]
A0A8I3MS45	Malate dehydrogenase (EC 1.1.1.37)	MDH2 LOC119881628	Malate metabolic process [GO:0006108]; tricarboxylic acid cycle [GO:0006099]		L-malate dehydrogenase (NAD+) activity [GO:0030060]
A0A8I3P3U3	Mannose-6-phosphate isomerase (EC 5.3.1.8) (Phosphohexomutase) (Phosphomannose isomerase)	MPI	Carbohydrate metabolic process [GO:0005975]; GDP-mannose biosynthetic process [GO:0009298]		Mannose-6-phosphate isomerase activity [GO:0004476]; zinc ion binding [GO:0008270]
A0A8I3P478	Matrix metalloproteinase-9 (EC 3.4.24.35) (92 kda gelatinase) (92 kda type IV collagenase) (Gelatinase B)	MMP9	Apoptotic process [GO:0006915]; cell migration [GO:0016477]; cellular response to UV-A [GO:0071492]; collagen catabolic process [GO:0030574]; embryo implantation [GO:0007566]; endodermal cell differentiation [GO:0035987]; extracellular matrix organization [GO:0030198]; negative regulation of epithelial cell differentiation involved in kidney development [GO:2000697]; negative regulation of intrinsic apoptotic signaling pathway [GO:2001243]; positive regulation of apoptotic process [GO:0043065]; positive regulation of epidermal growth factor receptor signaling pathway	Extracellular matrix [GO:0031012]; extracellular space [GO:0005615]	Identical protein binding [GO:0042802]; metalloendopeptidase activity [GO:0004222]; zinc ion binding [GO:0008270]

			[GO:0045742]; positive regulation of keratinocyte migration [GO:0051549]; positive regulation of release of cytochrome c from mitochondria [GO:0090200]; positive regulation of vascular associated smooth muscle cell proliferation [GO:1904707]; proteolysis [GO:0006508]; response to amyloid-beta [GO:1904645]; skeletal system development [GO:0001501]		
A0A8I3Q8P5	MAX dimerization protein MGA	MGA	Positive regulation of DNA-templated transcription [GO:0045893]	MLL1 complex [GO:0071339]	DNA-binding transcription factor activity [GO:0003700]; protein dimerization activity [GO:0046983]; RNA polymerase II cis-regulatory region sequence-specific DNA binding [GO:0000978]
A0A8I3NEL7	Mediator of RNA polymerase II transcription subunit 1 (Mediator complex subunit 1)	MED1	Androgen biosynthetic process [GO:0006702]; angiogenesis [GO:0001525]; animal organ regeneration [GO:0031100]; brain development [GO:0007420]; cell morphogenesis [GO:0000902]; cellular response to epidermal growth factor stimulus [GO:0071364]; cellular response to hepatocyte growth factor stimulus [GO:0035729]; cellular response to thyroid hormone stimulus [GO:0097067]; embryonic heart tube development [GO:0035050]; embryonic hemopoiesis [GO:0035162]; embryonic hindlimb morphogenesis [GO:0035116]; embryonic placenta development [GO:0001892]; enucleate erythrocyte development [GO:0048822]; epithelial cell proliferation involved in mammary gland duct elongation [GO:0060750]; fat cell differentiation [GO:0045444]; G0 to G1 transition [GO:0045023]; hematopoietic stem cell differentiation [GO:0060218]; keratinocyte differentiation [GO:0030216]; lactation [GO:0007595]; lens development in camera-type eye [GO:0002088]; liver development [GO:0001889]; mammary gland branching	Core mediator complex [GO:0070847]; mediator complex [GO:0016592]; nucleolus [GO:0005730]; nucleoplasm [GO:0005654]	DNA binding [GO:0003677]; LBD domain binding [GO:0050693]; nuclear estrogen receptor binding [GO:0030331]; nuclear receptor coactivator activity [GO:0030374]; nuclear retinoic acid receptor binding [GO:0042974]; nuclear thyroid hormone receptor binding [GO:0046966]; nuclear vitamin D receptor binding [GO:0042809]; peroxisome proliferator activated receptor binding [GO:0042975]; promoter-specific chromatin binding [GO:1990841]; protein-containing complex binding [GO:0044877]; transcription coactivator binding [GO:0001223]; transcription corepressor activity [GO:0003714]

			involved in pregnancy [GO:0060745]; mammary gland branching involved in thelarche [GO:0060744]; megakaryocyte development [GO:0035855]; monocyte differentiation [GO:0030224]; mrna transcription by RNA polymerase II [GO:0042789]; negative regulation of apoptotic process [GO:0043066]; negative regulation of keratinocyte proliferation [GO:0010839]; negative regulation of neuron differentiation [GO:0045665]; negative regulation of transcription by RNA polymerase II [GO:0000122]; nuclear receptor-mediated steroid hormone signaling pathway [GO:0030518]; peroxisome proliferator activated receptor signaling pathway [GO:0035357]; positive regulation of erythrocyte differentiation [GO:0045648]; positive regulation of G0 to G1 transition [GO:0070318]; positive regulation of gene expression [GO:0010628]; positive regulation of hepatocyte proliferation [GO:2000347]; positive regulation of intracellular estrogen receptor signaling pathway [GO:0033148]; positive regulation of keratinocyte differentiation [GO:0045618]; positive regulation of transcription initiation by RNA polymerase II [GO:0060261]; positive regulation of type II interferon-mediated signaling pathway [GO:0060335]; protein import into nucleus [GO:0006606]; regulation of vitamin D receptor signaling pathway [GO:0070562]; retinal pigment epithelium development [GO:0003406]; thyroid hormone generation [GO:0006590]; thyroid hormone receptor signaling pathway [GO:0002154]; ventricular trabecula myocardium morphogenesis [GO:0003222]	
A0A8I3Q1Y6	Mediator of RNA polymerase II	SMIM7	Regulation of transcription by RNA polymerase II [GO:0006357]	Mediator complex [GO:0016592]

	transcription subunit 26 (Cofactor required for Sp1 transcriptional activation subunit 7) (Mediator complex subunit 26)				
A0A8I3Q4H4	Metalloproteinase inhibitor 1 (Tissue inhibitor of metalloproteinases 1)	TIMP1	Cellular response to UV-A [GO:0071492]; connective tissue replacement involved in inflammatory response wound healing [GO:0002248]; negative regulation of membrane protein ectodomain proteolysis [GO:0051045]; negative regulation of trophoblast cell migration [GO:1901164]; positive regulation of cell population proliferation [GO:0008284]; regulation of integrin-mediated signaling pathway [GO:2001044]	Basement membrane [GO:0005604]; extracellular space [GO:0005615]	Cytokine activity [GO:0005125]; growth factor activity [GO:0008083]; metalloendopeptidase inhibitor activity [GO:0008191]; zinc ion binding [GO:0008270]
A0A8I3RQP4	Microtubule associated protein 1B	MAP1B	Axon extension [GO:0048675]; dendrite development [GO:0016358]; establishment of monopolar cell polarity [GO:0061162]; microtubule bundle formation [GO:0001578]; mitochondrion transport along microtubule [GO:0047497]; negative regulation of intracellular transport [GO:0032387]; odontoblast differentiation [GO:0071895]; regulation of postsynapse assembly [GO:0150052]	Cytosol [GO:0005829]; dendrite [GO:0030425]; glutamatergic synapse [GO:0098978]; microtubule [GO:0005874]; neuronal cell body [GO:0043025]; photoreceptor outer segment [GO:0001750]; plasma membrane [GO:0005886]; postsynaptic density [GO:0014069]	Microtubule binding [GO:0008017]
A0A8I3RQY7	Modulator of smoothened	MOSMO	Embryonic limb morphogenesis [GO:0030326]; embryonic skeletal system development [GO:0048706]; gene expression [GO:0010467]; heart development [GO:0007507]; in utero embryonic development [GO:0001701]; left/right pattern formation [GO:0060972]; lung development [GO:0030324]; negative regulation of smoothened signaling pathway [GO:0045879]; protein localization [GO:0008104]; regulation of neuron differentiation [GO:0045664]; regulation of	Ciliary membrane [GO:0060170]; Golgi apparatus [GO:0005794]	

			protein stability [GO:0031647]; smoothened signaling pathway [GO:0007224]		
A0A8I3P6H2	Myeloperoxidase	MPO	Response to oxidative stress [GO:0006979]		Heme binding [GO:0020037]; peroxidase activity [GO:0004601]
A0A8I3PHW8	N-acetylated alpha-linked acidic dipeptidase 2	NAALAD2		Plasma membrane [GO:0005886]	
A0A8I3MTC5	NCCRP1, F-box associated domain containing	NCCRP1	Positive regulation of cell population proliferation [GO:0008284]	Cytosol [GO:0005829]	
A0A8I3N3R2	Neurexin 1	NRXN1	Cell adhesion [GO:0007155]	Presynaptic membrane [GO:0042734]	
A0A8I3P8P3	Neuropilin	NRP2	Angiogenesis [GO:0001525]; axon guidance [GO:0007411]	Membrane [GO:0016020]	Heparin binding [GO:0008201]; metal ion binding [GO:0046872]; semaphorin receptor activity [GO:0017154]; vascular endothelial growth factor receptor activity [GO:0005021]
A0A8I3QYU2	Uncharacterized protein	NIPSNAP1	Mitophagy [GO:0000423]; sensory perception of pain [GO:0019233]	Mitochondrion [GO:0005739]; synaptic membrane [GO:0097060]	
A0A8I3N8P2	NLR family pyrin domain containing 14	NLRP14	Spermatogenesis [GO:0007283]	Cytoplasm [GO:0005737]	
A0A8I3NDM5	Focal adhesion kinase 1 (EC 2.7.10.2) (Protein-tyrosine kinase 2) (pp125fak)	PTK2	Positive regulation of cell population proliferation [GO:0008284]; regulation of biological quality [GO:0065008]; regulation of cellular component organization [GO:0051128]; regulation of multicellular organismal process [GO:0051239]; signal complex assembly [GO:0007172]	Cell cortex [GO:0005938]; cell projection [GO:0042995]; cytoskeleton [GO:0005856]; focal adhesion [GO:0005925]; plasma membrane [GO:0005886]	ATP binding [GO:0005524]; protein tyrosine kinase activity [GO:0004713]
A0A8I3PXZ8	Non-specific serine/threonine protein kinase (EC 2.7.11.1)	CDC42BPG		Cytoplasm [GO:0005737]	ATP binding [GO:0005524]; metal ion binding [GO:0046872]; protein serine/threonine kinase activity [GO:0004674]
A0A8I3PPA3	Non-specific serine/threonine protein kinase (EC 2.7.11.1)	KALRN	Adult locomotory behavior [GO:0008344]; habituation [GO:0046959]; lactation [GO:0007595]; maternal behavior [GO:0042711]; maternal process involved in parturition [GO:0060137]; memory [GO:0007613]; negative regulation of growth	Cytosol [GO:0005829]; nucleoplasm [GO:0005654]	ATP binding [GO:0005524]; guanyl-nucleotide exchange factor activity [GO:0005085]; protein serine/threonine kinase activity [GO:0004674]

			hormone secretion [GO:0060125]; neuromuscular junction development [GO:0007528]; positive regulation of dendritic spine morphogenesis [GO:0061003]; social behavior [GO:0035176]		
A0A8I3P7R0	Non-specific serine/threonine protein kinase (EC 2.7.11.1)	ULK2	Autophagy [GO:0006914]; regulation of autophagy [GO:0010506]	Phagophore assembly site membrane [GO:0034045]	ATP binding [GO:0005524]; protein serine/threonine kinase activity [GO:0004674]
A0A8I3NVN8	NPC intracellular cholesterol transporter 2 (Epididymal secretory protein E1)	LOC119876 012	Intracellular cholesterol transport [GO:0032367]	Extracellular region [GO:0005576]	Sterol binding [GO:0032934]
A0A8I3PDV0	NPC intracellular cholesterol transporter 2 (Epididymal secretory protein E1)		Intracellular cholesterol transport [GO:0032367]	Extracellular region [GO:0005576]	Sterol binding [GO:0032934]
A0A8I3NGM3	Nuclear receptor binding SET domain protein 1	NSD1	Methylation [GO:0032259]	Chromosome [GO:0005694]; nucleus [GO:0005634]	Histone H3 methyltransferase activity [GO:0140938]; metal ion binding [GO:0046872]; protein-lysine N-methyltransferase activity [GO:0016279]
A0A8I3MNL8	Nuclear receptor corepressor 1	NCOR1	Chromatin organization [GO:0006325]; negative regulation of DNA-templated transcription [GO:0045892]	Nucleoplasm [GO:0005654]; protein-containing complex [GO:0032991]	DNA binding [GO:0003677]
A0A8I3Q4R4	U8 snorna-decapping enzyme (EC 3.6.1.62) (EC 3.6.1.64) (IDP phosphatase) (Inosine diphosphate phosphatase) (Nucleoside diphosphate-linked moiety X motif 16)	NUDT16	Nucleotide metabolic process [GO:0009117]	Nucleolus [GO:0005730]; nucleoplasm [GO:0005654]	RNA binding [GO:0003723]

	(m7gpppn-mrna hydrolase)				
A0A8I3NLX2	Olfactory receptor	LOC119880303		Plasma membrane [GO:0005886]	G protein-coupled receptor activity [GO:0004930]; olfactory receptor activity [GO:0004984]
A0A8I3NAF1	Olfactory receptor	OR13E1		Plasma membrane [GO:0005886]	G protein-coupled receptor activity [GO:0004930]; olfactory receptor activity [GO:0004984]
A0A8I3P0A5	Trypsin (EC 3.4.21.4)	LOC475521	Proteolysis [GO:0006508]	Extracellular region [GO:0005576]	Serine-type endopeptidase activity [GO:0004252]
A0A8I3MX03	Uncharacterized protein	DHODH	Proteolysis [GO:0006508]	Extracellular region [GO:0005576]	Hemoglobin binding [GO:0030492]; serine-type endopeptidase activity [GO:0004252]
A0A8I3NVG7	Peptidyl-prolyl cis-trans isomerase (ppiase) (EC 5.2.1.8)		Apoptotic process [GO:0006915]; protein folding [GO:0006457]	Cytoplasm [GO:0005737]; extracellular region [GO:0005576]; nucleus [GO:0005634]	Peptidyl-prolyl cis-trans isomerase activity [GO:0003755]
A0A8I3Q354	Thioredoxin-dependent peroxiredoxin (EC 1.11.1.24)	PRDX4			Antioxidant activity [GO:0016209]; oxidoreductase activity [GO:0016491]
A0A8I3PG86	Peroxiredoxin-1 (EC 1.11.1.24)	PRDX1			Peroxiredoxin activity [GO:0051920]
A0A8I3PI24	Peroxiredoxin-5 (EC 1.11.1.24)		Cellular response to oxidative stress [GO:0034599]		Thioredoxin peroxidase activity [GO:0008379]
A0A8I3P259	Phosphatidylethanolamine binding protein 4	PEBP4			
A0A8I3Q1U5	Phosphatidylethanolamine-binding protein 1 (hcnppp)	PEBP1			
A0A8I3PDR7	Phosphatidylinositol-3,4,5-trisphosphate dependent Rac exchange factor 2	PREX2	Intracellular signal transduction [GO:0035556]		Guanyl-nucleotide exchange factor activity [GO:0005085]
A0A8I3P9Y9	Phosphoglycerate kinase (EC 2.7.2.3)	PGK1	Canonical glycolysis [GO:0061621]; cellular response to hypoxia [GO:0071456]; epithelial cell differentiation [GO:0030855]; gluconeogenesis [GO:0006094]; negative	Cytosol [GO:0005829]; extracellular space [GO:0005615]; membrane raft [GO:0045121]	ATP binding [GO:0005524]; phosphoglycerate kinase activity [GO:0004618]; protein-disulfide reductase (NAD(P)H) activity [GO:0047134]

			regulation of angiogenesis [GO:0016525]; plasminogen activation [GO:0031639]		
A0A8I3MVM5	Phosphoglycerate kinase (EC 2.7.2.3)	PGK2	Glycolytic process [GO:0006096]	Cytoplasm [GO:0005737]	ATP binding [GO:0005524]; phosphoglycerate kinase activity [GO:0004618]
A0A8I3SC19	Phosphoglycerate mutase (EC 5.4.2.11) (EC 5.4.2.4)	PGAM1	Gluconeogenesis [GO:0006094]; glycolytic process [GO:0006096]	Cytosol [GO:0005829]	Phosphoglycerate mutase activity [GO:0004619]
A0A8I3NE41	Phosphoglycerate mutase (EC 5.4.2.11) (EC 5.4.2.4)	PGAM2	Glycolytic process [GO:0006096]		Intramolecular phosphotransferase activity [GO:0016868]
A0A8I3QXI0	Phospholipase A2 (EC 3.1.1.4)	PLA2G4B	Glycerophospholipid catabolic process [GO:0046475]	Cytosol [GO:0005829]; membrane [GO:0016020]	Calcium ion binding [GO:0005509]; calcium-dependent phospholipase A2 activity [GO:0047498]
A0A8I3PS86	Phospholipid-transporting atpase (EC 7.6.2.1)	ATP9A	Negative regulation of exosomal secretion [GO:1903542]; phospholipid transport [GO:0015914]; regulation of endocytic recycling [GO:2001135]; regulation of retrograde transport, endosome to Golgi [GO:1905279]	Early endosome membrane [GO:0031901]; late endosome [GO:0005770]; perinuclear region of cytoplasm [GO:0048471]; plasma membrane [GO:0005886]; recycling endosome membrane [GO:0055038]; trans-Golgi network membrane [GO:0032588]	ATP binding [GO:0005524]; ATP hydrolysis activity [GO:0016887]; atpase-coupled intramembrane lipid transporter activity [GO:0140326]; magnesium ion binding [GO:0000287]; protease binding [GO:0002020]
A0A8I3PVT9	Phosphopyruvate hydratase (EC 4.2.1.11) (2-phospho-D-glycerate hydro-lyase)	ENO1	Glycolytic process [GO:0006096]	Phosphopyruvate hydratase complex [GO:0000015]	Magnesium ion binding [GO:0000287]; phosphopyruvate hydratase activity [GO:0004634]
A0A8I3NS61	Plastin-3 (T-plastin)	PLS3	Actin filament bundle assembly [GO:0051017]	Cytoplasm [GO:0005737]	Actin filament binding [GO:0051015]; calcium ion binding [GO:0005509]
A0A8I3PIJ3	Platelet-derived growth factor subunit B (PDGF-2) (Platelet-derived growth factor B chain) (Platelet-derived growth	PDGFB	Positive regulation of cell division [GO:0051781]; positive regulation of cell population proliferation [GO:0008284]	Membrane [GO:0016020]	Growth factor activity [GO:0008083]

	factor beta polypeptide)				
A0A8I3PEC2	Pleckstrin	PLEK	Cortical actin cytoskeleton organization [GO:0030866]; hematopoietic progenitor cell differentiation [GO:0002244]; integrin-mediated signaling pathway [GO:0007229]; intracellular signal transduction [GO:0035556]; negative regulation of G protein-coupled receptor signaling pathway [GO:0045744]; negative regulation of inositol phosphate biosynthetic process [GO:0010920]; phosphatidylinositol metabolic process [GO:0046488]; phospholipase C-activating G protein-coupled receptor signaling pathway [GO:0007200]; phospholipase C-inhibiting G protein-coupled receptor signaling pathway [GO:0030845]; platelet aggregation [GO:0070527]; positive regulation of actin filament bundle assembly [GO:0032233]; positive regulation of actin filament depolymerization [GO:0030836]; positive regulation of integrin activation [GO:0033625]; positive regulation of platelet activation [GO:0010572]; protein secretion by platelet [GO:0070560]; regulation of cell diameter [GO:0060305]; ruffle organization [GO:0031529]; thrombin-activated receptor signaling pathway [GO:0070493]; vesicle docking involved in exocytosis [GO:0006904]	Cytoplasm [GO:0005737]; ruffle membrane [GO:0032587]	Phosphatidylinositol-3,4-bisphosphate binding [GO:0043325]; protein homodimerization activity [GO:0042803]; protein kinase C binding [GO:0005080]
A0A8I3NSK4	Poly(A) binding protein cytoplasmic 1 like	PABPC1L			
A0A8I3N997	Polyadenylate-binding protein (PABP)	PABPC4		Cytoplasm [GO:0005737]	RNA binding [GO:0003723]
A0A8I3Q921	Polysaccharide biosynthesis domain containing 1	PBDC1			
A0A8I3PKK0	Potassium voltage-gated channel	KCNQ2		Voltage-gated potassium channel complex [GO:0008076]	Voltage-gated potassium channel activity [GO:0005249]

	subfamily Q member 2				
A0A8I3N9K7	Peptidylprolyl isomerase (EC 5.2.1.8)	PPIA	Apoptotic process [GO:0006915]; protein folding [GO:0006457]	Cytoplasm [GO:0005737]; extracellular region [GO:0005576]	Peptidyl-prolyl cis-trans isomerase activity [GO:0003755]
A0A8I3NE98	Prickle planar cell polarity protein 2	PRICKLE2		Cytoplasm [GO:0005737]	Zinc ion binding [GO:0008270]
Q9GL24	Procathepsin L (EC 3.4.22.15) (Cathepsin L1) [Cleaved into: Cathepsin L; Cathepsin L heavy chain; Cathepsin L light chain]	CTSL CTSL1	Antigen processing and presentation of peptide antigen [GO:0048002]; CD4-positive, alpha-beta T cell lineage commitment [GO:0043373]; collagen catabolic process [GO:0030574]; elastin catabolic process [GO:0060309]; enkephalin processing [GO:0034230]; immune response [GO:0006955]; positive regulation of apoptotic signaling pathway [GO:2001235]; protein autoprocessing [GO:0016540]; proteolysis involved in protein catabolic process [GO:0051603]; zymogen activation [GO:0031638]	Apical plasma membrane [GO:0016324]; chromaffin granule [GO:0042583]; extracellular space [GO:0005615]; lysosome [GO:0005764]	Cysteine-type endopeptidase activator activity involved in apoptotic process [GO:0008656]; cysteine-type endopeptidase activity [GO:0004197]; endopeptidase activity [GO:0004175]; metal ion binding [GO:0046872]
A0A8I3PG73	Procollagen-proline 3-dioxygenase (EC 1.14.11.7)	P3H2	Collagen metabolic process [GO:0032963]; negative regulation of cell population proliferation [GO:0008285]	Basement membrane [GO:0005604]; cytosol [GO:0005829]; endoplasmic reticulum [GO:0005783]; Golgi apparatus [GO:0005794]; nucleoplasm [GO:0005654]	Iron ion binding [GO:0005506]; L-ascorbic acid binding [GO:0031418]; procollagen-proline 3-dioxygenase activity [GO:0019797]
A0A8I3MSY8	Profilin	PFN1	Actin cytoskeleton organization [GO:0030036]; modification of postsynaptic actin cytoskeleton [GO:0098885]; modulation of chemical synaptic transmission [GO:0050804]; negative regulation of actin filament polymerization [GO:0030837]; negative regulation of stress fiber assembly [GO:0051497]; neural tube closure [GO:0001843]; positive regulation of actin filament polymerization [GO:0030838]; positive regulation of epithelial cell migration [GO:0010634]; positive regulation of ruffle assembly [GO:1900029]; protein stabilization [GO:0050821]; regulation of transcription by RNA polymerase II [GO:0006357]; synapse maturation [GO:0060074]	Cell cortex [GO:0005938]; cytoskeleton [GO:0005856]; glutamatergic synapse [GO:0098978]; nucleus [GO:0005634]	Actin monomer binding [GO:0003785]; adenyl-nucleotide exchange factor activity [GO:0000774]; phosphatidylinositol-4,5-bisphosphate binding [GO:0005546]; phosphotyrosine residue binding [GO:0001784]; proline-rich region binding [GO:0070064]; small gtpase binding [GO:0031267]

A0A8I3P4J7	Prolactin-induced protein	PIP		Extracellular region [GO:0005576]	
A0A8I3NFT3	Prostaglandin reductase 2 (EC 1.3.1.48) (15-oxoprostaglandin 13-reductase)	PTGR2	Prostaglandin metabolic process [GO:0006693]	Cytoplasm [GO:0005737]	15-oxoprostaglandin 13-oxidase [NAD(P)+] activity [GO:0047522]
Q9XS65	Prostaglandin-H2 D-isomerase (EC 5.3.99.2) (Glutathione-independent PGD synthase) (Lipocalin-type prostaglandin-D synthase) (Prostaglandin-D2 synthase) (PGD2 synthase) (PGDS) (PGDS2)	PTGDS	Mast cell degranulation [GO:0043303]; prostaglandin biosynthetic process [GO:0001516]; regulation of circadian sleep/wake cycle, sleep [GO:0045187]	Extracellular region [GO:0005576]; extracellular space [GO:0005615]; Golgi apparatus [GO:0005794]; nuclear membrane [GO:0031965]; perinuclear region of cytoplasm [GO:0048471]; rough endoplasmic reticulum [GO:0005791]	Prostaglandin-D synthase activity [GO:0004667]; retinoid binding [GO:0005501]; small molecule binding [GO:0036094]
A0A8I3P0V3	Proteasome subunit alpha type	PSMA3	Ubiquitin-dependent protein catabolic process [GO:0006511]	Cytoplasm [GO:0005737]; nucleus [GO:0005634]; proteasome core complex, alpha-subunit complex [GO:0019773]	
A0A8I3NL81	Proteasome subunit alpha type	PSMA4	Ubiquitin-dependent protein catabolic process [GO:0006511]	Cytoplasm [GO:0005737]; nucleus [GO:0005634]; proteasome core complex, alpha-subunit complex [GO:0019773]	
A0A8I3MGS5	Proteasome subunit alpha type	PSMA5	Proteasome-mediated ubiquitin-dependent protein catabolic process [GO:0043161]	Cytoplasm [GO:0005737]; nucleus [GO:0005634]; proteasome core complex, alpha-subunit complex [GO:0019773]	
A0A8I3NUC6	Proteasome subunit beta	PSMB2	Proteasomal protein catabolic process [GO:0010498]	Cytoplasm [GO:0005737]; nucleoplasm [GO:0005654]; proteasome core complex [GO:0005839]	
A0A8I3PLV2	Protein AAR2 homolog (AAR2 splicing factor homolog)	AAR2			

A0A8I3N4K7	Protein associated with LIN7 2, MAGUK p55 family member	PALS2		Plasma membrane [GO:0005886]	
A0A8I3Q0F7	Protein disulfide-isomerase A6 (EC 5.3.4.1)	PDIA6		Cytosol [GO:0005829]; endoplasmic reticulum lumen [GO:0005788]; endoplasmic reticulum-Golgi intermediate compartment [GO:0005793]; extracellular space [GO:0005615]	Protein disulfide isomerase activity [GO:0003756]; protein-disulfide reductase activity [GO:0015035]
A0A8I3S5M8	Protein disulfide-isomerase (EC 5.3.4.1)	PDIA3	Cellular response to interleukin-7 [GO:0098761]; extrinsic apoptotic signaling pathway [GO:0097191]; peptide antigen assembly with MHC class I protein complex [GO:0002502]; platelet aggregation [GO:0070527]; positive regulation of extrinsic apoptotic signaling pathway [GO:2001238]	Cell surface [GO:0009986]; endoplasmic reticulum lumen [GO:0005788]; extracellular space [GO:0005615]; melanosome [GO:0042470]; Tapasin-erp57 complex [GO:0061779]	Identical protein binding [GO:0042802]; protein disulfide isomerase activity [GO:0003756]; protein-disulfide reductase activity [GO:0015035]
C0LQL0	Protein S100 (S100 calcium-binding protein)	S100A8			Calcium ion binding [GO:0005509]
A0A8I3MFK0	Protein S100 (S100 calcium-binding protein)	S100A9			Antioxidant activity [GO:0016209]; calcium ion binding [GO:0005509]
A0A8I3PNZ6	Protein wnt	WNT7A	Animal organ development [GO:0048513]; cell differentiation [GO:0030154]; system development [GO:0048731]; tissue development [GO:0009888]; Wnt signaling pathway [GO:0016055]	Extracellular region [GO:0005576]	Signaling receptor binding [GO:0005102]
A0A8I3PDN1	Protocadherin 17	PCDH17	Homophilic cell adhesion via plasma membrane adhesion molecules [GO:0007156]	Plasma membrane [GO:0005886]	Calcium ion binding [GO:0005509]
A0A8I3MGE1	Protocadherin beta 1	PCDHB1	Homophilic cell adhesion via plasma membrane adhesion molecules [GO:0007156]	Plasma membrane [GO:0005886]	Calcium ion binding [GO:0005509]
A0A8I3N9W8	Protein prune homolog 2 (BNIP2 motif-containing molecule at the C-terminal region 1)	PRUNE2	Apoptotic process [GO:0006915]	Cytoplasm [GO:0005737]	
A0A8I3Q1A8	Pseudouridine synthase 7 like	PUS7L	Pseudouridine synthesis [GO:0001522]		Pseudouridine synthase activity [GO:0009982]; RNA binding [GO:0003723]

A0A8I3ML02	Pyruvate kinase (EC 2.7.1.40)				ATP binding [GO:0005524]; kinase activity [GO:0016301]; magnesium ion binding [GO:0000287]; potassium ion binding [GO:0030955]; pyruvate kinase activity [GO:0004743]
A0A8I3PPL6	Rab GDP dissociation inhibitor		Protein transport [GO:0015031]; small gtpase-mediated signal transduction [GO:0007264]	Cytoplasm [GO:0005737]; membrane [GO:0016020]	Gtpase activator activity [GO:0005096]; Rab GDP-dissociation inhibitor activity [GO:0005093]
A0A8I3P5H6	RILP-like protein 2	RILPL2	Epithelial cell morphogenesis [GO:0003382]; protein transport from ciliary membrane to plasma membrane [GO:1903445]	Centrosome [GO:0005813]; cilium [GO:0005929]; cytosol [GO:0005829]; membrane [GO:0016020]	Identical protein binding [GO:0042802]; protein dimerization activity [GO:0046983]
A0A8I3MXE7	Ras-related protein Rab-2A		Protein transport [GO:0015031]; vesicle-mediated transport [GO:0016192]	Acrosomal vesicle [GO:0001669]; autophagosome membrane [GO:0000421]; endoplasmic reticulum membrane [GO:0005789]; endoplasmic reticulum-Golgi intermediate compartment membrane [GO:0033116]; Golgi membrane [GO:0000139]; melanosome [GO:0042470]	GTP binding [GO:0005525]; gtpase activity [GO:0003924]
A0A8I3MVS4	Receptor protein-tyrosine kinase (EC 2.7.10.1)	EPHA2	Axial mesoderm formation [GO:0048320]; blood vessel endothelial cell proliferation involved in sprouting angiogenesis [GO:0002043]; bone remodeling [GO:0046849]; branching involved in mammary gland duct morphogenesis [GO:0060444]; camp metabolic process [GO:0046058]; cell chemotaxis [GO:0060326]; defense response to Gram-positive bacterium [GO:0050830]; inflammatory response [GO:0006954]; intrinsic apoptotic signaling pathway in response to DNA damage [GO:0008630]; keratinocyte differentiation [GO:0030216]; lens fiber cell morphogenesis [GO:0070309]; mammary gland epithelial cell proliferation [GO:0033598]; negative regulation of angiogenesis [GO:0016525];	Cell surface [GO:0009986]; focal adhesion [GO:0005925]; lamellipodium [GO:0030027]; leading edge membrane [GO:0031256]; tight junction [GO:0070160]	ATP binding [GO:0005524]; ephrin receptor activity [GO:0005003]; growth factor binding [GO:0019838]

			negative regulation of chemokine production [GO:0032682]; negative regulation of lymphangiogenesis [GO:1901491]; neural tube development [GO:0021915]; neuron differentiation [GO:0030182]; notochord cell development [GO:0060035]; notochord formation [GO:0014028]; osteoblast differentiation [GO:0001649]; osteoclast differentiation [GO:0030316]; pericyte cell differentiation [GO:1904238]; positive regulation of bicellular tight junction assembly [GO:1903348]; positive regulation of cell migration [GO:0030335]; positive regulation of protein localization to plasma membrane [GO:1903078]; post-anal tail morphogenesis [GO:0036342]; protein localization to plasma membrane [GO:0072659]; regulation of blood vessel endothelial cell migration [GO:0043535]; regulation of cell adhesion mediated by integrin [GO:0033628]; regulation of ERK1 and ERK2 cascade [GO:0070372]; regulation of lamellipodium assembly [GO:0010591]; response to growth factor [GO:0070848]; skeletal system development [GO:0001501]; vasculogenesis [GO:0001570]		
A0A8I3PJ07	Receptor protein-tyrosine kinase (EC 2.7.10.1)	FLT3	Cell surface receptor protein tyrosine kinase signaling pathway [GO:0007169]	Membrane [GO:0016020]	ATP binding [GO:0005524]; metal ion binding [GO:0046872]; transmembrane receptor protein tyrosine kinase activity [GO:0004714]
A0A8I3Q0N8	Replication termination factor 2 (Replication termination factor 2 domain-containing protein 1)	RTF2	Cellular response to hydroxyurea [GO:0072711]; mitotic DNA replication termination [GO:1902979]; regulation of DNA stability [GO:0097752]	Nucleus [GO:0005634]; replication fork [GO:0005657]	DNA binding [GO:0003677]
A0A8I3MAJ8	Required for meiotic nuclear division 1 homolog	RMND1	Positive regulation of mitochondrial translation [GO:0070131]	Mitochondrion [GO:0005739]	

A0A8I3P569	Small monomeric gtpase (EC 3.6.5.2)	RERGL			GTP binding [GO:0005525]; gtpase activity [GO:0003924]
A0A8I3P6H9	Rho GDP dissociation inhibitor beta	ARHGDIB	Negative regulation of trophoblast cell migration [GO:1901164]; regulation of Rho protein signal transduction [GO:0035023]; Rho protein signal transduction [GO:0007266]	Cytosol [GO:0005829]	Gtpase activator activity [GO:0005096]; gtpase activity [GO:0003924]; Rho GDP-dissociation inhibitor activity [GO:0005094]; small gtpase binding [GO:0031267]
A0A8I3N9U7	Rho guanine nucleotide exchange factor 19	ARHGEF19	Regulation of actin cytoskeleton organization [GO:0032956]; wound healing [GO:0042060]		Guanyl-nucleotide exchange factor activity [GO:0005085]
A0A8I3PCR7	Ribosomal protein L24	RPL24		Cytoplasm [GO:0005737]; ribonucleoprotein complex [GO:1990904]; ribosome [GO:0005840]	
A0A8I3NEI5	Ring finger protein 213	RNF213	Immune system process [GO:0002376]	Cytoplasm [GO:0005737]	ATP hydrolysis activity [GO:0016887]; metal ion binding [GO:0046872]; ubiquitin-protein transferase activity [GO:0004842]
A0A8I3PSA0	Ropporin-1A	ROPN1		Motile cilium [GO:0031514]	
A0A8I3S289	Roundabout guidance receptor 2	ROBO2			
A0A8I3N3X3	RUN and FYVE domain containing 1	RUFY1	Early endosome to Golgi transport [GO:0034498]; endosomal vesicle fusion [GO:0034058]; protein transport [GO:0015031]; regulation of endocytosis [GO:0030100]; small gtpase-mediated signal transduction [GO:0007264]	Cytosol [GO:0005829]; early endosome membrane [GO:0031901]; nuclear speck [GO:0016607]	Metal ion binding [GO:0046872]; protein-macromolecule adaptor activity [GO:0030674]; SH2 domain binding [GO:0042169]; SH3 domain binding [GO:0017124]
A0A8I3MT21	Ryanodine receptor 2	RYR2	Calcium ion transport into cytosol [GO:0060402]; cardiac muscle contraction [GO:0060048]; cellular response to caffeine [GO:0071313]; detection of calcium ion [GO:0005513]; establishment of protein localization to endoplasmic reticulum [GO:0072599]; intracellular calcium ion homeostasis [GO:0006874]; positive regulation of sequestering of calcium ion [GO:0051284]; positive regulation of the force of heart contraction [GO:0098735]; regulation of atrial cardiac muscle cell action potential	Calcium channel complex [GO:0034704]; sarcoplasmic reticulum membrane [GO:0033017]; Z disc [GO:0030018]	Calcium ion binding [GO:0005509]; calmodulin binding [GO:0005516]; identical protein binding [GO:0042802]; protein kinase A catalytic subunit binding [GO:0034236]; protein kinase A regulatory subunit binding [GO:0034237]; ryanodine-sensitive calcium-release channel activity [GO:0005219]; suramin binding [GO:0043924]

			[GO:0098910]; regulation of AV node cell action potential [GO:0098904]; regulation of cardiac muscle contraction by calcium ion signaling [GO:0010882]; regulation of heart rate [GO:0002027]; regulation of SA node cell action potential [GO:0098907]; regulation of ventricular cardiac muscle cell action potential [GO:0098911]; release of sequestered calcium ion into cytosol by sarcoplasmic reticulum [GO:0014808]; response to muscle activity [GO:0014850]; response to muscle stretch [GO:0035994]; response to redox state [GO:0051775]; type B pancreatic cell apoptotic process [GO:0097050]		
A0A8I3MKE4	S100 calcium binding protein A8	S100A8			Calcium ion binding [GO:0005509]
A0A8I3PSD1	Secreted phosphoprotein 24 (Secreted phosphoprotein 2)	SPP2	Bone remodeling [GO:0046849]	Extracellular region [GO:0005576]	
A0A8I3NZ25	Senataxin	SETX	Cellular response to hydrogen peroxide [GO:0070301]; circadian rhythm [GO:0007623]; double-strand break repair [GO:0006302]; mrna splice site recognition [GO:0006376]; positive regulation of DNA-templated transcription initiation [GO:2000144]; positive regulation of neuron projection development [GO:0010976]; positive regulation of RNA splicing [GO:0033120]; positive regulation of termination of RNA polymerase II transcription, poly(A)-coupled [GO:2000806]; positive regulation of transcription by RNA polymerase II [GO:0045944]; termination of RNA polymerase II transcription [GO:0006369]	Cytoplasm [GO:0005737]; growth cone [GO:0030426]; intercellular bridge [GO:0045171]; nuclear body [GO:0016604]; nuclear chromosome [GO:0000228]	Helicase activity [GO:0004386]; identical protein binding [GO:0042802]; transcription termination site sequence-specific DNA binding [GO:0001147]
A0A8I3PGH2	Serine/threonine-protein phosphatase 2A activator (EC 5.2.1.8)	PTPA		Cytoplasm [GO:0005737]	Peptidyl-prolyl cis-trans isomerase activity [GO:0003755]; phosphatase activator activity [GO:0019211]

	(Phosphotyrosyl phosphatase activator)				
A0A8I3P1P3	Serpin family B member 1	SERPINB1	Negative regulation of cell migration [GO:0030336]; negative regulation of interleukin-1 beta production [GO:0032691]; type B pancreatic cell proliferation [GO:0044342]	Cytoplasmic ribonucleoprotein granule [GO:0036464]; extracellular space [GO:0005615]	Serine-type endopeptidase inhibitor activity [GO:0004867]
A0A8I3MHU9	Serpin family B member 8	SERPINB8		Extracellular space [GO:0005615]	Serine-type endopeptidase inhibitor activity [GO:0004867]
A0A8I3NIH9	Serpin H1 (Collagen-binding protein)	SERPINH1	Chondrocyte development involved in endochondral bone morphogenesis [GO:0003433]; collagen biosynthetic process [GO:0032964]; collagen fibril organization [GO:0030199]; protein maturation [GO:0051604]	Endoplasmic reticulum [GO:0005783]; endoplasmic reticulum-Golgi intermediate compartment [GO:0005793]; extracellular space [GO:0005615]; membrane raft [GO:0045121]	Collagen binding [GO:0005518]; serine-type endopeptidase inhibitor activity [GO:0004867]; unfolded protein binding [GO:0051082]
A0A8I3NFG5	SET and MYND domain-containing protein 4	SMYD4	Methylation [GO:0032259]	Cytoplasm [GO:0005737]; nucleus [GO:0005634]	Metal ion binding [GO:0046872]; methyltransferase activity [GO:0008168]
A0A8I3PD50	Phosphoinositide-3-kinase regulatory subunit 3	LOC100856339			
A0A8I3NPU4	SH3 and multiple ankyrin repeat domains 1	SHANK1	Adult behavior [GO:0030534]; associative learning [GO:0008306]; dendritic spine morphogenesis [GO:0060997]; determination of affect [GO:0050894]; habituation [GO:0046959]; long-term memory [GO:0007616]; negative regulation of actin filament bundle assembly [GO:0032232]; neuromuscular process controlling balance [GO:0050885]; olfactory behavior [GO:0042048]; positive regulation of excitatory postsynaptic potential [GO:2000463]; protein localization to synapse [GO:0035418]; righting reflex [GO:0060013]; social behavior [GO:0035176]; vocalization behavior [GO:0071625]	Dendritic spine [GO:0043197]; excitatory synapse [GO:0060076]; glutamatergic synapse [GO:0098978]; postsynaptic density [GO:0014069]; postsynaptic membrane [GO:0045211]; Schaffer collateral - CA1 synapse [GO:0098685]	Ionotropic glutamate receptor binding [GO:0035255]; structural constituent of postsynaptic density [GO:0098919]; synaptic receptor adaptor activity [GO:0030160]
A0A8I3N0Y2	Uncharacterized protein	SLC25A24	Transmembrane transport [GO:0055085]	Mitochondrial inner membrane [GO:0005743]	Calcium ion binding [GO:0005509]

A0A8I3MHQ5	Solute carrier family 49 member 3	SLC49A3		Membrane [GO:0016020]	Transmembrane transporter activity [GO:0022857]
A0A8I3NZI8	Sperm acrosome associated 9	SPACA9			
A0A8I3NJB6	Sperm acrosome membrane-associated protein 3	SPACA3	Fusion of sperm to egg plasma membrane involved in single fertilization [GO:0007342]; sperm-egg recognition [GO:0035036]	Acrosomal matrix [GO:0043159]; acrosomal membrane [GO:0002080]	Lysozyme activity [GO:0003796]
A0A8I3PFW1	Sperm equatorial segment protein 1	SPESP1	Acrosome reaction [GO:0007340]; fusion of sperm to egg plasma membrane involved in single fertilization [GO:0007342]	Acrosomal vesicle [GO:0001669]; cytosol [GO:0005829]; nucleoplasm [GO:0005654]	
A0A8I3MEV1	Sperm flagellar 2	SPEF2			
A0A8I3PVY9	Spermatogenesis associated serine rich 2 like	SPATS2L		Cytosol [GO:0005829]; nucleolus [GO:0005730]; nucleoplasm [GO:0005654]; protein-containing complex [GO:0032991]	
A0A8I3PA49	ST13 Hsp70 interacting protein	ST13			
A0A8I3NJ34	Star related lipid transfer domain containing 3	STARD3		Late endosome membrane [GO:0031902]	Lipid binding [GO:0008289]
A0A8I3S319	Strawberry notch homolog 1	SBNO1	Regulation of DNA-templated transcription [GO:0006355]		
A0A8I3PIQ1	Stress-70 protein, mitochondrial (75 kda glucose-regulated protein) (Heat shock 70 kda protein 9)	HSPA9			ATP binding [GO:0005524]; ATP-dependent protein folding chaperone [GO:0140662]; unfolded protein binding [GO:0051082]
A0A8I3MZG9	Sulfotransferase (EC 2.8.2.-)	SULT1C3	3'-phosphoadenosine 5'-phosphosulfate metabolic process [GO:0050427]; xenobiotic metabolic process [GO:0006805]		3'-phosphoadenosine 5'-phosphosulfate binding [GO:0050656]; alcohol sulfotransferase activity [GO:0004027]; aryl sulfotransferase activity [GO:0004062]
A0A8I3PS52	Superoxide dismutase [Cu-Zn] (EC 1.15.1.1)	SOD1		Nucleus [GO:0005634]	Copper ion binding [GO:0005507]; superoxide dismutase activity [GO:0004784]

A0A8I3N828	TANK binding kinase 1	TBK1		Cytoplasm [GO:0005737]	ATP binding [GO:0005524]; protein serine/threonine kinase activity [GO:0004674]
A0A8I3P3D6	T-complex protein 1 subunit theta (CCT-theta)	CCT8		Cell projection [GO:0042995]; centrosome [GO:0005813]; chaperonin-containing T-complex [GO:0005832]	ATP binding [GO:0005524]; ATP hydrolysis activity [GO:0016887]; ATP-dependent protein folding chaperone [GO:0140662]; unfolded protein binding [GO:0051082]
A0A8I3P505	Teneurin transmembrane protein 3	TENM3	Camera-type eye morphogenesis [GO:0048593]; signal transduction [GO:0007165]	Cell projection [GO:0042995]; plasma membrane [GO:0005886]	
A0A8I3PPJ8	Tetratricopeptide repeat and ankyrin repeat containing 1	TRANK1			
A0A8I3RU84	Tetratricopeptide repeat domain 16	TTC16			
A0A8I3NZH5	Tissue factor pathway inhibitor	TFPI2	Blood coagulation [GO:0007596]	Extracellular region [GO:0005576]	Serine-type endopeptidase inhibitor activity [GO:0004867]
A0A8I3PL38	Ig-like domain-containing protein	TTN			
A0A8I3MCL6	Trans-1,2-dihydrobenzene-1,2-diol dehydrogenase (EC 1.1.1.179) (EC 1.3.1.20) (D-xylose 1-dehydrogenase) (D-xylose-NADP dehydrogenase) (Dimeric dihydrodiol dehydrogenase)	DHDH			Nucleotide binding [GO:0000166]; oxidoreductase activity [GO:0016491]
A0A8I3P237	Transaldolase (EC 2.2.1.2)	TALDO1	Carbohydrate metabolic process [GO:0005975]; pentose-phosphate shunt, non-oxidative branch [GO:0009052]	Cytosol [GO:0005829]	Transaldolase activity [GO:0004801]
A0A8I3NWM9	Transforming growth factor beta receptor 3	TGFBR3		Extracellular region [GO:0005576]; plasma membrane [GO:0005886]	

A0A8I3MWF5	Transitional endoplasmic reticulum atpase (EC 3.6.4.6) (15S Mg(2+)-atpase p97 subunit) (Valosin-containing protein)	VCP	Autophagy [GO:0006914]; DNA repair [GO:0006281]	Cytosol [GO:0005829]; endoplasmic reticulum [GO:0005783]; nucleus [GO:0005634]	ATP binding [GO:0005524]; ATP hydrolysis activity [GO:0016887]; lipid binding [GO:0008289]
A0A8I3Q4C1	Transketolase (EC 2.2.1.1)	TKT		Cytoplasm [GO:0005737]	Transferase activity [GO:0016740]
A0A8I3PX87	Transmembrane protein 255B	TMEM255 B		Membrane [GO:0016020]	
A0A8I3N8D4	Transmembrane protein 63A	TMEM63A		Endomembrane system [GO:0012505]; membrane [GO:0016020]	Calcium-activated cation channel activity [GO:0005227]; nucleic acid binding [GO:0003676]
A0A8I3PRM7	Triosephosphate isomerase (EC 5.3.1.1)	TPI1	Canonical glycolysis [GO:0061621]; gluconeogenesis [GO:0006094]; glyceraldehyde-3-phosphate biosynthetic process [GO:0046166]	Cytosol [GO:0005829]	Lyase activity [GO:0016829]; protein homodimerization activity [GO:0042803]; triose-phosphate isomerase activity [GO:0004807]; ubiquitin protein ligase binding [GO:0031625]
A0A8I3PNE5	TSC22 domain family member 2	TSC22D2	Negative regulation of cell cycle [GO:0045786]; regulation of transcription by RNA polymerase II [GO:0006357]; response to osmotic stress [GO:0006970]		
A0A8I3MD95	Tubulin beta chain	TUBB3	Axon guidance [GO:0007411]; dorsal root ganglion development [GO:1990791]; microtubule cytoskeleton organization [GO:0000226]	Cell periphery [GO:0071944]; cytoplasm [GO:0005737]; dendrite [GO:0030425]; filopodium [GO:0030175]; growth cone [GO:0030426]; intercellular bridge [GO:0045171]; lamellipodium [GO:0030027]; microtubule [GO:0005874]; mitotic spindle [GO:0072686]; neuronal cell body [GO:0043025]	GTP binding [GO:0005525]; gtpase activity [GO:0003924]; netrin receptor binding [GO:1990890]; structural constituent of cytoskeleton [GO:0005200]
A0A8I3NWL8	Ubiquitin carboxyl-terminal hydrolase 47 (EC 3.4.19.12) (Deubiquitinating enzyme 47) (Ubiquitin	USP47	Protein deubiquitination [GO:0016579]; proteolysis [GO:0006508]		Cysteine-type deubiquitinase activity [GO:0004843]

	thioesterase 47) (Ubiquitin-specific-processing protease 47)				
A0A8I3MJ82	Ubiquitin conjugating enzyme E2 D2	UBE2D2	Protein autoubiquitination [GO:0051865]; protein K48-linked ubiquitination [GO:0070936]	Protein-containing complex [GO:0032991]	ATP binding [GO:0005524]; ubiquitin conjugating enzyme activity [GO:0061631]; ubiquitin protein ligase activity [GO:0061630]
A0A8I3RSZ1	Ubiquitin protein ligase E3 component n-recognin 4	UBR4	Cytoplasm protein quality control by the ubiquitin-proteasome system [GO:0071629]; endosome organization [GO:0007032]; negative regulation of fatty acid biosynthetic process [GO:0045717]; negative regulation of HRI-mediated signaling [GO:0141191]; positive regulation of autophagy [GO:0010508]; protein branched polyubiquitination [GO:0141198]; protein K11-linked ubiquitination [GO:0070979]; protein K27-linked ubiquitination [GO:0044314]; protein K48-linked ubiquitination [GO:0070936]; protein stabilization [GO:0050821]; response to oxidative stress [GO:0006979]; ubiquitin-dependent protein catabolic process via the N-end rule pathway [GO:0071596]	Centrosome [GO:0005813]; cytosol [GO:0005829]; endosome [GO:0005768]; nucleoplasm [GO:0005654]	Calmodulin binding [GO:0005516]; ubiquitin protein ligase activity [GO:0061630]; zinc ion binding [GO:0008270]
A0A8I3NFB9	Ubiquitin-ribosomal protein es31 fusion protein	RPS27A	Translation [GO:0006412]	Cytoplasm [GO:0005737]; nucleus [GO:0005634]; ribonucleoprotein complex [GO:1990904]; ribosome [GO:0005840]	Structural constituent of ribosome [GO:0003735]
A0A8I3MWJ2	DNA damage inducible 1 homolog 1	DDI1	Cellular response to hydroxyurea [GO:0072711]; proteasomal protein catabolic process [GO:0010498]; regulation of DNA stability [GO:0097752]; regulation of protein stability [GO:0031647]		Aspartic-type endopeptidase activity [GO:0004190]
A0A8I3MXY6	Uncharacterized protein	LOC100685053			
A0A8I3NG71	Uncharacterized protein	ASAP1		Cytoplasm [GO:0005737]	Gtpase activator activity [GO:0005096]; metal ion binding [GO:0046872]

A0A8I3NWY4	Uncharacterized protein	LOC609513			
A0A8I3Q8J7	Uncharacterized protein	FSIP2L			
A0A8I3N1M4	RAB11B, member RAS oncogene family	RAB11B	Protein transport [GO:0015031]		GTP binding [GO:0005525]; gtpase activity [GO:0003924]
A0A8I3PMK8	Uncharacterized protein	ROPN1		Motile cilium [GO:0031514]	
A0A8I3MWR5	Uncharacterized protein	WDR27			
A0A8I3NX52	Uncharacterized protein				
A0A8I3N7I6	Uncharacterized protein			Membrane [GO:0016020]	
A0A8I3MHY1	UPAR/Ly6 domain-containing protein	CD177		Plasma membrane [GO:0005886]	
A0A8I3PHJ9	Uroporphyrinogen decarboxylase (EC 4.1.1.37)	UROD	Heme A biosynthetic process [GO:0006784]; heme B biosynthetic process [GO:0006785]; heme O biosynthetic process [GO:0048034]; porphyrin-containing compound catabolic process [GO:0006787]; protoporphyrinogen IX biosynthetic process [GO:0006782]	Cytosol [GO:0005829]; nucleoplasm [GO:0005654]	Uroporphyrinogen decarboxylase activity [GO:0004853]
A0A8I3NEM4	Utrophin	UTRN		Cytoplasm [GO:0005737]; cytoskeleton [GO:0005856]; plasma membrane bounded cell projection [GO:0120025]; postsynaptic membrane [GO:0045211]	Actin binding [GO:0003779]; zinc ion binding [GO:0008270]
Q5TJF0	Vacuolar protein sorting-associated protein 52 homolog	VPS52	Endocytic recycling [GO:0032456]; Golgi to vacuole transport [GO:0006896]; lysosomal transport [GO:0007041]; protein transport [GO:0015031]; retrograde transport, endosome to Golgi [GO:0042147]	Cytosol [GO:0005829]; EARP complex [GO:1990745]; endosome membrane [GO:0010008]; GARP complex [GO:0000938]; recycling endosome [GO:0055037]	Syntaxin binding [GO:0019905]
A0A8I3NNR9	Vimentin	VIM		Intermediate filament [GO:0005882]; nuclear matrix [GO:0016363]	
A0A8I3PJ06	YEATS domain containing 2	YEATS2	Regulation of DNA-templated transcription [GO:0006355]	Nucleus [GO:0005634]	
A0A8I3Q7X0	YTH domain-containing family protein	YTHDF3	Mrna destabilization [GO:0061157]; negative regulation of type I interferon-mediated signaling pathway [GO:0060339]; positive	Cytoplasmic stress granule [GO:0010494]; P-body [GO:0000932]	N6-methyladenosine-containing RNA reader activity [GO:1990247];

			regulation of translational initiation [GO:0045948]; regulation of trophoblast cell migration [GO:1901163]; stress granule assembly [GO:0034063]		ribosome binding [GO:0043022]; RNA binding [GO:0003723]
A0A8I3MUN4	ZFP28 zinc finger protein	ZFP28	Regulation of DNA-templated transcription [GO:0006355]		DNA binding [GO:0003677]; metal ion binding [GO:0046872]
A0A8I3MKQ0	Zinc finger protein 592	ZNF592		Nucleus [GO:0005634]	DNA binding [GO:0003677]
A0A8I3NDP5	Zinc finger protein 629	ZNF629		Nucleus [GO:0005634]	
A0A8I3NH30	Zinc finger protein 804B	ZNF804B			Metal ion binding [GO:0046872]
A0A8I3NIV3	Zinc finger SWIM-type containing 6	ZSWIM6			Zinc ion binding [GO:0008270]
A0A8I3NBY9	Uncharacterized protein	ZPBP	Binding of sperm to zona pellucida [GO:0007339]	Acrosomal vesicle [GO:0001669]; extracellular region [GO:0005576]	
